# Rare Earths-Doped and Ceria-Coated Strontium Aluminate Platelets—Versatile Luminescent Platforms for Correlated Lifetime Imaging by Multiphoton FLIM and PLIM

**DOI:** 10.1021/acsomega.5c01649

**Published:** 2025-04-29

**Authors:** David G. Calatayud, María Victoria Martín Arroyo, Amador C. Caballero, Marina Villegas, Haobo Ge, Stanley W. Botchway, Sofia I. Pascu, Marco Peiteado, Teresa Jardiel

**Affiliations:** † Electroceramics Department, Instituto de Cerámica y Vidrio—CSIC, Kelsen 5, Campus de Cantoblanco, 28049 Madrid, Spain; ‡ Inorganic Chemistry, 152670 Universidad Autonoma de Madrid, Francisco Tomas y Valiente 7, Campus de Cantoblanco, 28049 Madrid, Spain; § Department of Chemistry, 1555 University of Bath, BA2 7AY Bath, U.K.; ∥ STFC Research Complex at Harwell, Rutherford Appleton Laboratory, Harwell, Science and Innovation Campus, Harwell, Oxfordshire OX11 0QX, U.K.

## Abstract

We report our recent advances in the design and synthesis of functional and hybrid composite nanomaterials with properties geared toward life sciences assays and as platforms for biomedical imaging applications. Using a stepwise reverse micelle procedure, we synthesized hybrid platelets comprising rare earth-doped strontium aluminate cores labeled Eu,Dy:SrAlO, where the phase nominally denoted as Sr_0_._95_Eu_0_._02_Dy_0_._03_Al_2_O_4_ dominates the nature of the composite, as demonstrated by extensive X-ray diffraction investigations. These were coated with a biocompatible cerium oxide shell, giving rise to the hierarchical hybrids denoted CeO_2_@Eu,Dy:SrAlO. Such Eu/Dy codoped strontium aluminates exhibit broad luminescent emissions with high optical sensitivity. The CeO_2_ shell further imparts biocompatibility and water dispersibility, resulting in kinetically stable nanoplatelets which can translocate into living cells in lifetime imaging protocols that were optimized for imaging across nano- and microscales. Multiphoton fluorescence lifetime imaging microscopy (MP FLIM) confirmed the luminescent properties in thin films and living cellular environments. These nanohybrids represent a significant step forward in the development of functional molecules and materials, leveraging directed and self-assembly strategies for their synthesis. Their luminescence (detectable by fluorescence as well as phosphorescence emission intensity correlated with emission lifetime), negligible toxicity on the time scale of imaging assays and up to 72 h, and biocompatibility with cellular milieu enabled their tracing with living cells. Their cellular activity was estimated by standard MTT assays in PC-3 and provided a further insight into their behavior in biological environments. The inclusion of heavy cerium and strontium atoms enhanced X-ray attenuation, supporting multimodal imaging by integrating optical and X-ray-based methods, which paves the way for potential applications in computed tomography correlated to confocal microscopy coupled with fluorescence lifetime imaging. These findings highlight the versatility of these luminescent hybrids for bioimaging and as synthetic scaffolds toward nanomedicine applications, bridging advanced imaging modalities with functional materials design.

## Graphical abstract

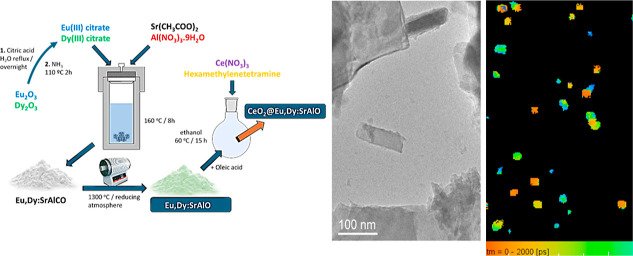

## Introduction

Biomedical imaging approaches and tools are imperative for a greater understanding of new diagnostic probes. There is a need to develop new sustainable technologies for such functional materials as well as design and synthesis to provide access to more sensitive tools for better and earlier detection of different pathologies. It is in this context that nanomedicine, defined as the application of nanotechnology to healthcare, has emerged to provide new solutions to the unsolved problems of current medicine.^1,2^ The main objectives in nanomedicine are the development of new therapy and diagnosis systems as well as to improve the existing ones.^3–5^ In the field of diagnostics, medical images are widely used for their potential toward easy-to-understand interface, allowing the creation of visual representations of a region inside single cells and the whole body.^6^ A wide range of imaging modalities can be enumerated, such as optical fluorescence imaging (OI), magnetic resonance imaging, or computed tomography (CT), among others.^7^ All of them have their own advantages and limitations, including varying resolution (spatial and/or temporal), sensitivity, quantitative accuracy, and different postchemotherapy/postradiation lesions, but none alone is able to obtain all of the essential information across different length scales (from molecules to tissues and organs) required for complete analysis.^8^ Consequently, many efforts are currently directed toward the production of so-called multimodal imaging platforms, in which complementary techniques are combined in a single platform containing different contrast agents (CAs).^9,10^ CAs are mostly chemical compounds that are introduced into cell and the body to provide an image in which contrast and spatial resolution are enhanced by magnifying signal differences between adjacent regions and tissues.^6^ CAs can also be functionalized by adding certain group functions, for example, to target a specific tissue.^11^ The combination of two or more CAs on a single platform can bring important benefits, for example, obtaining the maximum information from complementary imaging techniques in a single visit to the doctor (i.e., administering the CA only once). In this context, a particularly advantageous scenario is the possibility of constructing bimodal platforms combining fluorescence optical imaging and CT activity: CT has high resolution over a large (centimeter) range but low sensitivity and could be complemented by the higher cellular level sensitivity of fluorescence/optical imaging.

However, the CAs currently in use for both imaging modalities display serious limitations particularly toward such bimodal merging. On the one hand, the preferred CA option in the case of optical imaging involves the use of organic fluorescent dyes, which may present relatively low signals, poor photostability, and a broad emission spectrum, which is limiting in multichannel assays.^12^ Quantum dots (QDs) with tunable fluorescent spectra have been used, as they provide sharp emission bands (few nm) and higher resistance to photobleaching. The most promising ones are those based on CdSe and CdTe,^12,13^ although the undesirable presence of toxic cadmium (with detrimental effects on most organs^14^), together with complicated surface chemistry both *in vitro* and *in vivo* and very low kinetic stability in biological environments,^12^ hinders their practical uses. Alternatively, inorganic ceramic nanoparticles with luminescent properties have the potential to overcome the limitations of existing CAs.^15^ Like QDs, they are largely resistant to photodegradation and capable of supporting multimodal activity. However, their surface is easier to modify to make them biocompatible, keeping toxicity low, and to functionalize for tissue targeting and to reduce nonspecific interactions with biomolecules in living cells.^8,16^ Mostly based on doped oxide-based matrices, the emission of these inorganic nanostructures relies on the presence of specific metal ions that act as dopant activators and so they can be designed to emit at a wavelength of interest and to elude cellular and tissue autofluorescence issues.^17^ With a promising electronic configuration that gives rise to fine, sharp emission lines related to specific transitions, lanthanides (Ln^3+^) become the most suitable metal ions in these structures, while vanadates, phosphates, or aluminates are representative host matrix materials leading to intense emission.^17,19–22^ Recently we have succeeded in preparing core–shell structures consisting of Eu^2+^, Dy^3+^ codoped Sr_4_Al_14_O_25_ particles using a ball-milling technique followed by encapsulation within a well-known biocompatible silica layer which showed potential for application in biomedical diagnosis.^18^

Additionally, the CAs commonly used in CT rely upon high X-ray absorption and the production of sharp cross-sectional images^23^ and the high number of doses necessary to achieve good contrast in imaging often cause serious metabolic side effects such as, for example, in case of CT with medically ubiquitous iodine-based agents, thyrotoxic crisis, or hyperthyroidism.^24,25^ Barium-based CAs have been tested instead but they have shown a poor specificity for inflammatory sites that prevents their use in patients with inflammatory bowel disease (IBD).^26^ Gold-, bismuth-, tantalum-, or lanthanide-based nanoparticles have also attracted interest in recent years.^25–28^ They are all characterized by effective X-ray absorption and display longer blood circulation time, allowing their use in lower doses. Au nanoparticles, in particular, can accumulate longer in damaged tissues and are easy to remove when coated with biodegradable coatings that break down into harmless byproducts after use.^25^ In contrast, cerium oxide-based nanoparticles (denoted ceria NPs) are more promising in addressing certain conditions, e.g., those associated with imaging IBD: because Ce (*Z* = 58) has an even higher atomic number than iodine (*Z* = 53), it is well suited to use with the same X-ray setup currently used in medical imaging, leading to improved image quality in CT scans.^26,29,30^

A state-of-the-art procedure includes several reports toward the preparation of a bimodal OI/CT combination using different CA: Zhang and co-workers prepared a noninvasive dual nanoprobe for in vivo tumor imaging using gold nanoparticles as a CA for CT and organic fluorescence dyes for optical imaging.^31^ However, the strong emission quenching because of the gold nanoparticles used upon the fluorescent dyes required a complicated design, which could pose difficulties for clinical setups: that was addressed by producing DSPE-PEG2000-based structures coloaded with both CAs. Another crucial factor in preparing bimodal nanostructures for nanomedicine applications is ensuring their biocompatibility. A common approach involves encapsulating the CAs within a SiO_2_ shell, which can then be further functionalized.^32,33^ However, this method adds complexity to the fabrication of the composite three-component platform and risks increasing the overall size of the nanostructures, potentially hindering their incorporation into cells.

Here we propose a synthetically controlled, facile, two-component platform alternative that consists of one material as the core luminescent CA with optical imaging potential (rare earth doped strontium aluminate core) and a second component that would serve as both the composite shell and the CA for the complementary CT technique, the cerium oxide. Specifically, for the nanoparticulate core, acting as the encapsulated optical imaging tag (or OI marker), we used a high-contrast material identified here as the nominal Eu­(II):SrAl_2_O_4_, synthesized by an optimized in situ reduction method that simultaneously leads to codoping with Dy^3+^ ions as auxiliary activators. This system exhibits a broad and high-brightness luminescent emission, effectively avoiding cellular autofluorescence effects which often pose microscopy challenges in cellular imaging assays.^18,21^ Here we also redeployed a ceria (CeO_2_) coating as the biocompatible shell since this offers a promising alternative to the well-established silica shells used in analogous nanoceramic core–shell particulates. Ceria is a biocompatible material known for its efficient dispersion in various biological fluids, including serum and saline solutions,^27^ and, as noted, can function as a feasible CT CA too due to its strong attenuation of incident X-rays.^30,34^ It is well established that cerium oxide (CeO_2_) nanoparticles can generate reactive oxygen species (ROS) due to their ability to undergo redox reactions, where they can easily switch between cerium­(III) and cerium­(IV) oxidation states. This redox activity can lead to the production of ROS, which are highly reactive and can cause cellular damage.^35^ However, when CeO_2_ is engineered into the shape of nanoplatelets, the generation of ROS can be mitigated. The nanoplatelet shape provides a larger surface area and unique morphology, which can enhance the antioxidant properties of CeO_2_.^35^ This allows the nanoplatelets to scavenge and neutralize ROS more effectively, thereby reducing the potential for ROS generation. Additionally, nanoplatelets can be coated with specific materials or functionalized to stabilize their surface, preventing the CeO_2_ nanoplatelets from participating in redox reactions that generate ROS.^35^ Surface coatings can also act as a protective barrier, preventing unwanted interactions with the environment. These factors combined can help in mitigating the generation of ROS when CeO_2_ is used in the form of nanoplatelets, making them more effective and safer for various applications.^35^ The resulting colloidal core–shell nanocomposites, labeled as CeO_2_@Eu,Dy:SrAlO, on the basis of the presence of a number of strontium aluminate phases as the matrix (Scheme 1), were synthesized via a simple and intuitive reverse micelle procedure, allowing precise control over the morphology and size of the heterostructures, with the aim to generate biocompatible materials that can be amenable to cellular incorporation. Their composition and morphology across nano- and microscales were thoroughly characterized using TEM and X-ray diffraction methods and imaged in thin films and cellular environments through multiphoton fluorescence lifetime imaging microscopy coupled with confocal fluorescence microscopy techniques.

**Scheme 1:**
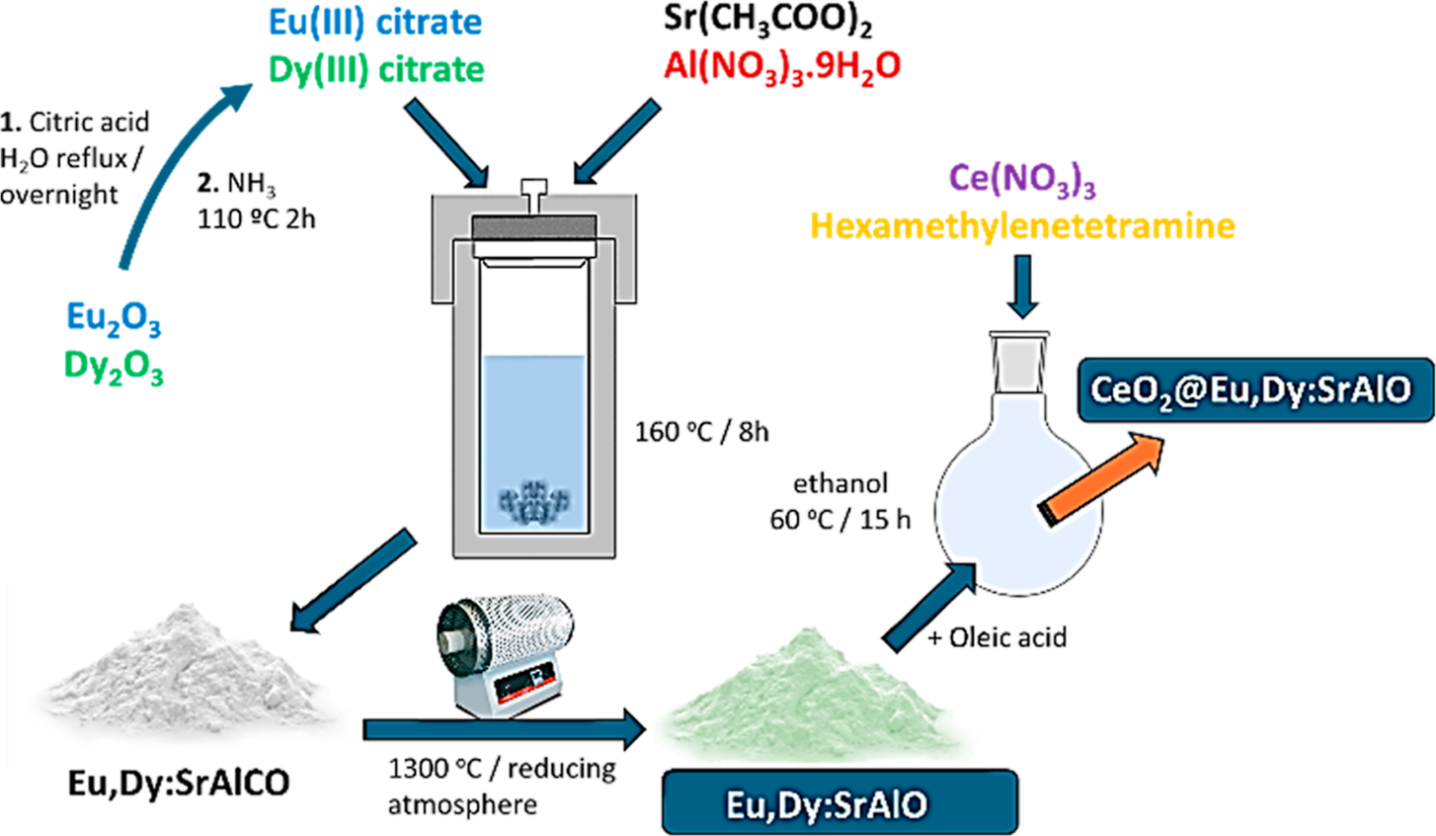
Schematic representation of the synthetic protocol applied hereby. The process was monitored via TEM and the evaluation of the dispersed nanoparticles scaffolds was performed using DLS particle size measurements at 25 °C in water (conc. 0.5 mg/mL). Note: A combination of phases consisting of SrAl_2_O_4_ (Monoclinic), Sr_1.88_Eu_0.12_Al_24_O_38_ (Hexagonal), Sr_3_Al_2_O_6_ (Cubic), and minor traces of Sr_10_Al_6_O_19_ (Monoclinic) occurred from the synthesis, therefore the material obtained is denoted hereby using the abbreviation Eu,Dy:SrAlO

## Results and Discussion

### Synthesis and Characterization of Eu,Dy:SrAlO Core Particles

The main design element redeployed here was the deliberate choice of a nominal strontium aluminate host lattice (denoted SrAlO) codoped with the in situ-generated divalent europium and trivalent dysprosium as the CA for optical imaging. Since strontium aluminates can be structured in a variety of stoichiometries, including SrAl_12_O_19_, Sr_4_Al_14_O_25_, Sr_3_Al_2_O_6_, SrAl_2_O_4_, and SrAl_4_O_7_, the first challenge to address was the evaluation of the crystalline phases formed after the in situ synthesis of the luminescent particles (performed as depicted in Scheme 1 and described in the Results and Discussion Section). Figures 1a and S4 (Supporting Information) show the X-ray diffractogram corresponding to the synthesized powder and the results show a mixture of phases consisting of SrAl_2_O_4_ (monoclinic), Sr_1.88_Eu_0.12_Al_24_O_38_ (hexagonal), Sr_3_Al_2_O_6_ (cubic), and minor traces of Sr_10_Al_6_O_19_ (monoclinic), all of them (which will be referred to hereinafter in the abbreviated form as Eu,Dy:SrAlO) luminescent materials^22,35^ when doped with rare earth metal ions. While there was a possibility that Eu­(III) traces remained following the reduction treatment, the method was in line with previously reported techniques and led to a majority of Eu­(II)/Dy­(III) dopants being encapsulated.^36^

**Figure 1:**
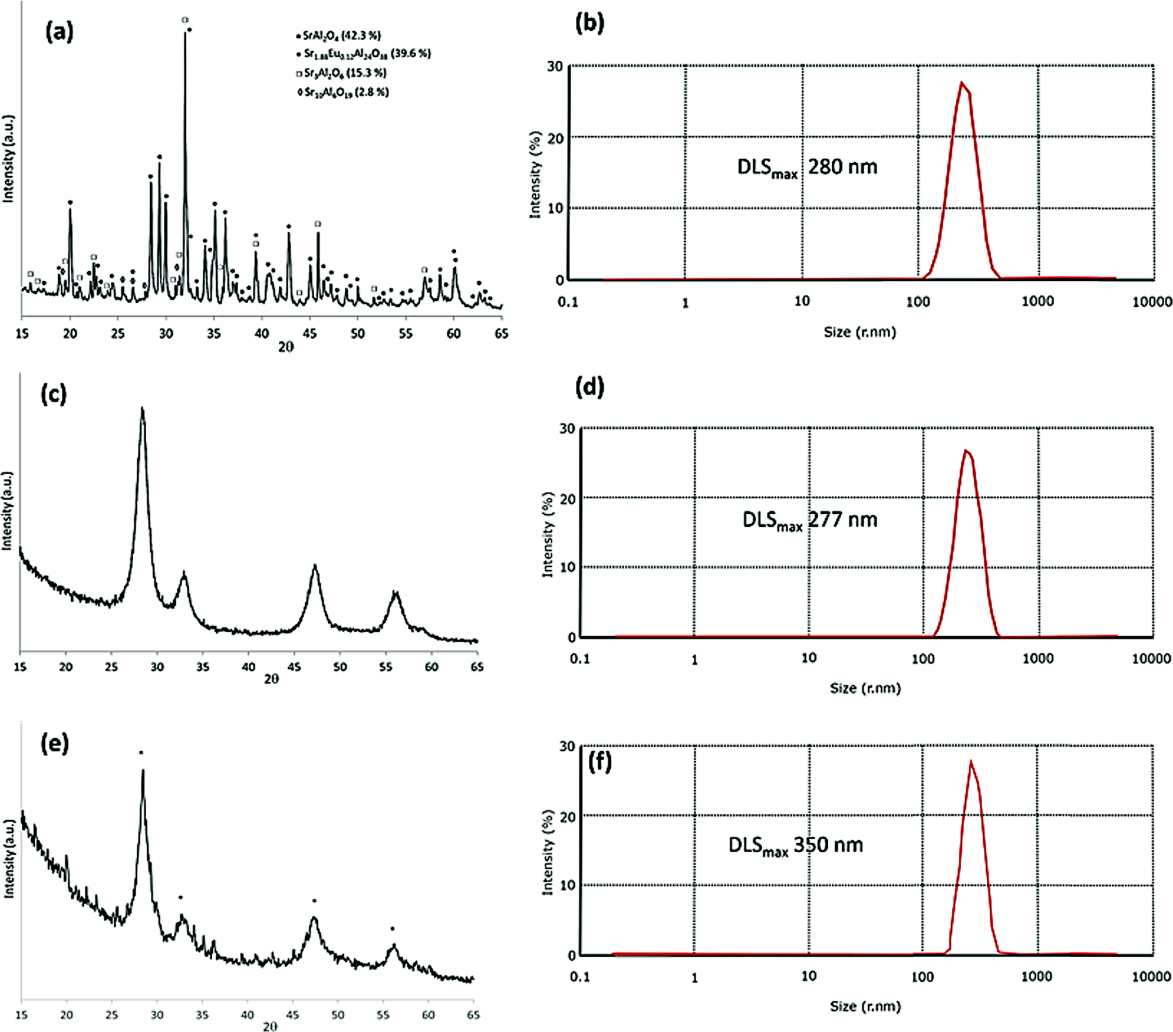
(a) Powder X-ray diffractogram and (b) DLS of the strontium aluminate codoped luminescent particles as obtained at 1300 °C. (c) Powder X-ray diffractogram and (d) DLS of the CeO_2_ shell. (e) Powder X-ray diffractogram and (f) DLS of the CeO_2_@Eu,Dy:SrAlO (* peaks corresponding to the CeO_2_ phase ICCC: 00-034-0394).

The hybrid and inhomogeneous nature of these NPs with expected spinel-type formula SrAl_2_O_4_ was observed and characterized in depth. The results of the X-ray analysis in solid state were complemented by the data obtained by the DLS technique for the range of nanoparticulate materials of interest. Traces shown in Figure 1b,d,f were obtained for the core Eu,Dy:SrAlO core, the ceria shell, and the ceria−coated coreshell hierarchical nanoparticles assembles, denoted CeO_2_@Eu,Dy: SrAlO each dispersed in 1 mg/L in deionized H_2_O.^37^ These DLS all showed that these materials present a narrow distribution of the resulted aqueous aggregates in the ca. 250 nm ranges, with a slightly large size for the strontium aluminate cores centered at 280 nm. However, the DLS values are highly dependent on the type of solvent used for the measurement and could correspond to either individual particles or agglomerates. To further complement these findings, transmission electron microscopy was used and representative images of the nanoparticles of interests, imaged on the nano- and microscale, are presented in Figures 2 and S1. Interestingly, TEM experiments also show that these particles have characteristic shapes corresponding to the different phases, assigned by X-ray diffraction and given in the Supporting Information (Figure S4) with a predominant platelet-like morphology and appropriate dimensions of less than 150 nm in length and ca. 30 nm in width, as evaluated by high-resolution transmission electron microscopy (HRTEM) (Figure S1, Supporting Information).

**Figure 2:**
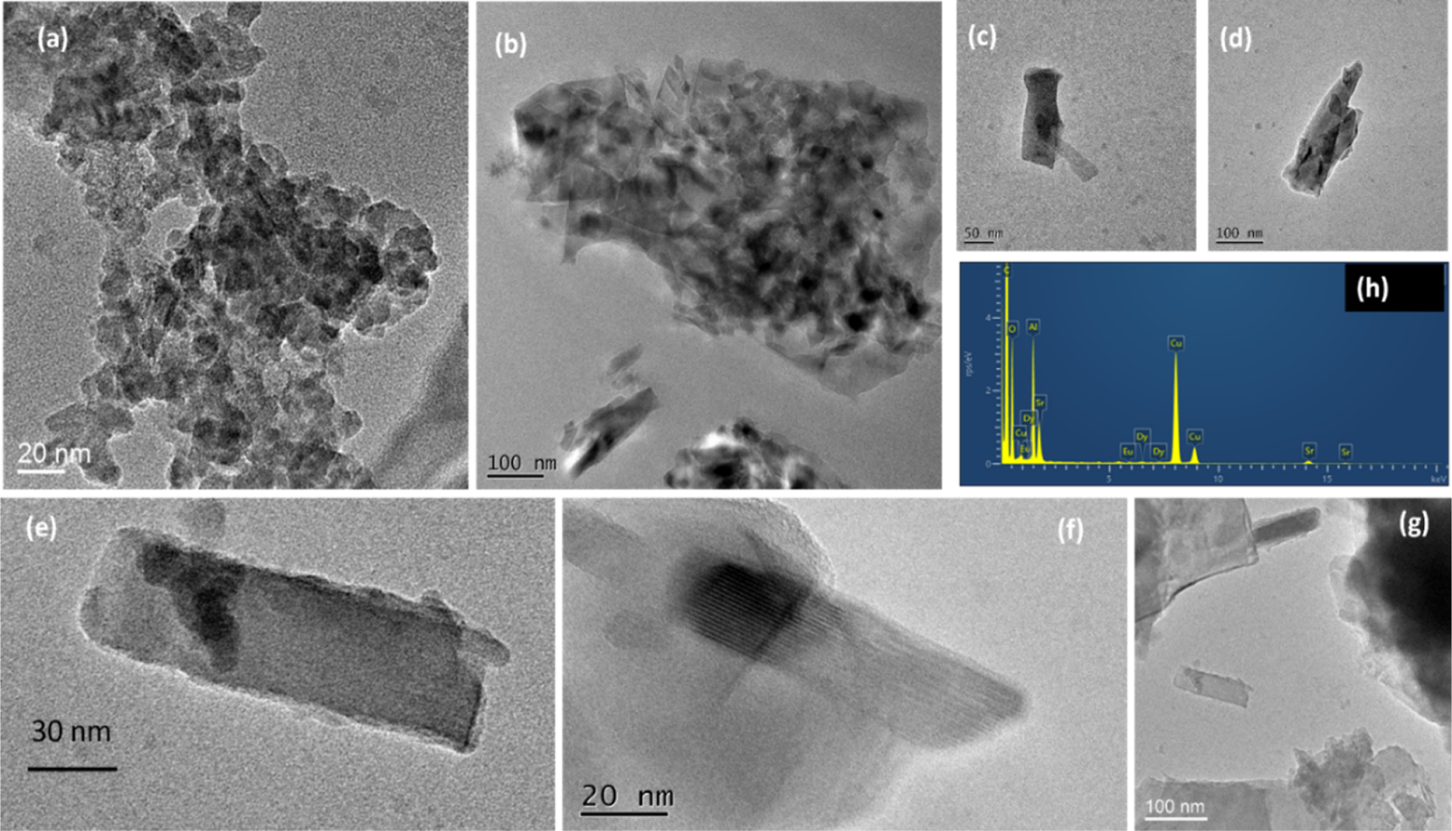
Representative TEM micrographs of the core luminescent particles involved in this study, (a–g) imaged at different magnifications (Eu,Dy:SrAlO) and (h) corresponding energy-dispersive X-ray (EDX) analysis of the strontium aluminate codoped luminescent particles as obtained at 1300 °C.

In line with the findings from X-ray data, the particles corresponding to the different phases of strontium aluminate are clearly smaller in size than the value detected by DLS, although they were also observed to form larger agglomerates. As stated above, their sizes are well within the expected range for nanoparticles used for bioimaging in water, ca. 250 nm.

The optical properties of the as-synthesized powder denoted Eu,Dy:SrAlO were first analyzed by UV/Visible spectroscopy in thin films and in the dispersed phase (1 mg/mL in H_2_O). The absorption spectrum in Figure 3a shows that the doped nanoparticles absorb effectively in the UV region. These emission properties were further evaluated by fluorescence spectroscopy in thin films and dispersions after exciting the sample with a wavelength of 375 nm. The two-photon excitation at 800 nm rather than single-photon UV excitation was used as this offered a significant advantage for deeper tissue penetration instead and was utilized hereby to shed light into the cellular penetration prospects of these platelet like materials. The corresponding emission spectrum is plotted in Figure 3b and shows an emission peak centered at 505 nm within the visible range of interest. Additional spectroscopic data are given in Supporting Information (Figures S5). The current literature associates this emission maximum with the transition from the 4f^6^ 5d^1^ to the 4f^7^ states of Eu^2+^, slightly shifted to longer wavelength due to the presence of Dy^3+^.^38,39^ Specifically, both the emission and excitation spectra of the divalent europium ion usually consist of bands such as those observed in our samples due to the transitions between the 4f^7^ (^8^S_7/2_) ground state and the crystal field components of the 4f^6^ 5d^1^ excited state configuration. When a sample containing the Eu,Dy:SrAlO nanoparticulate cores was excited using either single photon excitation (375 nm) in thin film or two photon excitation (800 nm) in a 1 g/L dispersed phase in water, the fluorescence emission spectra recorded (Figures 3d and S6) showed a further red emission band characteristic of the narrow Eu­(III) codopant traces, which was observed at ca. 705 nm and particularly enhanced in the presence of H_2_O and under 2 Photon excitation. This feature is entirely expected from traces of unreduced Eu­(III) present and is in line with the reported investigations into Eu­(II)/Eu­(III) in codoped systems in both the presence and absence of Dy­(III), in solid phase, as well as in aqueous environment.^36,40–44^

**Figure 3:**
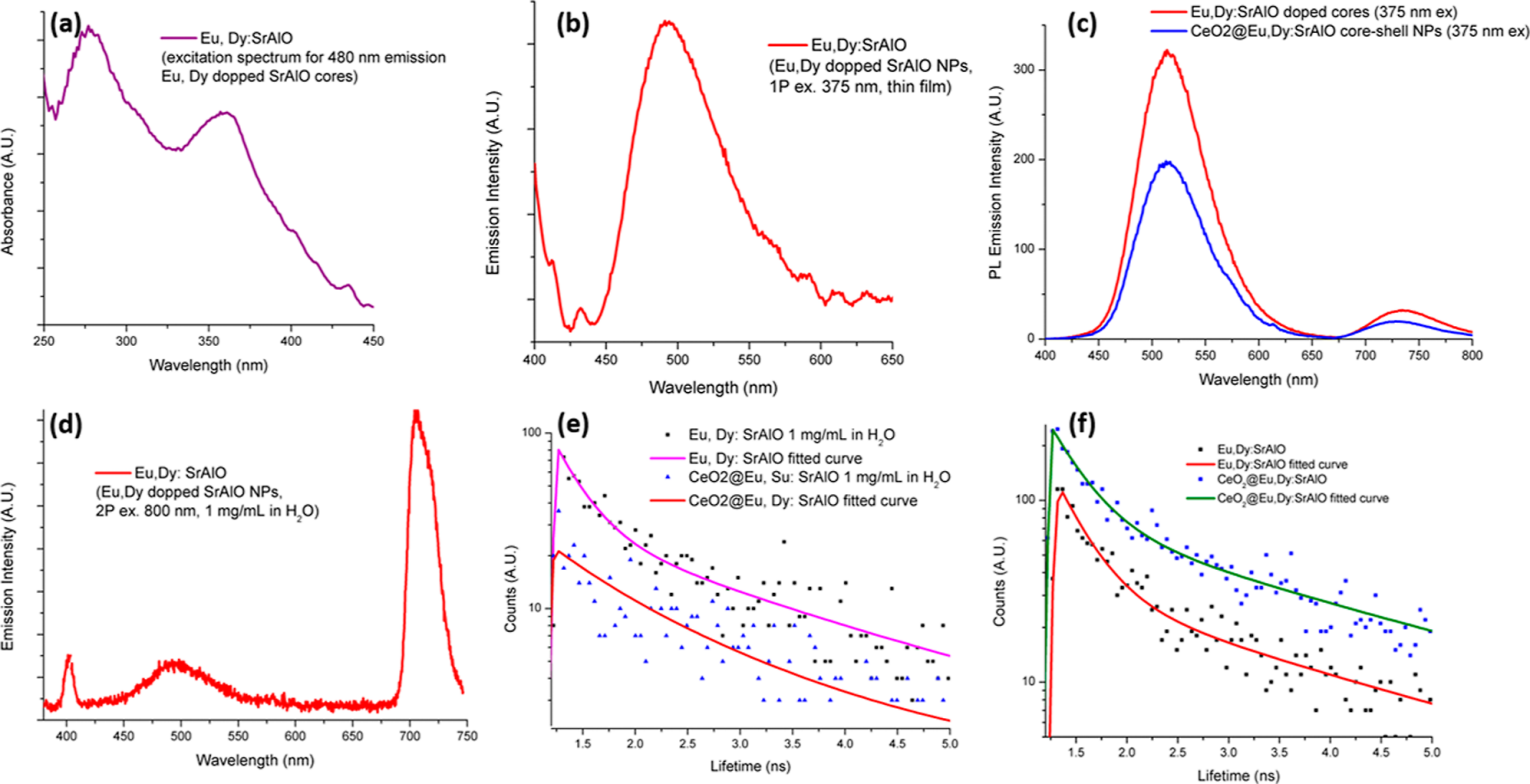
Spectroscopic characterization: solid state UV–vis spectrum (a), fluorescence emission spectrum (λ_exc_ = 360 nm) of the luminescent particles (Eu,Dy:SrAlO) in solid state (b), fluorescence emission spectra (λ_exc_ = 360 nm) of the luminescent particles (Eu,Dy:SrAlO) and core–shell composite (CeO_2_@Eu,Dy:SrAlO) in H_2_O dispersed phase (1 mg/mL) (c), two photon fluorescence emission spectrum (λexc = 800 nm) of the luminescent particles (Eu,Dy:SrAlO) suspension in water at 1 mg/mL concentration (d), representative fluorescence lifetime data in dispersed phase (e) and in thin films (in representative pixel-by-pixel spot) for the nanomaterials investigated further details are given in Table 1 and Supporting Information (f).

### Synthesis and Characterization of the Core–Shell Nanocomposites (CeO_2_@Eu,Dy:SrAlO)

With the luminescent cores Eu,Dy:SrAlO in hand, the synthetic procedure depicted in Figure 1 was followed by the CeO_2_ shell coating protocol. However, prior to this step, it was necessary to establish which specific CeO_2_ species emerged from the reverse micelle protocol used for the encapsulation. In doing so, a control experiment involving only the final coating step was performed using ceria alone and without incorporating the core nanoparticles (see the Experimental Section). Here a 0.1 M mixture of cerium­(III) nitrate and hexamethylenetetramine (HMTA) in ethanol (96%) was subjected to the 60 °C defined in the protocol and, after 2 h, a yellowish white precipitate was produced, which was removed by centrifugation and dried for a further 24 h at 60 °C.

The as-obtained CeO_2_ powder was first analyzed by a range of characterization techniques, as follows. The crystallinity and phase composition was studied by XRD and results (with X-ray data and simulation depicted in Figure 1c and Supporting Information Figure S8) indicated the presence of pure CeO_2_ phase crystallizing in the cubic system. This is attributed to the presence of HMTA, which acts as a complexing agent and stabilizes the 4+ oxidation state of cerium. Subsequently the light absorption properties of the ceria nanoparticles used for coating were estimated. The corresponding UV/visible absorption spectrum is shown in Figure 4 and evidences a peak around 200 nm and a shoulder at 300 nm. Next, the average size of the powder CeO_2_ particles was evaluated by DLS as 0.5 mg/mL water dispersions Figure 1. Consistent with the literature which compares data in dispersed phase with those results on the atomic scale from TEM microscopy^45^ and as discussed above, DLS results are affected by the solvent used to perform the measurement, so two different experiments were conducted, one with the particles suspended in ethanol and the other after dispersing them in oleic acid. Interestingly the ethanol suspension was unable to prevent agglomeration of the particles while the DLS analyses in oleic acid showed the maximum centered at 277 nm with an approximated width of 50 nm, which was deemed satisfactory for further experiments and the full reverse micelle protocol involving the cerium oxide to coat the luminescent nanoparticles was performed.

**Figure 4:**
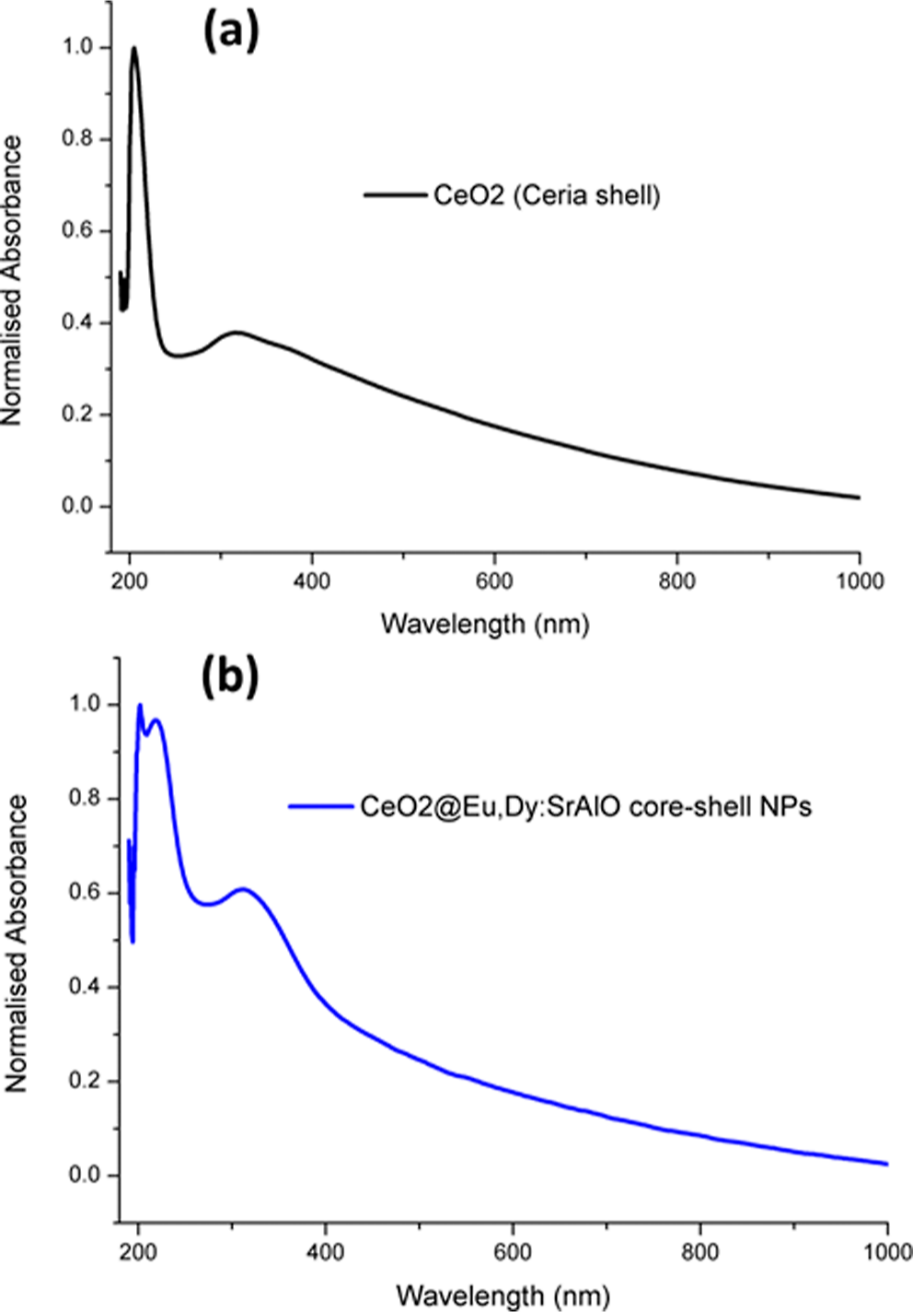
Solid state UV/visible absorption spectrum of the CeO_2_ shell (a) and core–shell composite (b).

The core–shell target structures denoted CeO_2_@Eu,Dy:SrALO were prepared according to the reverse micelle protocol detailed in the Experimental Section. Similar to the case of the individual components, the crystal phase composition of the obtained composites was first determined by X-ray diffraction (Figures 1e and S13 Supporting Information).

TEM and HRTEM demonstrate that the desired NPs have a core–shell structure with a dark contrast metal core and a somewhat lighter contrast CeO_2_ shell. The successful incorporation of Eu,Dy:SrAlO into CeO_2_ shell NPs was further confirmed by optical spectroscopy and the simultaneous presence of Eu, DY, Sr, Al, and Ce peaks (Figures 5 and S10–S15) in TEM-coupled EDX spectra and extensive XRD measurements (Figures 5 and S10, S11 and S13, Supporting Information). Figure 5 shows a typical TEM micrograph of core–shell nanoparticle, which we assign to the CeO_2_@Eu,Dy:SrAlO. Generally, the TEM of the NPs imaged indicate dimensions at ca. 150 nm. DLS showed that dispersion in aqueous solvents had size distribution centered at 350 nm which is expected when compared to the case of CeO_2_ alone or Eu,Dy:SrAlO (Figures 1f, 5). Although the DLS suggests a degree of aggregation in aqueous solutions, it is known that this technique yields higher diameters than those observed by TEM.^46,47^ The corresponding XRD pattern is plotted in Figure 1e, showing that most of the detected maxima belong to the CeO_2_ phase of the shells. Diffraction peaks attributable to the strontium aluminate phases of the luminescent cores are also visible, although, because of the coating treatment, their phase distribution (percentage) has been slightly modified. The size of the nanoparticles was again estimated by DLS in aqueous environments and imaged by TEM. DLS measurements indicated an average particle size for the core–shell assembly centered at 300 nm, which remained suitable for cellular imaging. TEM characterization was in line with these estimated values and further reveals that the core–shell composites contain several of the luminescent nanoparticles inside, with an effective CeO_2_ coating of up to 10 nm thick, and the composition was confirmed by EDX.

**Figure 5:**
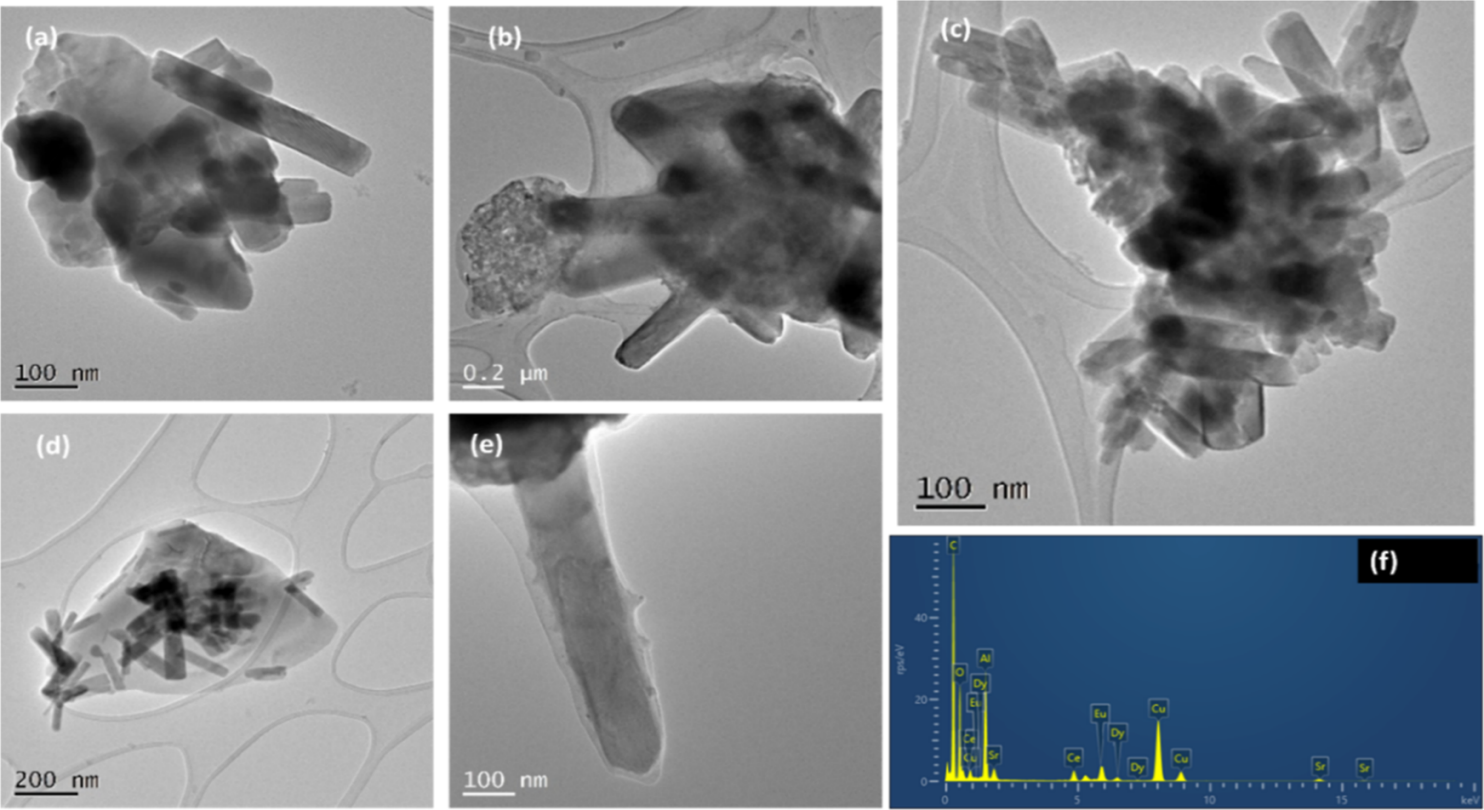
Representative TEM micrographs showing the platelet-like nature of particles investigated, on a range of magnifications (a–e) and EDX analysis of the core–shell composite CeO_2_@Eu,Dy:SrAlO. (f). Alternative images are given in the Supporting Information.

We found through extensive coating experiments monitored by TEM and UV–vis spectroscopy that this thin shell layer of CeO_2_ was ideal, as it did not significantly diminish the optical response of the luminescent cores and thus can bode well for the usefulness of these composite structures in optical imaging. Moreover, the fact that such a thin coating gives rise to the intense CeO_2_ signals observed by X-ray diffractions is exciting: this is due to the increased refraction of X-rays by the cerium atoms and represents an indirect evidence that this coating and the synthesized core–shell structures CeO_2_@Eu,Dy:SrAlO also have potential for CT applications.

Spectroscopic Investigations in aqueous dispersions and in thin films. The composites were then characterized by UV/vis and fluorescence spectroscopy in the thin film deposited from 1 mg/mL dispersions in H_2_O.

As depicted in Figures 3 and 4, absorption bands of both the cerium shell and luminescent cores are observed. Absorption is observed within the entire spectral range investigated (up to 900 nm) due to the inhomogeneity of the dopants present in the hybrids. However, this should not be a major drawback toward their explorations in biological milieu since in the final core–shell structures, the maximum thickness of the outer cerium shell will be strictly controlled to a few nanometers, as evidenced by extensive HRTEM measurements. Complementary fluorescence spectroscopy measurements confirmed that the luminescent properties of the cores were barely screened by the biocompatible CeO_2_ shell. Moreover, the absorption maximum corresponding to the cores is more defined than that in the bare luminescent particles and even shows a higher absorbance. This is a well-known effect observed in core–shell engineering of luminescent platforms: the coating with the thin ceria shell not only retains the optical integrity of the luminescent cores but further reduces emission losses caused by surface quenching effects.^48^ Fischer et al. and Kim et al., respectively, demonstrate that coating with an inert shell, such as ceria, enhances luminescence by suppressing surface quenching effects. It supports the claim that the ceria shell reduces emission losses and maintains the optical properties of the cores.^48^ The quantum yield of both Eu,Dy:SrAlO and the corresponding CeO_2_-coated materials was also calculated by measuring directly and indirectly in integrating sphere to calculate the yield, obtaining values of 11.22% and 15.86% respectively, which compare well with the previously reported values for related nanophosphors.^35,48^ Accordingly, we can expect the prepared core–shell structures to have potential as fluorescent imaging agents for in vitro studies of cells and tissues. This hypothesis was confirmed by two-photon laser scanning confocal microscopy measurements, which were first performed on both the uncoated core nanoparticles and the core–shell nanostructures. We redeployed here two-photon excitation techniques because these provide high spatial resolution and, when combined with FLIM, often termed multiphoton FLIM (MP FLIM), enable the exploration of photophysical properties of molecules and nanomaterials in the emission lifetime domain. This approach offers complementary insights beyond steady-state intensity-based confocal fluorescence imaging, particularly in thin films and cellular environments. FLIM excels at probing molecular or nanoparticle environments, as excited-state lifetimes are highly sensitive to environmental factors such as pH, viscosity, and organelle trapping, making them effective analytical probes. Standard fluorescent probes with short emission lifetimes (picoseconds to nanoseconds) are limited by their overlap with background autofluorescence and minimal lifetime variations required for FLIM analysis. Phosphorescent nanoparticles, by contrast, possess long-lived triplet excited states, offering significant advantages. These include bypassing autofluorescence, enabling detection of larger lifetime variations and providing tunable absorption and emission properties through size, morphology, and codoping characteristics (e.g., with Eu^2+^, Eu^3+^ and Dy^3+^ ions, single or in combinations, yet the method is easily generalized and extendible to a range of relevant rarer earth elements). Additionally, phosphorescence lifetime imaging microscopy (PLIM) measures longer emission time scales (>100 ns), complementing FLIM. In this investigation, 800 nm two-photon excitation was applied in both FLIM and PLIM modalities to Eu,Dy:SrAlO materials. Together, PLIM–FLIM probes, coupled with tissue-friendly near-IR excitation using 2-photons, might allow simultaneous imaging across multiple luminescence modalities with submicron spatial resolution. This approach enabled verification of the Eu^3+^ codopant’s detectability alongside the Eu^2+^/Dy^3+^ diade, utilizing emission lifetimes differing by orders of magnitude: nanoseconds for Eu^2+^/Dy^3+^ and micro- to milliseconds for phosphorescent Eu^2+^/Eu^3+^/Dy^3+^ species. Such capabilities support multimodal imaging with distinct luminescence outputs across spectral regions.

For Eu,Dy:SrAlO and corresponding CeO_2_@ Eu,Dy:SrAlO core–shell nanoparticles, MP FLIM was performed in the thin film as well as when incubated with living cells, using laser scanning microscopy (LSM) as the imaging modalities. The excitation-detection technique used the LSM systems was in correlated confocal (CLSM) with multiphoton (MP LSM) systems, and these microscopic techniques provided the necessary 2P FLIM data for the systems analyzed, where the fluorescence lifetimes were measured in the time domain. We previously applied the correlated FLIM measurement methods together with time-correlated single-photon counting (TCSPC) where a photon-overtime histogram was measured, I each case. The intensity *I*(*t*) was calculated by convolving the systems instrument response function and the weighted sum of several fluorescence decays, typically modeled via simple first-order exponential functions, as shown in eq 1. Here, FLIM measured the time delay between excitation and emission photons using TCSPC with time-gated detection ,which was previously used in our similar experimental setups and sample characteristics for tracing nanoparticulate materials with a range of dimensions and morphologies in cells from silica coated nanoparticles^8^ to graphene oxide nanohybrids^49^ in living cells.

To extract a meaningful overview of the nanoparticulate materials studied and shed light on the behavior of these materials within a range of environments (e.g., dispersed aqueous phase, thin film drop-casted and dried on borosilicate glass and in the presence of living healthy and cancerous cells, as imaged hereby), the lifetimes (τ_*i*_ = τ_1_, τ_2_, etc.) and corresponding % abundances (*a*_*i*_ = *a*_1_, *a*_2_ etc.) were first extracted on pixel-by-pixel bases from the field of view, where the lifetimes τ_*i*_ represent the different types of chromophores and abundances highlight their local concentration. Data corresponding to these lifetimes and abundances from the measured decay traces are fitted using standard curve-fitting methods: the photon histograms are generated by applying eq 1 where the fitting curve method is highly sensitive to the signal-to-noise ratio, which is known to increase with higher photon counts. Lifetime calculations were obtained using SPCImage analysis software (Becker and Hickl, Germany) or Edinburgh Instruments F900 TCSPC analysis software. In these experiments, fluorescence decay curves were well fitted to a multiexponential function where *a*_*i*_ and τ_*i*_ are the amplitudes and decay times of the exponential components of the fluorescence decay. The percentage of components τ_1_ (e.g., major) and τ_2_ (e.g., minor) in a decay can be evaluated from the respective amplitudes *a*_1_ and *a*_2_ calculated with standard programmes, for example, the SPCImage software analysis software (Becker and Hickl, Germany), which models the data for each individual pixel, where *F* is fluorescence, *a*_0_ is background, and *t* is time, in eq 2, and the fwhm, calculated from the lifetime distribution curve within the focal area, is typically used to assess the error (eq 3).^50^

1 $$I\left(t\right)=IRF^{*}\sum_{i}^{n}a_{i}e^{-t/\tau_{i}}$$

2 $$I\left(t\right)=\sum_{i}^{M}a_{i}\exp\left(\frac{-t}{\tau_{1}}\right)$$

3 $$F\left(t\right)=a_{0}+a_{1}e^{-t/\tau_{1}}+a_{2}e^{-t/\tau_{2}}$$

Figures 6 and S19–S24, S26–S30 (Supporting Information) show confocal images, the fluorescence lifetime and intensity maps distribution, and the associated distribution profile of a water suspension of both the strontium aluminate bare nanoparticles and the core–shell composites. Fluorescence decay curves required fitting to two components (Figure S16, Supporting Information), indicating a complex molecular environment. For the bare uncoated cores, a predominant fluorescent response is obtained with an average lifetime <1200 ps, Figure 6a–c. In a representative spot, the corresponding decay curves are shown in Figure S22 (Supporting Information), which report a ratio *a*_1_ = 68% and a lifetime τ_1_ = 277 ps for the first component of the fitting and a ratio *a*_2_ = 32% with a lifetime τ_2_ = 1976 ps for the second component (additional values are given in Table 1 and Supporting Information, Figures S16–S48). In addition, the lifetime map images reveal the presence of minority regions of intense blue coloring (lifetimes ca. 3000 ps) with decay curve suggestive of the possibility of additional longer-lived phosphorescent emission behavior, which was then probed by PLIM in thin films (see Supporting Information, Figures S25 and S31). It has been already shown that under certain conditions similarly doped strontium aluminate nanoparticles can display a persistent luminescent response assignable to the codoping of Eu^2+^ and Dy^3+^.^34^ Here the 2P FLIM lifetime mapping depicted in Figure 6 indicated a slightly shifted yet broad fluorescence lifetime distribution of Eu,Dy:SrAlO, with the distribution trace being skewed toward the yellow-green area of the “rainbow” representation used in lifetime map (<1800 ps). In a representative spot, the decay curves and fittings show a slower decay than that of the bare cores, with fitting values now giving generally values of τ_1_ = 555 ps (*a*_1_ = 35%) for the first component and τ_2_ = 1482 ps (*a*_2_ = 65%) for the second component (additional values are given in Table 1 and Supporting Information). Interestingly, although the proportions of the components hardly change, the second component clearly corresponds to longer lifetime for the composites.^51^ These observations support what was observed with the UV/vis measurements (Figure 6) in thin films; the ceria coating minimizes the surface defects of the core nanoparticles and, also, by encapsulating several particles inside (agglomerates), results in a more efficient luminescent response with a longer lifetime. We reported previously on our observations that variations in nanoparticulates size lead to luminescence inhomogeneity and that a decrease in the luminescent properties of the system in the solid state seems to occur upon reduction in particulate size.^20^ In this work, the bottom-up strategy carried out to synthesize the strontium aluminate cores led to particles with sizes in the range of 50–150 nm (Figures 1 and 2), which, although suitable for cellular uptake, appeared to show a reduction in their phosphorescence. The identified blue regions can thus be ascribed to the formation of larger agglomerates (or aggregates) of nanoparticles, and we image the phosphorescence of these materials deposited in thin films by PLIM. The imaging configuration of the FLIM setup incorporated femtosecond tunable lasers and OPO with a range from 400 to 1600 nm. This configuration, with GaAsP hybrid detectors (900 nm sensitivity), is also advantageous to carry out lifetime studies since the inorganic species used show a wide range of excited state life times, in >100 ns range and even spanning lifetime of 1 μs to 1000 ms. By contrast, the lifetimes of the standard organic dyes (e.g., NucBlue Live ReadyProbes Reagent or Hoechst 33342) ranged from 600 to 1000 ps under this setup under 800 nm 2P excitation and cellular autofluorescence as expected and showed characteristic FLIM distributions in ca. 1.5–2.5 ns. A preliminary FLIM–PLIM experimental setup coupled to TCSPC detection on a pixel-by-pixel basis was also explored hereby as it provided excellent spatial and temporal resolution, which is crucial for this application. PLIM is a similar technique to the more established FLIM method, the key difference being, it measures emission on a longer time scale (>100 ns) and is hence compatible with determination of triplet emissive states. FLIM–PLIM–TCSPC can potentially achieve imaging over decades of time-scales to provide highly useful insights into the aggregated nature and environment of these materials that is not accessible so far in any other microscopy method.

**Figure 6:**
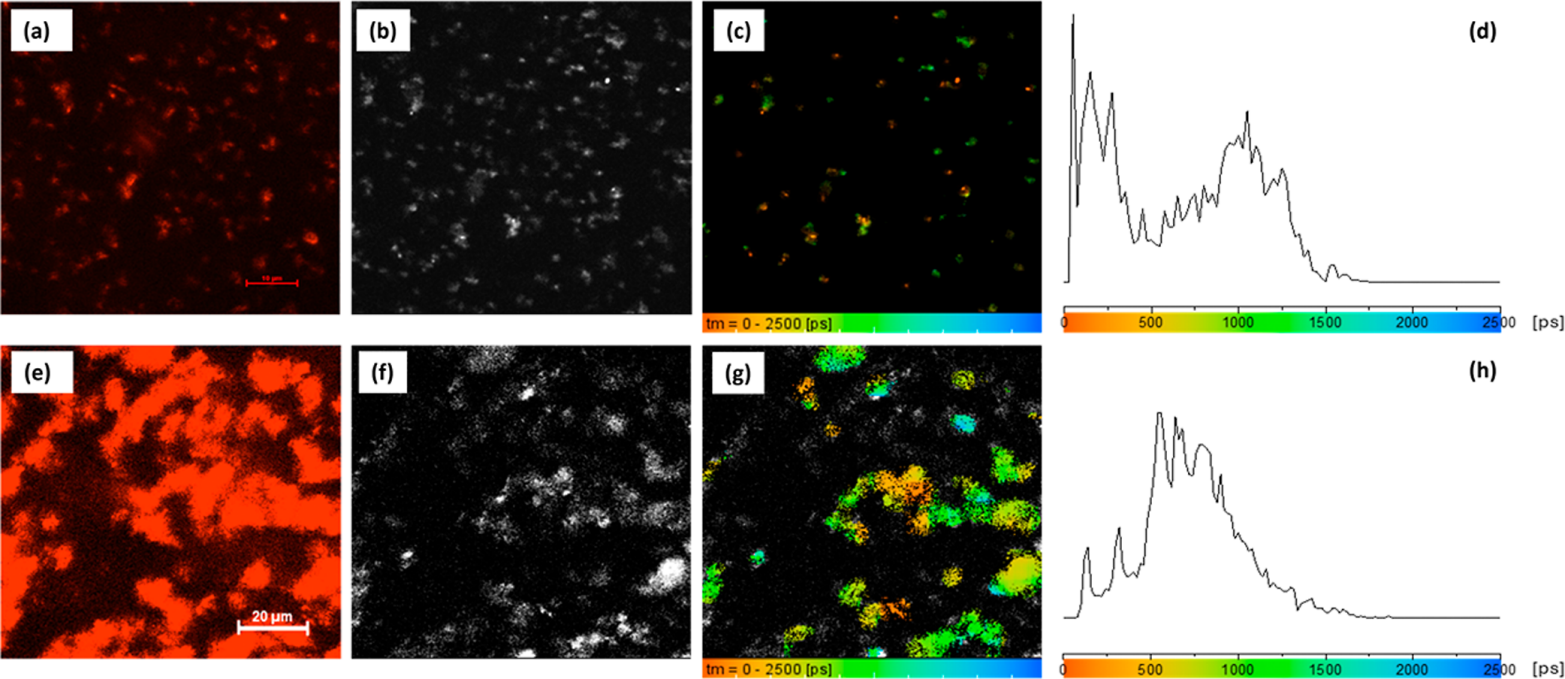
Correlated imaging: single-photon laser confocal fluorescence (λ_ex_ = 400 nm and λ_em_ = 570–750 nm) for Eu,Dy:SrAlO (a) and CeO_2_@Eu,Dy:SrAlO (e). Correlated two-photon fluorescence microscopy measurements (scale-bar 20 μm, laser power: 2.0 mW at 800 nm 2P excitation) of particle films showing: fluorescence intensity (b,f, respectively), fluorescence lifetime maps (c,g) and associated profile distribution for (a–d) uncoated core particles Eu,Dy:SrAlO and (e–h) ceria-coated composites, CeO_2_@Eu,Dy:SrAlO. Rainbow colors mapping provides a direct correlation between the lifetime in field of view (c,g) and the fluorescence lifetime distribution histograms (d,h).

**Table 1:** Selection of Experimental Fluorescence Lifetime Decays and Corresponding Fitted Parameters for the Two-Photon Excitation TCSPC Measurements at 800 nm Excitation, in Thin Films and in Living Cells^a^ ^,^ ^b^

2P FLIM in thin film and TCSPC in 3 randomly selected spots (800 nm, laser power 2 mW)							2P FLIM and TCSPC in 3 selected spots (800 nm, laser power 2 mW, 37 °C, 15 min in CHO, fwhm peak ± error, ns)						
spot		τ_1_ (ns)	*a*_1_ (%)	τ_2_ (ns)	*a*_2_ (%)	χ^2^	spot		τ_1_ (ns)	*a*_1_ (%)	τ_2_ (ns)	*a*_2_ (%)	χ^2^
Eu,Dy:SrAlO fwhm: ±0.12 ns	1	0.28	68	1.98	32	1.24	Eu,Dy:SrAlO fwhm: ±0.22 ns	1	0.33	76	2.51	24	0.79
	2	0.20	58	1.35	42	0.63		2	0.30	76	2.34	24	1.17
	3	0.27	76	2.45	24	1.15		3	0.31	67	2.17	33	1.04
CeO2@Eu,Dy:SrAlO fwhm: ±0.23 ns	1	0.24	75	1.82	25	1.08	CeO_2_@Eu,Dy:SrAlO fwhm: ±0.15 ns	1	0.33	71	2.50	29	1.01
	2	0.19	80	2.16	20	1.23		2	0.30	69.5	2.14	30.5	1.06
	3	0.17	52	1.17	48	1.09		3	0.22	68	2.06	32	1.17

a Corresponding 2P FLIM data associated to micrographs of thin films and living (healthy) Chinese hamster ovary (CHO) at 37 °C are depicted in Figures 6–8. The full width at half maximum, calculated from the lifetime distribution curve within the focal area, was used to estimate the range of the 2P lifetime distribution and in each case, this was found to be very broad, as illustrative of the lifetime inhomogeneity in the nanoparticles analyzed across various environments.

b Note: the corresponding PLIM data (400 nm excitation) are given in the Supporting Information for thin films, whereas associated experiments in living cells did not give sufficient counts to conclusively derive PLIM fitted parameters. 2P FLIM in (cancerous) PC-3 cells and control experiments probing for cellular autofluorescence are given in the Supporting Information alongside corresponding parameters for the imaging with the known organic dye nucleus blue dye (which was tested by 2P FLIM in CHO, τ_1_ 1.12 (ns), *a*_1_ 50% and τ_2_ 3.01 ns, *a*_2_ 50%, fwhm ±0.22 ns). Fitted parameters for random spots in living control CHO cells (37 °C, 1% DMSO) are τ 1.1 (ns), *a*_1_ 100% fwhh = ± 0.13 ns and for living PC-3 cells (37 °C, 1% DMSO) are τ 0.69 (ns), *a*_1_ 100% fwhm ±0.23 ns.

PLIM is applicable to the Eu­(II)/Eu­(III) codoped with Dy­(III) nanoparticles in this family, with >100 ns lifetimes, i.e., significantly longer than that of the organic dyes, 350–650 ps, and similar to previously tested Ir/Eu­(III) and Pt­(II) complexes where a similar setup was previously tested (with lifetime >50 ns).^52^ In the case of Eu,Dy:SrAlO and the corresponding core–shell composites CeO_2_@Eu,Dy:SrAlO, the 1P emission intensity in thin films could be correlated with the PLIM/TCSPC data and phosphorescence emission under excitation of 400 nm. Figure 7a,e indicates fluorescent response in both materials (arbitrary colored, ex 400 nm), (b,f) represent phosphorescence intensity images, and the (c,g) micrographs represent the phosphorescence emission in thin films with corresponding “rainbow colored” mapping and lifetime distributions (Figure 7c,d for Eu,Dy:SrAlO and Figure 7g,h composites CeO_2_@Eu,Dy:SrAlO). These PLIM images (Figures 7 and S25 and S31, Supporting Information) are consistent with previous findings in the 2P FLIM mode (Figure 6) for both materials were analyzed in thin films, deposited from aqueous solutions (1 mg/mL in H_2_O). However, while detectable in thin films, the phosphorescence emission signals did not seem to be sufficiently strong to be detectable in cells, unlike the corresponding 2P FLIM signals from samples of the same batch (Figure 6, two-photon excitation 800 nm).

**Figure 7:**
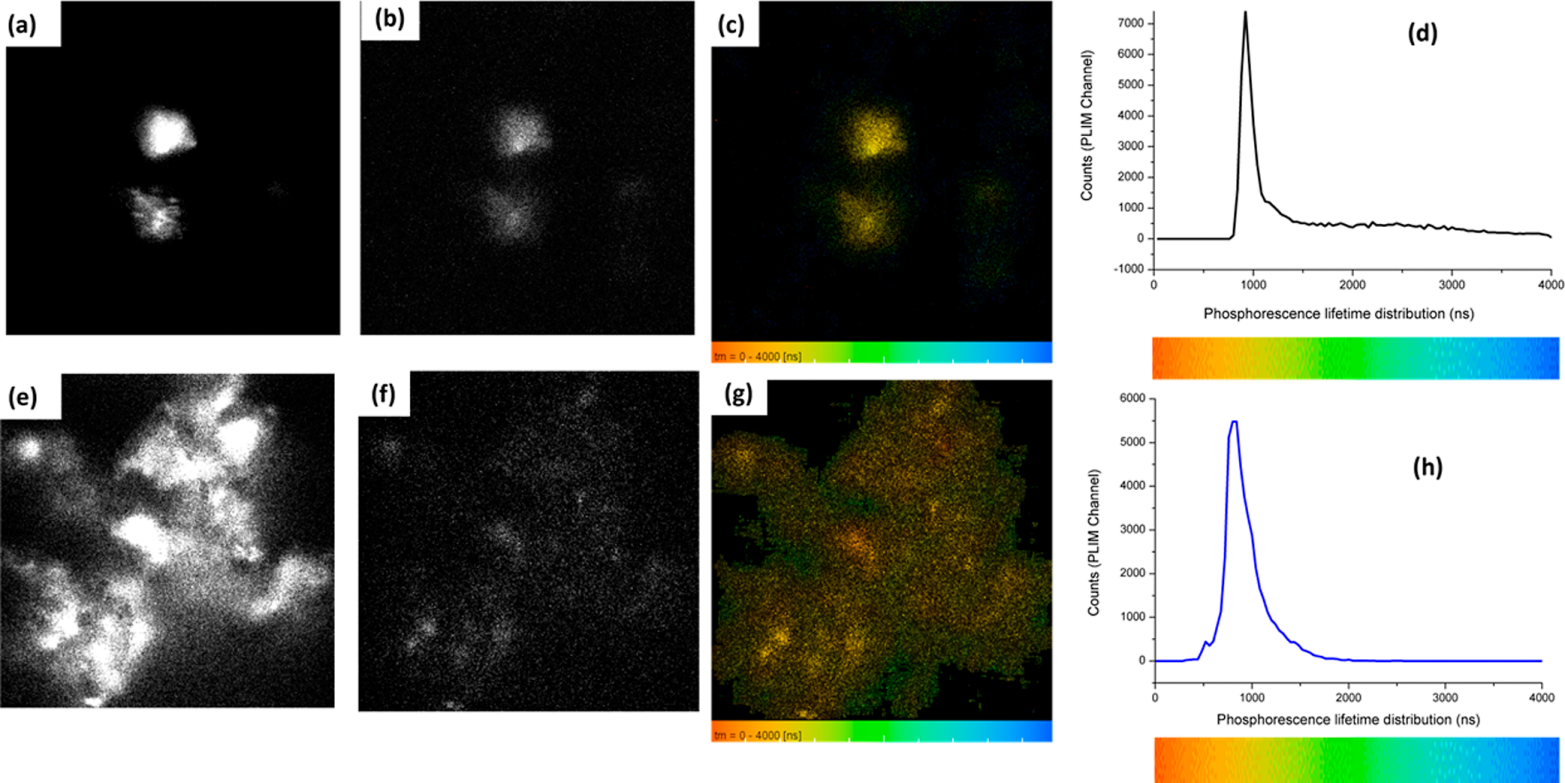
Correlated PLIM microscopy measurements of particles in thin films (400 nm excitation). Fluorescence emission intensity channel (λ_ex_ = 400 nm and λ_em_ = 570–750 nm) for Eu,Dy:SrAlO (a) and CeO_2_@Eu,Dy:SrAlO (e). Correlated PLIM measurements of particle films showing: phosphorescence emission intensity (b,f, respectively), phosphorescence lifetime maps (c,g), and associated profile distribution for (a–d) uncoated core particles Eu,Dy:SrAlO and (e–h) core–shell composites CeO2@Eu,Dy:SrAlO. Rainbow colors mapping provides a direct correlation between the phosphorescence lifetime in field of view (c,g) and the phosphorescence lifetime distribution histograms (d,h).

### Cellular Uptake Investigated Using MP FLIM

Two-photon laser scanning confocal microscopy analyses were next conducted on mammalian cells following the uptake of the particles. First, CHO cells were grown (to around 70% confluency) according to standard protocols on glass bottom dishes (see the experimental section for cell culture and plating details). The fluorescence lifetime map distribution of the compound in CHO cells and the associated distribution profile are shown in Figures 8 and S32–S45, Supporting Information. Figure 8a,e,I, respectively, shows the bright field confocal images for the bare nanoparticles and the core–shell composites after a 15 min incubation period. As observed in the corresponding lifetime maps, Figure 8, both systems were readily taken up by the cells and distributed throughout the cytoplasm, producing a stable fluorescent environment within the cells. The internalization is visibly more effective when the particles are coated with a cerium-based shell. Both the bare nanoparticles and the composite structures did show a high degree of biocompatibility and cell viability: in neither case, the cell morphology altered up to 6 h after addition, a time scale well beyond that of imaging experiments in living cells.^51–53^ As for the decay curves (Figures 9 and S17, Supporting Information), the fitting values for the bare nanoparticles gave *a*_1_ = 76% and τ_1_ = 302 ps for the first component and *a*_2_ = 24% and τ_2_ = 2343 ps for the second component; for the core–shell structures, these values change to *a*_1_ = 71% and τ_1_ = 332 ps for the first component and *a*_2_ = 29% and τ_2_ = 2500 ps for the second one. A longer second component is obtained for the composites, which suggests that the intracellular environment also favors the aggregation of the fluorescent units within cellular compartments.^54^ Generally, nanoparticle endocytosis (including macropinocytosis, which is heavily affected by the physiochemical properties of the NP such as size, charge, and surface coating) and all of these affects in a concerted way the intracellular uptake. Experiments using correlated 2P FLIM and LSM at 4 °C revealed that there is insufficient cellular uptake for both the EuDy:SrALO and the ceria-coated NPs at this temperature to deem the materials traceable in cells, unlike the case of 37 °C. This qualitatively indicates uptake by active transport; however, it is well-known that the size and shape of NPs influence the nature of the uptake mechanism. It is also well documented that NPs with sizes of 100–200 nm enter cells via clathrin- or caveolae-mediated pathways and those of 250 nm to 3–5 μm via phagocytosis or micropinocytosis.^55^ This is believed to result from interactions with cellular membranes, and different endocytosis patterns may well be displayed by the range of the particles involved hereby too. We hypothesize that macropinocytosis, an understudied endocytosis mechanism than the conventional receptor mediated endocytosis, occurs based on the detailed microscopy results and further focused studies are underway in our laboratories.

**Figure 8:**
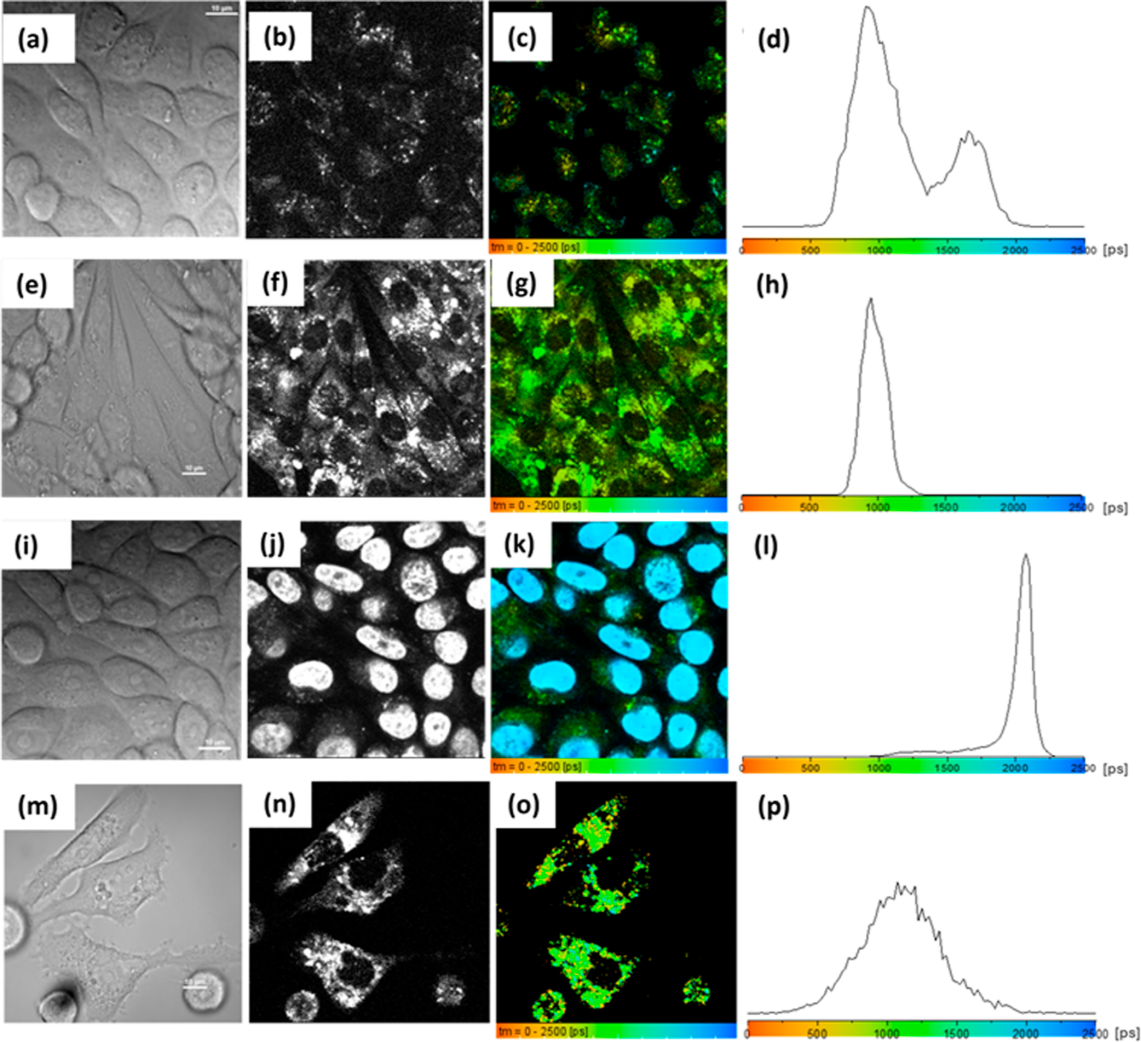
In vitro two-photon fluorescence microscopy of CHO cells. Correlated imaging (scalebar 10 μm) with micrographs showing DIC channel, intensity map, lifetime distribution maps, and associated profiles for lifetime distribution for (a–d) luminescent particles Eu,Dy:SrAlO, (e–h) core–shell particles CeO_2_@Eu,Dy:SrAlO, (i–l) luminescent particles Eu,Dy:SrAlO plus NucBlue CHO cells were treated with 1 mg/mL of the particles. Additional experiments are given in the Supporting Information. In vitro two-photon fluorescence microscopy of PC-3 cells. DIC channel, intensity map, lifetime map, and associated profile distribution for (m–p) core–shell particles CeO_2_@Eu,Dy:SrAlO in PC-3 (37 °C, 15 min incubation). Colors provide a direct correlation between the lifetime maps and the lifetime histograms. Laser power: 2.0 mW at 800 nm wavelength (multiphoton excitation). PC-3 cells were treated with 1 mg/mL of the particles. Additional micrographs are given in the Supporting Information.

**Figure 9:**
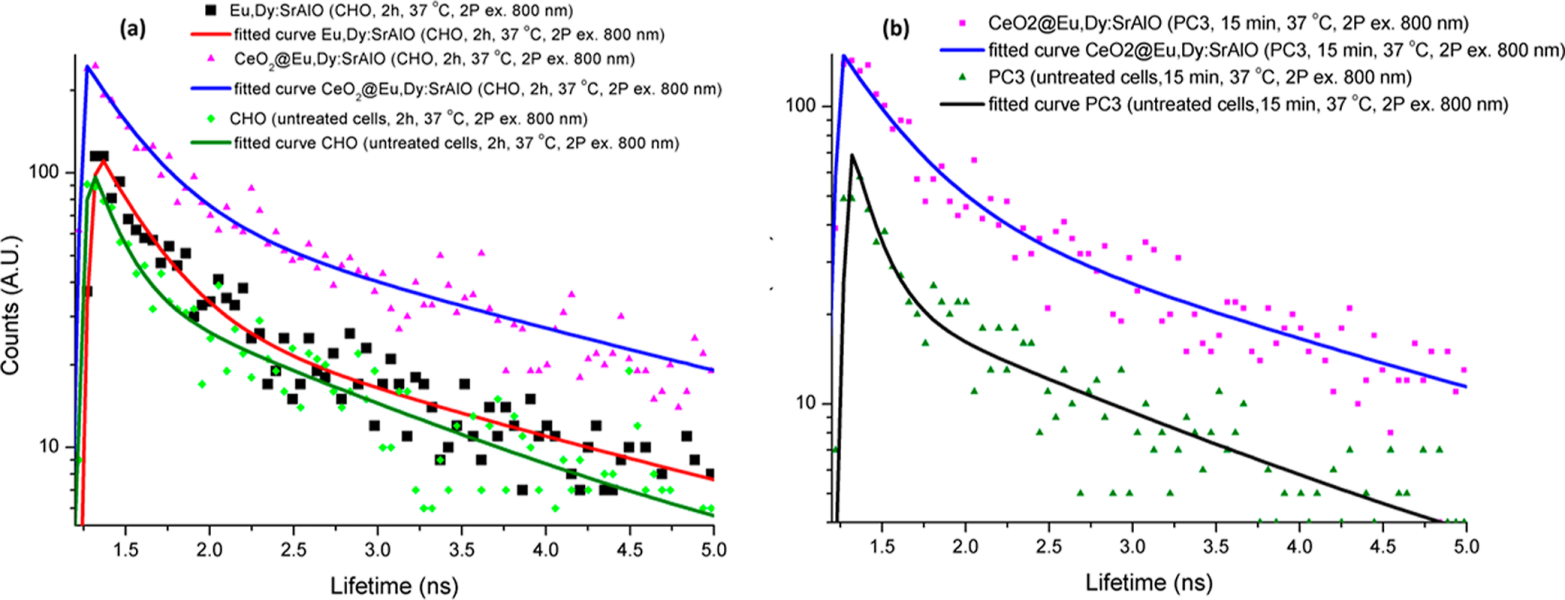
(a) Comparison of 2P fluorescence lifetime decays of Eu,Dy:SrAlO and CeO_2_@Eu,Dy:SrAlO in CHO cells (800 nm, TCSPC measurements, CHO cells were treated with 1 mg/mL of the particles) and (b) comparison of 2P fluorescence lifetime decays of CeO_2_@Eu,Dy:SrAlO in PC-3 cells (800 nm, TCSPC measurements, PC-3 cells were treated with 1 mg/mL of the particles).

Colocalization tests with NucBlue, a nuclear stain with maximum λ_ex_ = 360 nm, λ_em_ = 460 nm, which is a well-known commercial dye (Thermo Fisher) were also carried out as control to better understand the characteristics of the EuDy:SrAlO fluorescent nanoparticles in the presence of such organic dyes alongside their localization. From these tests, depicted in the Supporting Information, it can be concluded that the Eu,Dy:SrAlO distributed mainly in the cytoplasm unlike the NucBlu, which is evidently in the nucleus. In view of these results, a second in vitro test was conducted with the core–shell structures, this time with a PC-3 prostate cancer cell line. The PC-3 cells were grown according to standard serial passage protocols, plated onto glass bottom dishes and allowed to grow up to a suitable confluence. Subsequently, the cancer cells were incubated with a DMSO/RPMI (1:99) serum-free medium suspension of the core–shell units (10 mg/mL). Figures 8m–p and S46–S49 Supporting Information show the corresponding two-photon fluorescence microscopy results, evidencing an effective uptake of the composites in the PC-3 cells. Specifically, the confocal microscopy images reveal that the core–shell structures are mostly located throughout the cell cytoplasm but do not penetrate the nuclear membrane since no emission was observed in the nuclei of the cells. No visible alteration in the cell morphology was apparent up to 6 h after addition, suggesting that the concentration of the ceria core–shell particles used during the experiments is unlikely to produce cellular damage (i.e., the observed internalization is not caused by cellular degradation). Finally, the fluorescence decay is similar to that shown in CHO cells (Figures 9 and S18, Supporting Information), which indicates a propitious robustness of the composite structures for use as CAs in optical imaging. This, added to the presence of the cerium atoms in the shell with their characteristically strong X-ray adsorption capacity for CT applications, could render these as-produced core–shell inorganic nanophosphors promising, highly versatile platforms for future bioimaging applications.

## Materials and Methods

### Synthesis of the Cerium-Coated Core–Shell Structures

For the preparation of the dual-modal core–shell units, a specific sequential procedure was applied, comprising first the synthesis of the luminescent core nanoparticles, then the formation of stable and dispersed suspensions of these nanoparticles, and finally their assembly with the cerium precursor solution (shell and CT CA) using a reverse micelle methodology. The whole procedure is detailed as follows in a stepwise manner. First, the synthesis of the strontium aluminate core luminescent particles was carried out by adapting a hydrothermal synthesis procedure as reported by Xu et al.^56^ Reagent-grade precursors were used, including strontium acetate (Aldrich), aluminum nitrate nonahydrate (VWR), europium­(III) oxide (99.9%, Alfa Aesar), and dysprosium­(III) oxide (99.9%, Alfa Aesar). Prior to the hydrothermal reaction, the rare-earth oxide commercial precursors were solubilized as citrates. In doing so, a mixture of each RE_2_O_3_ powder and citric acid (C_6_H_8_O_7_, 99%, Aldrich) with a 3:1 molar ratio (citric acid/RE^3+^) was added onto a round flask with 4 mL of distilled water, and the mixture was kept all night at 120 °C under reflux and constant stirring. NH_3_ (32%) was then incorporated to increase the pH and dissolve the as-formed citrates, keeping the reflux and agitation for two more hours at 110 °C; the obtained RE citrates were filtered (<100 nm) and the concentrations were determined by inductively coupled plasma optical emission spectrometry. A mixture of all precursors in the desired proportions was subsequently placed in a Teflon beaker, which was sealed and heated for 8 h at 160 °C under autogenerated pressure. The precipitate obtained was cleaned with oleic acid and ethanol by several sonication and centrifugation steps and dried at 60 °C for 24 h. Finally, a thermal treatment was conducted at 1300 °C in reducing atmosphere, bringing the europium activators to the required 2+ oxidation state.

Once the luminescent nanoparticles were obtained, it was mandatory to prepare them in the form of a stable and well-dispersed suspension so that they can be effectively encapsulated by the cerium shell. Otherwise, they would aggregate, making the final size of the core–shell unit too large (>500 nm), ultimately preventing its incorporation into the cells. Accordingly, the nanoparticulate powder obtained after the reductive thermal treatment was ground and sonicated in the presence of oleic acid, a surfactant molecule that prevents the agglomeration of the NPs and results in a stable suspension.

The last step involved the preparation of the core–shell structures using a reverse micelle protocol as adapted from Cui et al.^57^ and Kudo and Miseki.^58^ The routine consisted of slowly dropping the suspension of the synthesized core nanoparticles into a round flask containing a mixture of cerium­(III) nitrate and HMTA previously dissolved in ethanol 96% (both at 0.1 M concentration). The whole system was heated to 60 °C and kept under constant stirring for 15 h, after which the suspension produced was rinsed with ethanol by sonication and centrifugation and then dried at 60 °C for 24 h to obtain the final product.

### Characterization in Dispersed and Solid State and on the Nanoscale

The analyses of the crystalline structure and phase identification were performed by X-ray diffraction (XRD Bruker D8 ADVANCE) with a monochromatized source of CU-Kα1 radiation (λ = 1.5406 nm) at 1.6 kW (40 kV, 40 mA); samples were prepared by placing a drop of a concentrated ethanol dispersion of particles onto a single crystal silicon plate. Field emission scanning electron microscopy (FESEM) was employed to characterize the main microstructural features of the particles; a cold FESEM Hitachi S-4700 microscope was used for that purpose. HRTEM images were obtained on a JEOL 2100F transmission electron microscope (TEM/STEM) operating at 200 kV and equipped with a field emission electron gun providing a point resolution of 0.19 nm; samples were prepared by placing a drop of a dilute ethanol dispersion of nanoparticles onto a 300 mesh carbon-coated copper grid and evaporating immediately at 60 °C. The precise concentration of the rare-earth elements in each precursor solution was determined by ICP-AES using an Optima 3000 equipment, PerkinElmer. The thermogravimetric analyses of the precursors were carried out in a TGA furnace of TA Instruments, TGA 951-2000. The concentration of the suspensions of the as-synthesized nanoparticles was also determined by TGA. Particle size distribution was measured by DLS at 25 °C using a Malvern Zetasizer Nano ZS instrument. UV–vis diffuse reflectance spectroscopy (DRS UV–vis) was carried out in the solid state on a Analytik Jena Specord 200 plus spectrometer equipped with an integrating sphere. Fluorescence emission spectra and quantum yield measurements were acquired with an Edinburgh Instruments FS5-spectrofluorimeter equipped with an integration sphere.

### General Cellular Culturing Details

Cells were cultured at 37 °C in a humidified atmosphere in the air and subcultured once confluence had been reached. Culture occurred in Eagle’s Minimum Essential Medium (EMEM) containing 15% fetal calf serum (FCS), 0.5% penicillin/streptomycin, and 1% l-glutamine. The surplus supernatant containing dead cell matter and excess protein were aspirated. The live adherent cells were then washed with 2 × 10 mL aliquots of phosphate buffer saline (PBS) solution to remove any remaining media containing FCS, which inactivates trypsin. Cells were resuspended in solution by incubation in 3 mL of trypsin–PBS solution (0.25% trypsin) for 5 min at 37 °C. After trypsinization, 5 mL of medium containing serum was added to inactivate the trypsin, and the solution was centrifuged for 5 min (1000 rpm, 25 °C) to remove any remaining dead cell matter. The supernatant liquid was aspirated, and 5 mL of medium was added to the cell matter left behind. Cells were counted using a hemocytometer and then seeded as 0.15 million cells in the absence of indicator dyes such as phenol red in cell medium (15% FCS), for 48 h in poly-d-lysine-coated dishes. Cells were washed in serum-free medium three times prior to the addition of particles. For each experiment, images of cells were taken prior to addition, which indicate that cells were healthy, suitable for nanoparticle addition, given this initial perfectly undamaged outer cellular membranes and low background fluorescence.

#### Cellular Viability Tests

Standard MTT assays of PC-3 cells treated with composite were performed in order to investigate the effect of the Eu,Dy:SrAlO alone and post CeO_2_ encapsulation, giving rise to CeO_2_@ Eu,Dy:SrAlO cellular viability.^59^ Two different batches of ceria-coated platelets were tested, and the results demonstrate that all the nanoplatelet materials tested were biocompatible and the encapsulation of Eu,Dy:SrAlO within a ceria shell improves the in vitro biocompatibility. Normalized cell viability was evaluated in PC-3 cells treated with 1 pg/mL–20 μg/mL Eu,Dy:SrAlO and CeO_2_@ Eu,Dy:SrAlO. Control cells seed and grown for 72 h at 37 °C, cells treated with either Eu,Dy:SrAlO or CeO_2_@ Eu,Dy:SrAlO were incubated for 72 h at 37 °C, and 5 mg/mL MTT reagent was incubated for 3 h. Error bars stand for standard error calculated from the 12 repeats. Experiments shown these nanomaterials are nontoxic on the time scale of the cellular imaging investigations.

#### Cells Culturing for 2P FLIM

Cells for the 2P FLIM measurements were cultured according to a standard protocol. Briefly, cell lines used for live cell imaging were prostate cancer cells (PC-3) or Chinese hamster ovary cells (CHO) as healthy controls. All cell lines were obtained from American Type Cell Culture and stored frozen at −196 °C in liquid nitrogen until required, then thawed quickly, and incubated after the addition of fresh media at 37 °C under a 5% carbon dioxide environment. All solvents, buffer solutions, and media mentioned in the following section were warmed to 37 °C in the water bath prior to addition. Eagle’s Minimum Essential Medium (EMEM) and Roswell Park Memorial Institute (RPMI) medium were used as culture media. All media contained activated FCS (10%), 0.5% penicillin/streptomycin (10,000 IU mL-1/10,000 mg mL-1), and 2.5% l-glutamine.

Cell subculture was performed once or twice per week depending on the confluence of the cells in the flask, and the supernatant was aspirated. All attached cells were washed twice using phosphate buffered saline (PBS, 2 × 10 mL), and 3.5 mL of 0.25% trypsin in PBS was subsequently loaded and incubated at 37 °C for 5 min. After trypsinization, 7 mL of serum medium was added to neutralize the excess trypsin and the solution was centrifuged at 1000 rpm for 5 min. Afterward, the supernatant was aspirated and resuspended with 5 mL of serum medium. Prior to microscopy experiments, cells were seeded onto sterile glass dishes and incubated for 48 h prior to addition of fluorescent compounds to allow them to adhere to the surface.

### MP FLIM and PLIM In Vitro Experiments

A custom multiphoton–photon confocal FLIM system was constructed around a Nikon Ti2 inverted microscope with an attached modified Nikon EC2-Si scan head. The confocal scanning system was modified to enable near-infrared laser wavelength transmission.^60^ A mode-locked tunable laser, 660–1320 nm (Chameleon Discovery NX, Coherent Lasers) was used to provide laser light at wavelengths of 800 and 910 nm with a 100 fs pulse width at 80 MHz. The samples were illuminated on the microscope stage using a water-immersion 60× objective (Nikon VC; numerical aperture of 1.27).

Fluorescence emission was collected without a pinhole (nondescan mode), bypassing the confocal scanning system and is passed through a BG39 (Comar) and 700 nm short-pass filter to block the near-infrared laser light. Line, frame, and pixel clock signals are generated and synchronized with an external detector in the form of a fast hybrid photomultiplier tube (HPM100-40, Becker and Hickl, GmbH). The scanning system was linked to a TCSPC PC module SPC830 (Becker and Hickl) to generate the raw time-correlated single-photon (TCSPC) decay at each pixel or a single decay curve for solution phase studies. Analysis of the pixel-by-pixel TCSPC data (using SPCImage, V8.6 software) generated a FLIM image or map. Prior to FLIM data, the instrument was calibrated using samples with well-known lifetimes such as fluorescein, rhodamine b, and 7-hydroxycoumarin carboxylic acid. PLIM under 400 nm excitation was performed on a similar setup as it has the same principle as FLIM, but because this technique measures emission on a much longer time scale, it is compatible with long-lived emissive triplet states, which we anticipated would be suitable to the same materials.^51^

Decay data were mostly fitted to a single exponential parameter as *f*(*t*) = *a*_*i*_·*e* – τ_*i*_/τ_*i*_, eq 2 above. A chi-square (*c* ^2^) value is used to determine the goodness of fit where values with a χ^2^ between 0.9 and 1.2 are considered excellent decay fitting. A higher χ^2^ value (>1.4) following a single exponential fit indicated the need for multiple exponential decay presence (*i* > 1).

## Conclusions

New core–shell composite nanoparticles have been synthesized by combining two materials with suitable properties to operate as potential CAs (doped SrAlO and ceria) toward two complementary imaging modalities, namely, optical imaging and CT. The multicomponent platforms were produced by a simple reverse micelle procedure by which suitably doped strontium aluminate nanoparticles acting as luminescent cores (Eu,Dy:SrAlO) were encapsulated on a cerium oxide based shell to give rise to CeO_2_-coated Eu,Dy:SrAlO with enhanced biocompatibility and can simultaneously act as a potential X-ray attenuator for CT applications and is traceable within living cells on the basis of intrinsic fluorescence and phosphorescence. Moreover, the coating with the thin ceria shell not only retains the optical integrity of the luminescent cores but also reduces emission losses caused by surface quenching effects. The as-obtained hybrid heterostructures are observed to be fully dispersible and kinetically stable in aqueous media, holding an average size well below 300 nm that enables their effective incorporation into the cells. This was confirmed for the first time for materials of this family by two-photon fluorescence microscopy analyses and in vitro studies with different living cell lines, demonstrating the viability and robustness of the inorganic core–shell composites for bioimaging applications.

## Supplementary Material



## Notes

Characterization of Eu,Dy:SrAlO platelet composites, characterization of the CeO2 shell, characterization of the CeO_2_@Eu,Dy: SrAlO core–shell platelet nanocomposites, two-photon fluorescence lifetime spectroscopy (TCSPC) and imaging microscopy (MP FLIM and PLIM), general cell culturing methods for fluorescence imaging, and cellular viability tests (PDF)
