# Supplementary material for: Rare Earths-Doped and Ceria-Coated Strontium Aluminate Platelets—Versatile Luminescent Platforms for Correlated Lifetime Imaging by Multiphoton FLIM and PLIM
Source: ACS Omega. 2025 Apr 29;10(19):19950–65. doi: 10.1021/acsomega.5c01649 (PMC12096198; doi:10.1021/acsomega.5c01649)
Supplement: Supplementary file 1 [file ao5c01649_si_001.pdf]

Rare Earths Doped and Ceria Coated Strontium Aluminate Platelets - Versatile Luminescent Platforms for Correlated Lifetime Imaging by Multiphoton FLIM and PLIM

David G. Calatayud,[a,b] María Victoria Martín Arroyo,[a] Amador C. Caballero,[a] Marina Villegas,[a] Haobo Ge,[c] Stanley W. Botchway,[d] Sofia I. Pascu,*[c] Marco Peiteado[a] and Teresa Jardiel *[a]

[a.] *Electroceramics Department, Instituto de Cerámica y Vidrio – CSIC, Kelsen 5, Campus de Cantoblanco, 28049, Madrid, Spain*
[b.] *Inorganic Chemistry, Universidad Autonoma de Madrid, Francisco Tomas y Valiente 7, Campus de Cantoblanco, 28049, Madrid, Spain.*
[c.] *Department of Chemistry, University of Bath, BA2 7AY, Bath, UK.*

Table of Contents:

1. Characterisation of Eu,Dy:SrAlO platelet composites S2
2. Characterisation of the CeO_2 shell S5
3. Characterisation of the CeO_2@Eu,Dy: SrAlO core-shell platelet nanocomposites S7
4. Two-Photon Fluorescence Lifetime Spectroscopy (TCSPC)
   and Imaging Microscopy (MP FLIM and PLIM) S9
5. General Cell culturing methods for fluorescence imaging S26
6. Cellular Viability Tests S26

1. Characterisation of Eu,Dy:SrAlO composites

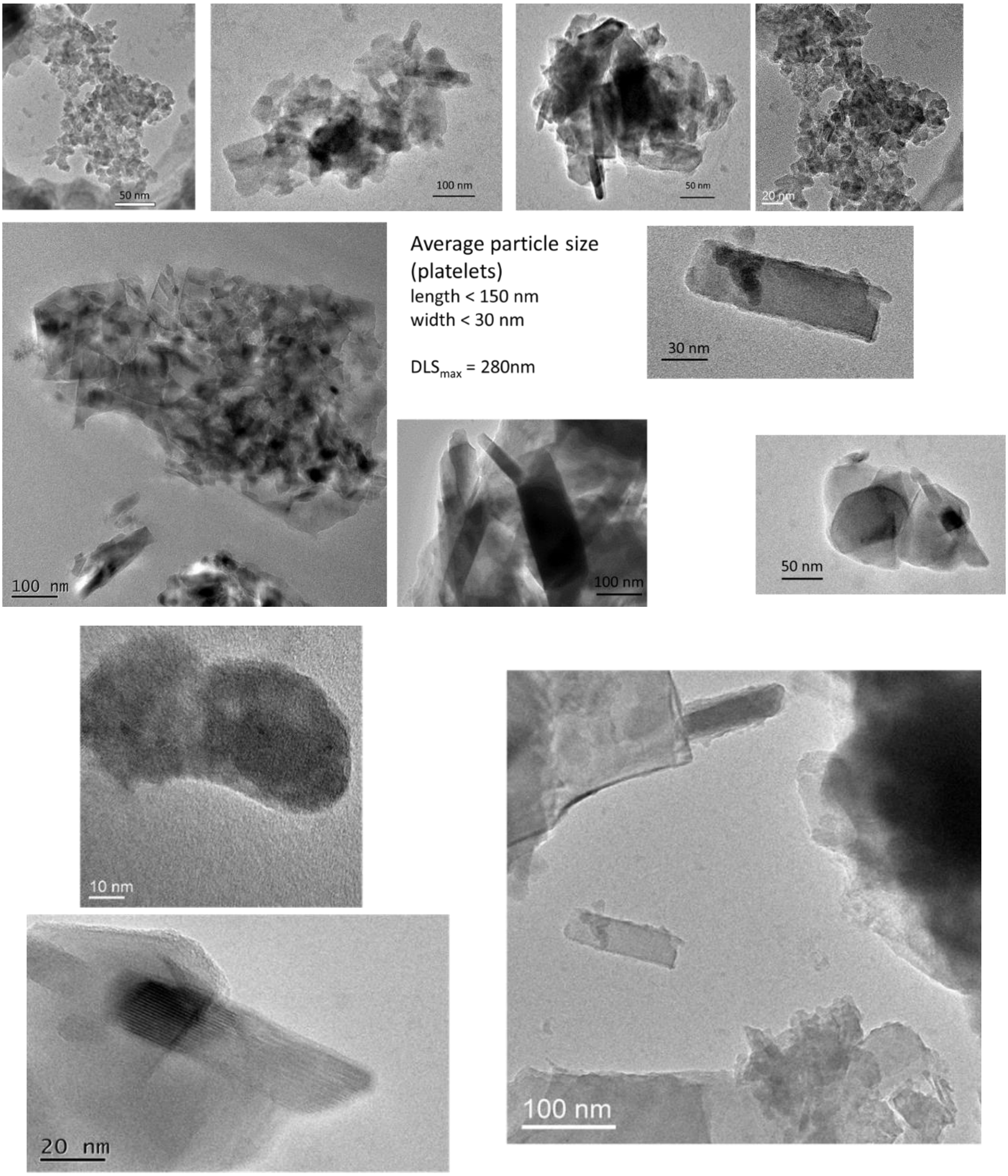

Figure S1. A range of TEM micrographs of the Eu,Dy:SrAlO nanoparticulate cores showing platelets over a range of magnifications

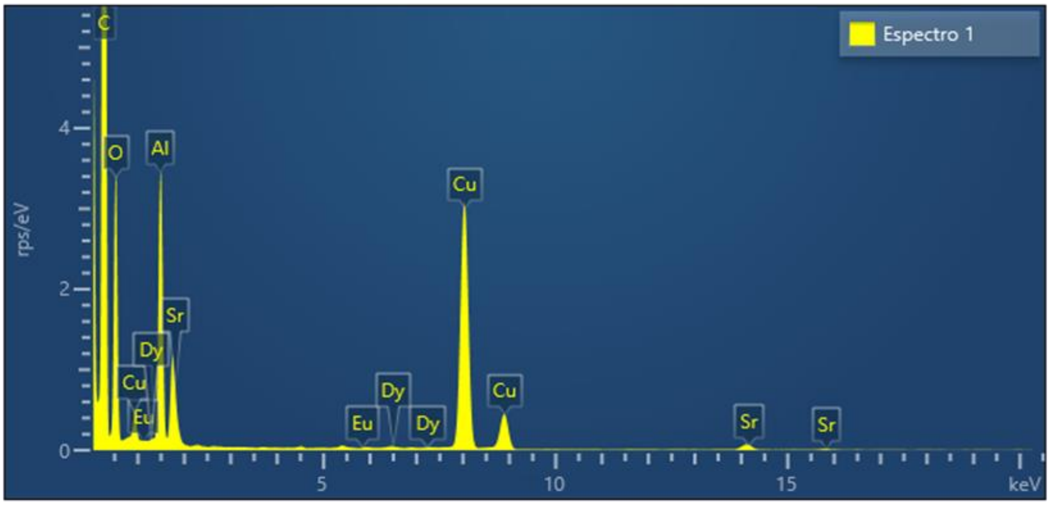

Figure S2. EDX analysis of Eu,Dy:SrAlO core platelets

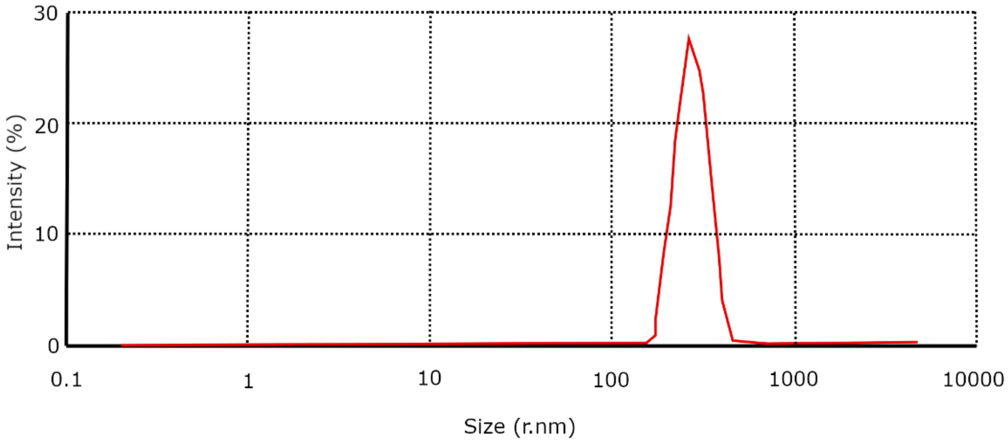

Figure S3. DLS size particle distribution of Eu,Dy:SrAlO in 0.5 mg/L H_2O dispersions

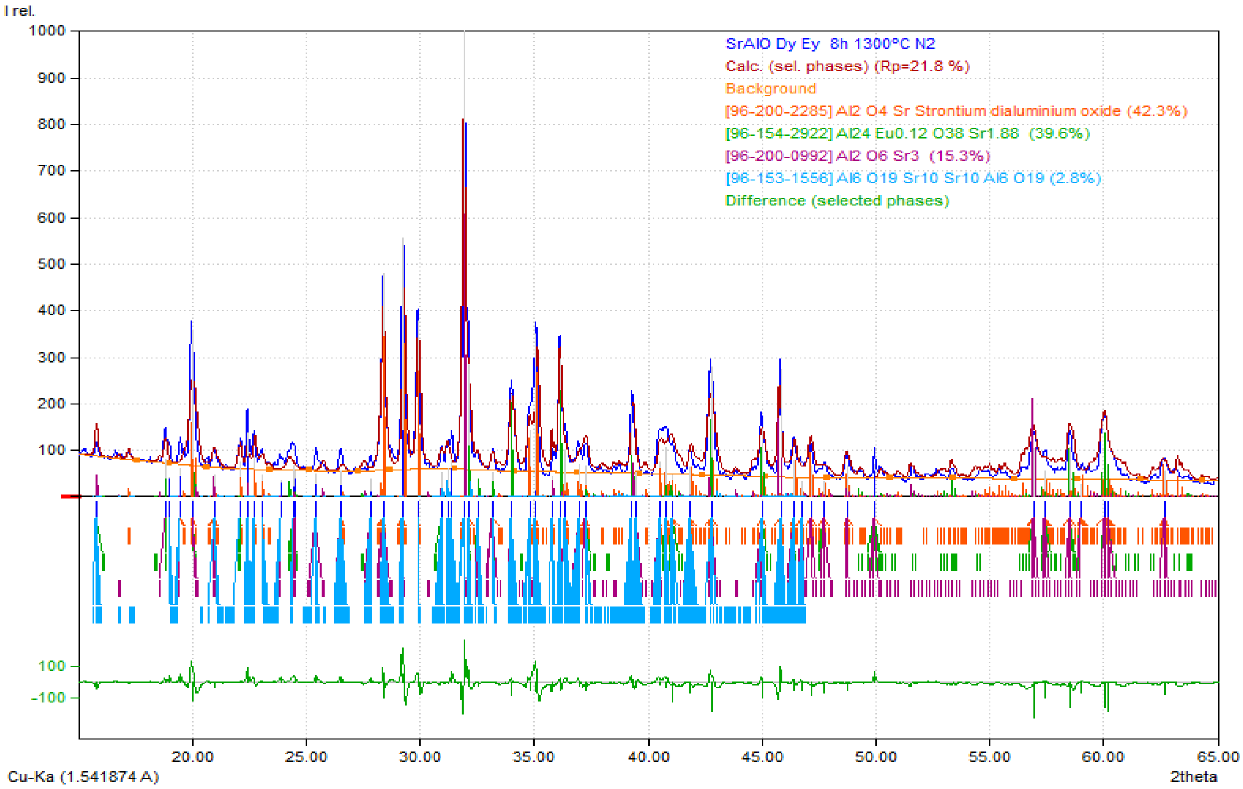

Figure S4. Powder X-ray diffractogram of Eu,Dy:SrAlO solid phase nanoparticulate cores

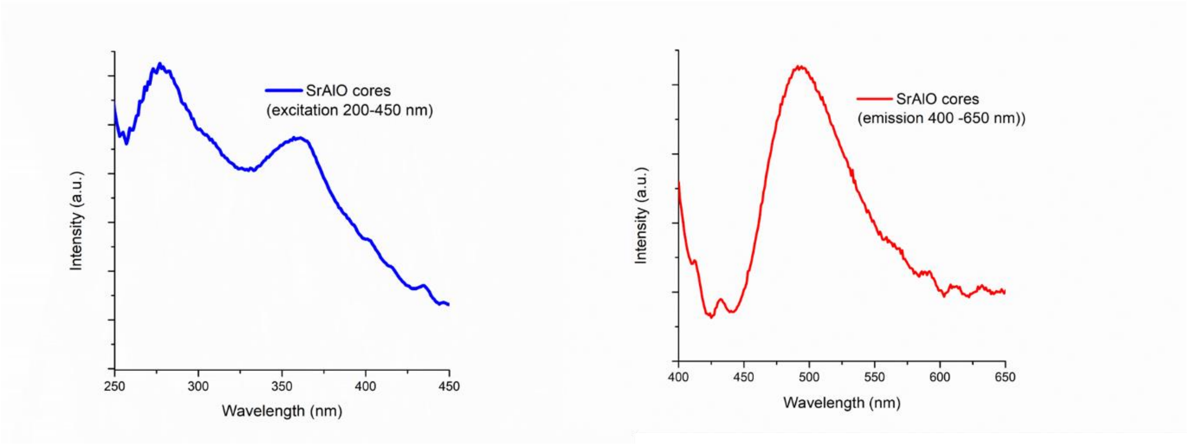

Figure S5. 1-photon fluorescence excitation and emission spectra of Eu,Dy:SrAlO (λ_{exc} = 360 nm, λ_{em} = 505 nm) in thin film

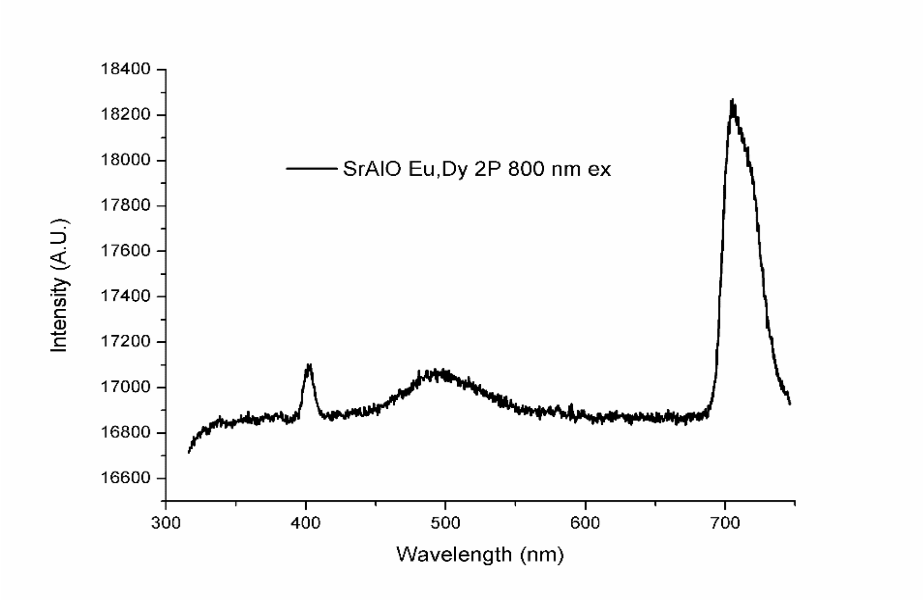

Figure S6. 2-photon fluorescence emission spectrum of Eu,Dy:SrAlO in dispersed phase (1 mg/mL in H_2O).

2. Characterisation of the CeO_2 shell

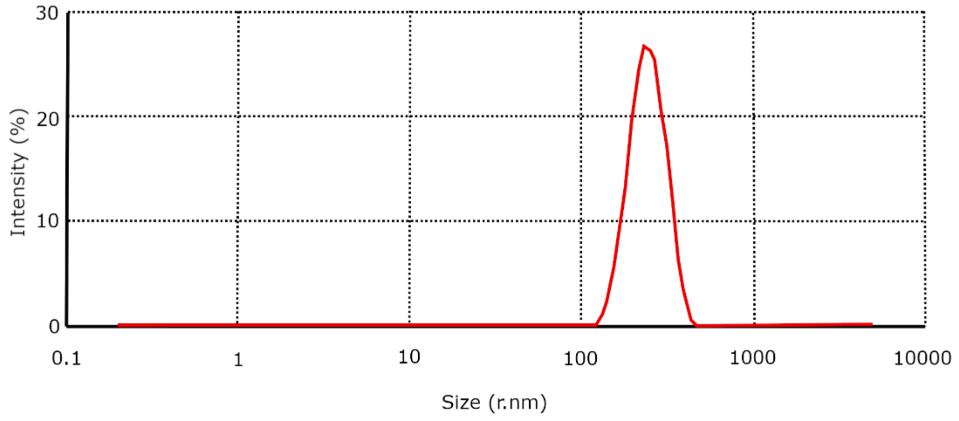

Figure S7. DLS size particle distribution of CeO_2 recorded in 0.5 mg/L H_2O dispersions.

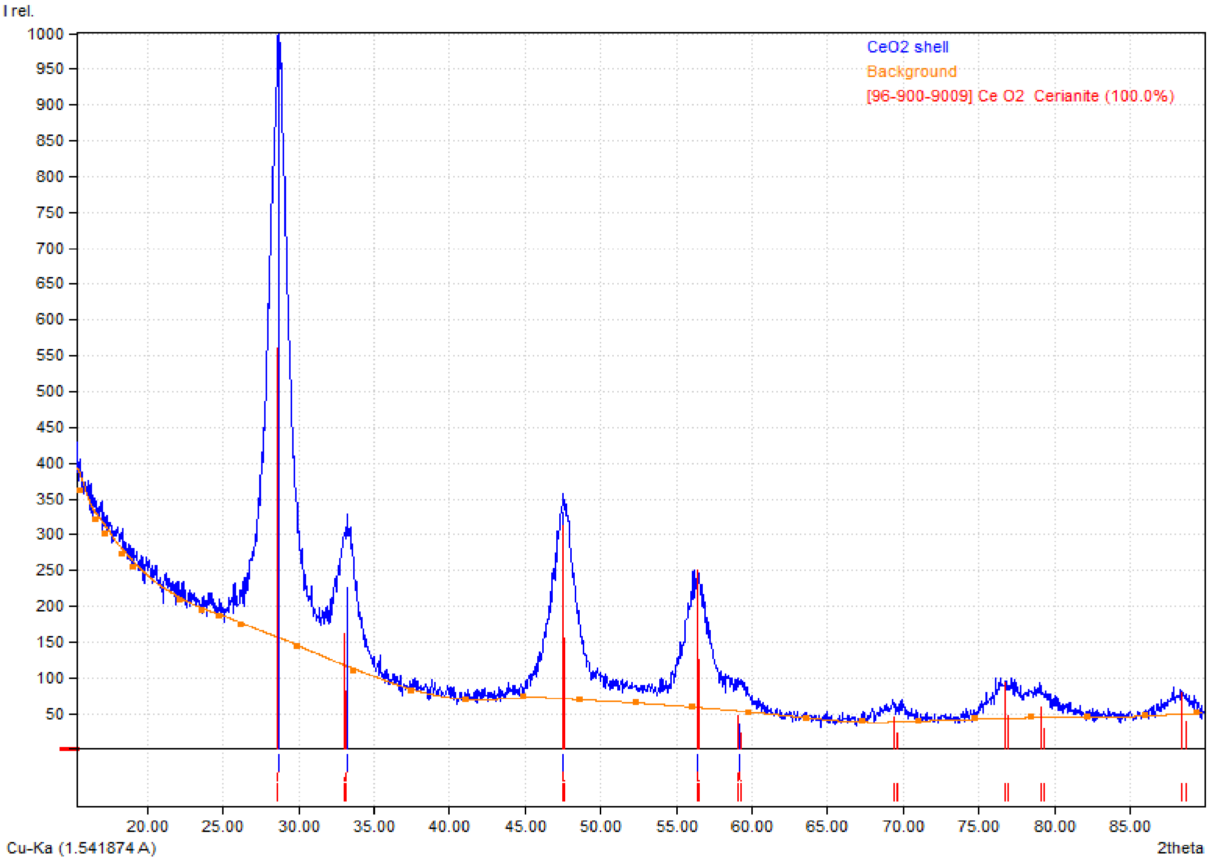

Figure S8. Powder X-ray diffractogram of the CeO_2 nanoparticulate shell

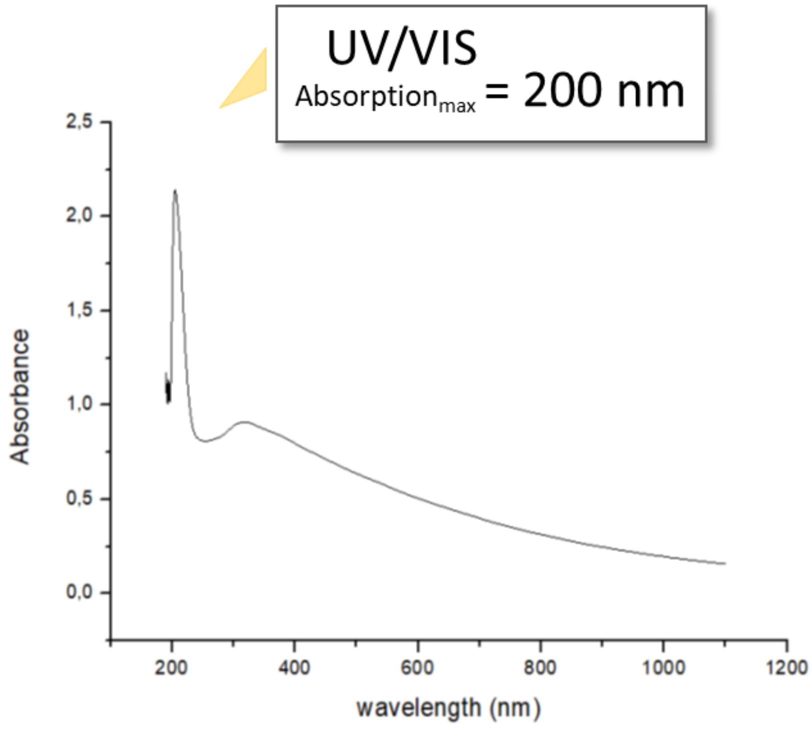

Figure S9. UV-vis spectrum of CeO_2 particles recorded from thin film.

3. Characterisation of the CeO_2@ Eu,Dy:SrAlO core-shell nanocomposites

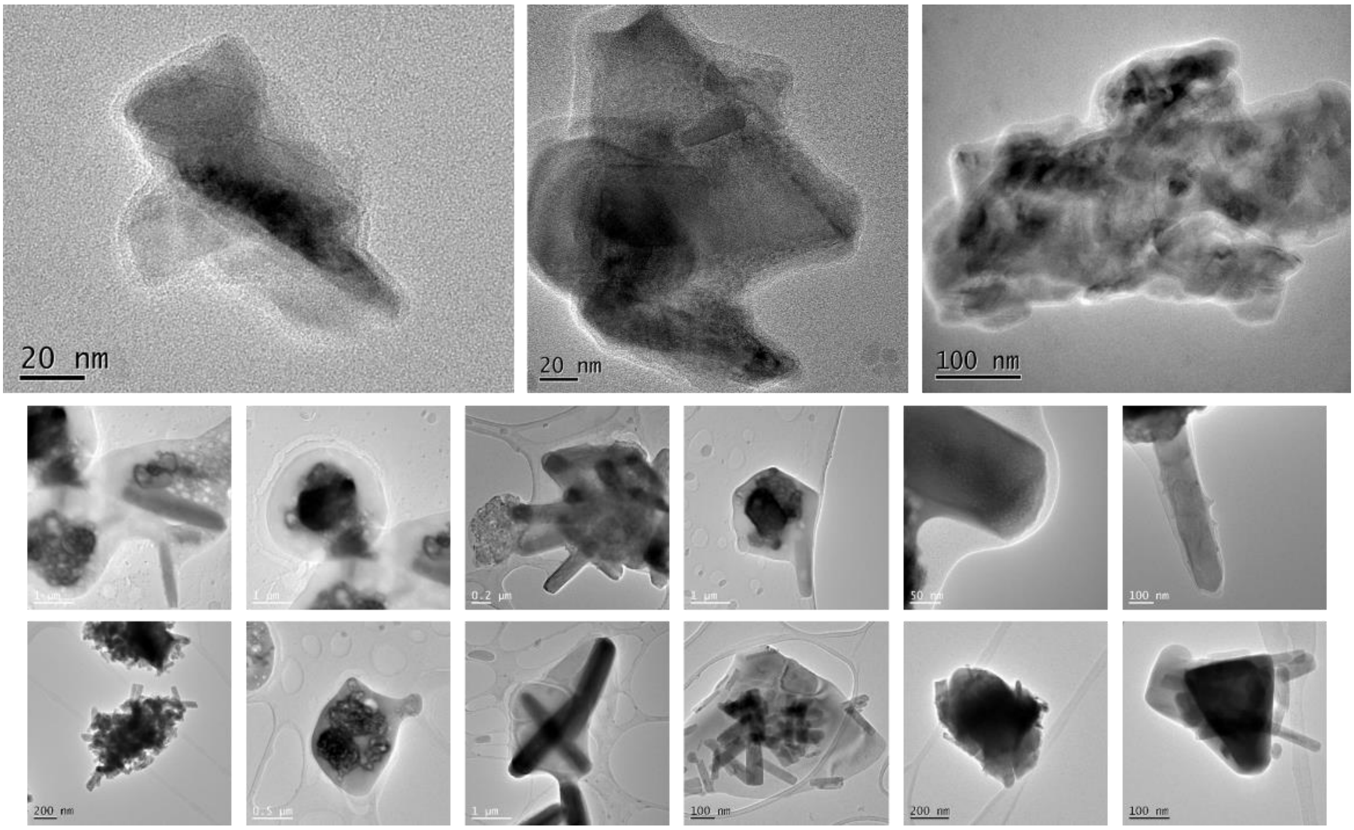

Figure S10. TEM micrographs of the CeO_2@Eu,Dy:SrAlO showing ceria-coated nanoplatelets

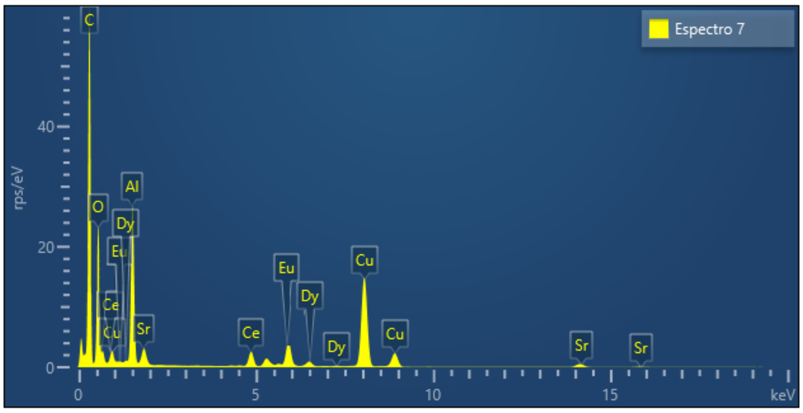

Figure S11. EDX analysis of CeO_2@Eu,Dy:SrAlO core-shell nanoparticles

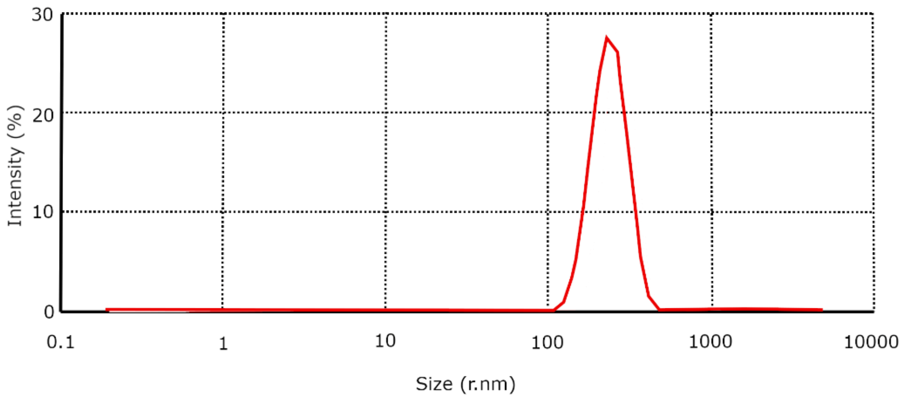

Figure S12. DLS size particle distribution of CeO_2@ Eu,Dy:SrAlO recorded in 0.5 mg/L H_2O.

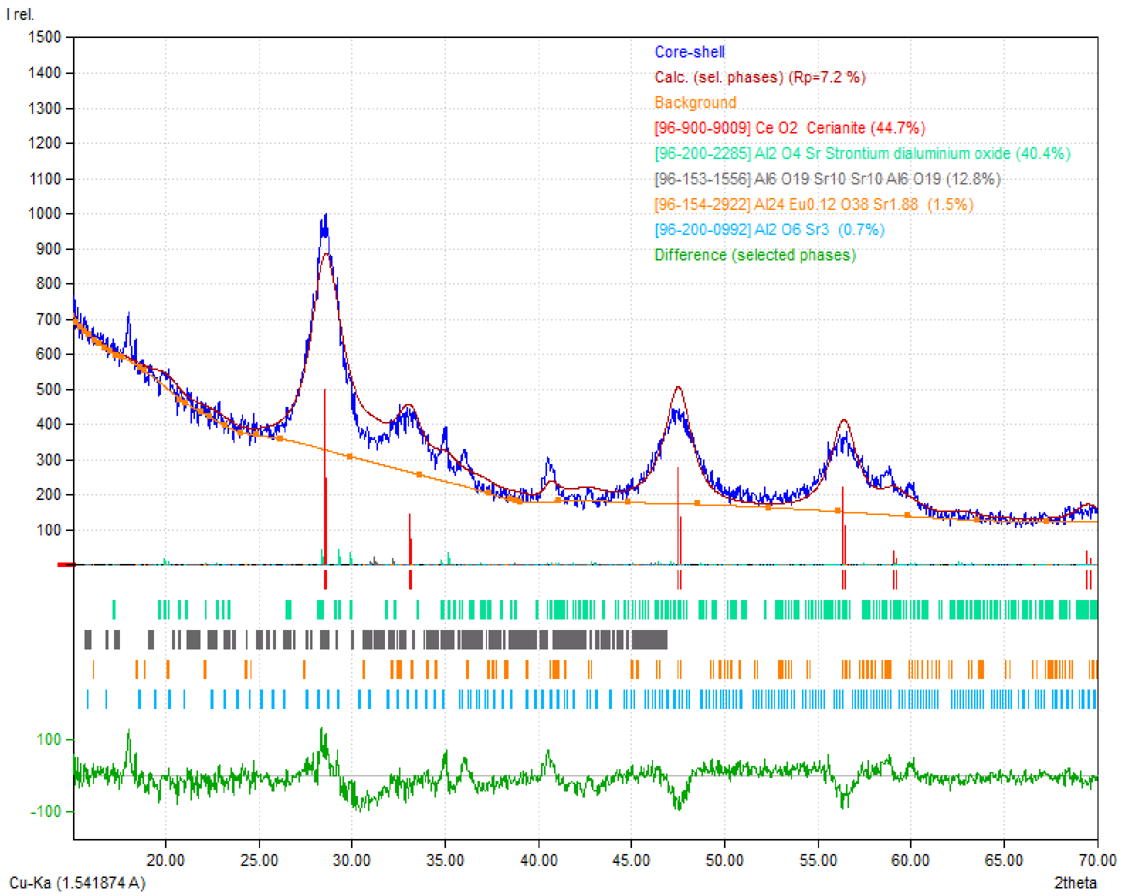

Figure S13. Powder X-ray diffractogram of CeO_2@ Eu,Dy:SrAlO

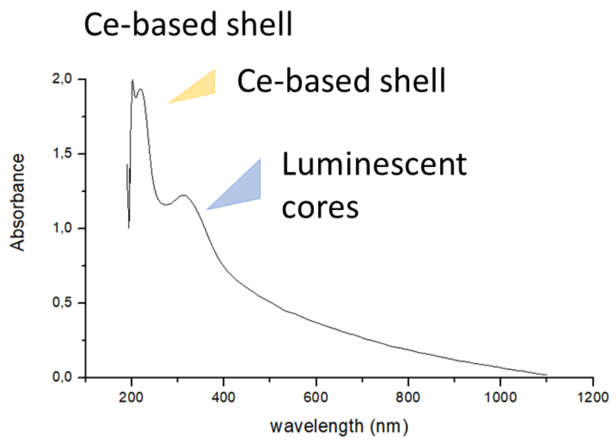

Figure S14. UV-vis spectrum of CeO_2@Eu,Dy: Eu,Dy:SrAlO recorded in thin film.

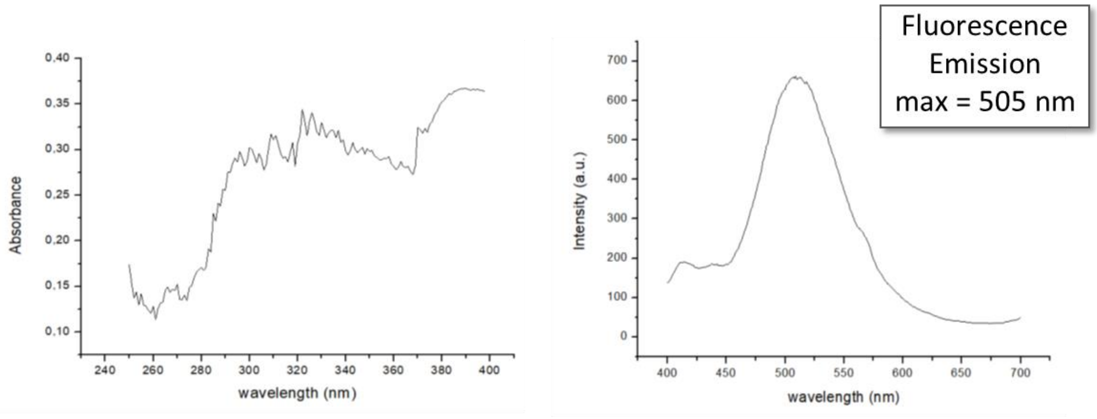

Figure S15. 1-photon fluorescence excitation and emission spectra of CeO_2@Eu,Dy: Eu,Dy:SrAlO (λ_{exc} = 360 nm, λ_{em} = 505 nm) recorded in thin film.

4. Two-Photon Fluorescence Lifetime Spectroscopy (TCSPC) and Imaging Microscopy (MP FLIM and PLIM)

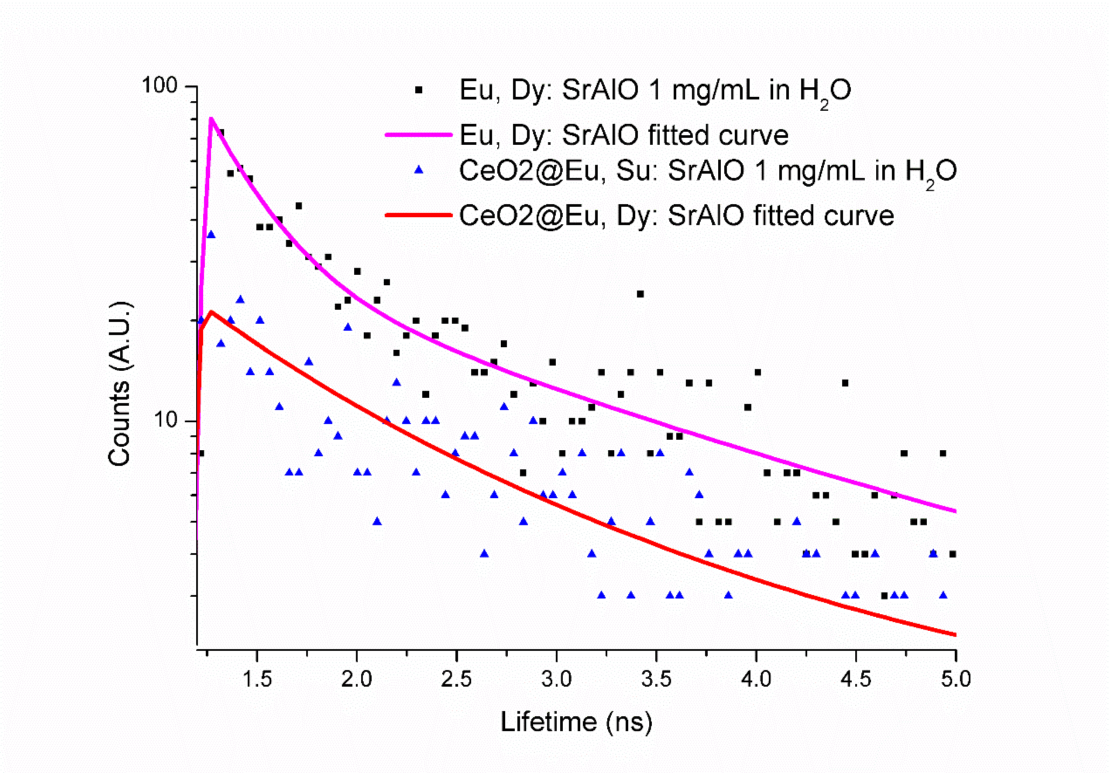

Figure S16. Comparison of 2P fluorescence lifetime decays of Eu,Dy: Eu,Dy:SrAlO and CeO_2@Eu,Dy: Eu,Dy:SrAlO (2-photon excitation 800 nm, TCSPC measurements) each recorded in the dispersed phase (1 mg/mL H_2O). Lifetime determined in droplet of dispersed NPs: Eu,Dy:SrAlO: τ_1 0.16 ns (a1 50%) and τ_2 1.26 ns (a2 50%,) χ^2 =1.15 and CeO_2@Eu,Dy: Eu,Dy:SrAlO: τ_1 0.18 ns (a1 76%) and τ_2 1.55 ns (a2 24%,) χ^2 =1.22.

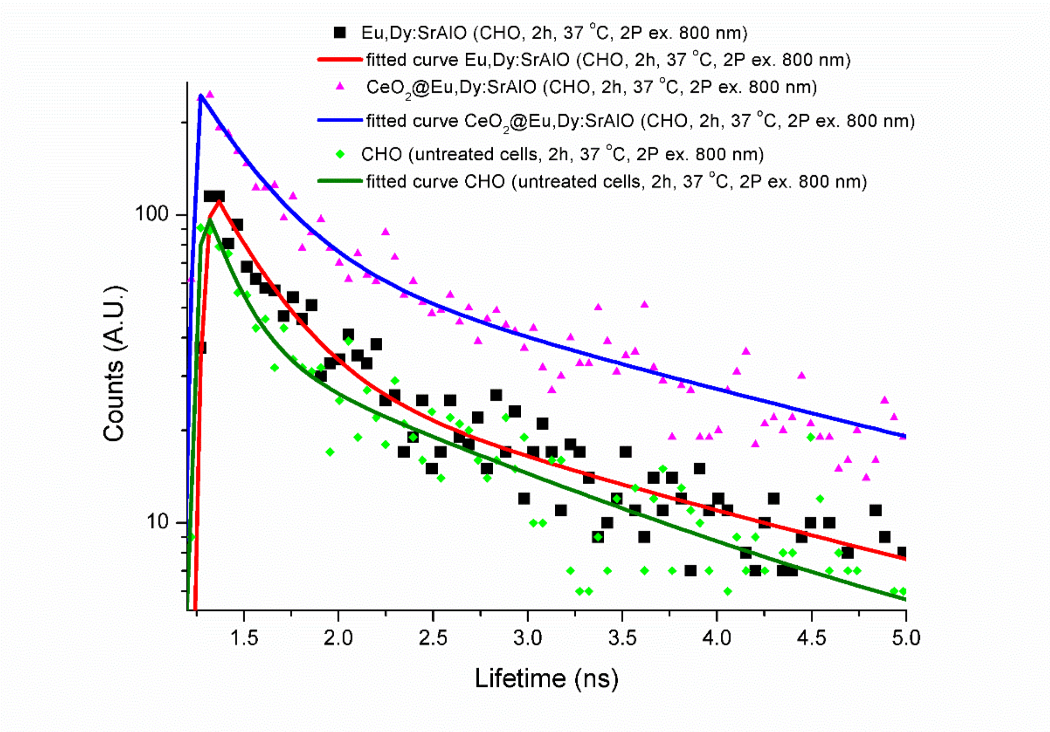

Figure S17. Comparison of 2P fluorescence lifetime decays of Eu,Dy:SrAlO and CeO_2@Eu,Dy:SrAlO in selected spots within CHO cells (800 nm, TCSPC measurements, CHO cells were treated with 1 mg/mL H_2O dispersions of the particles). Raw data and fitted parameters are given in Figure S30 (Eu, Dy: Eu,Dy:SrAlO in CHO),Figure S40 (CeO_2@EuDy; Eu,Dy:SrAlO in CHO) and Figure S47 (untreated CHO cells, control).

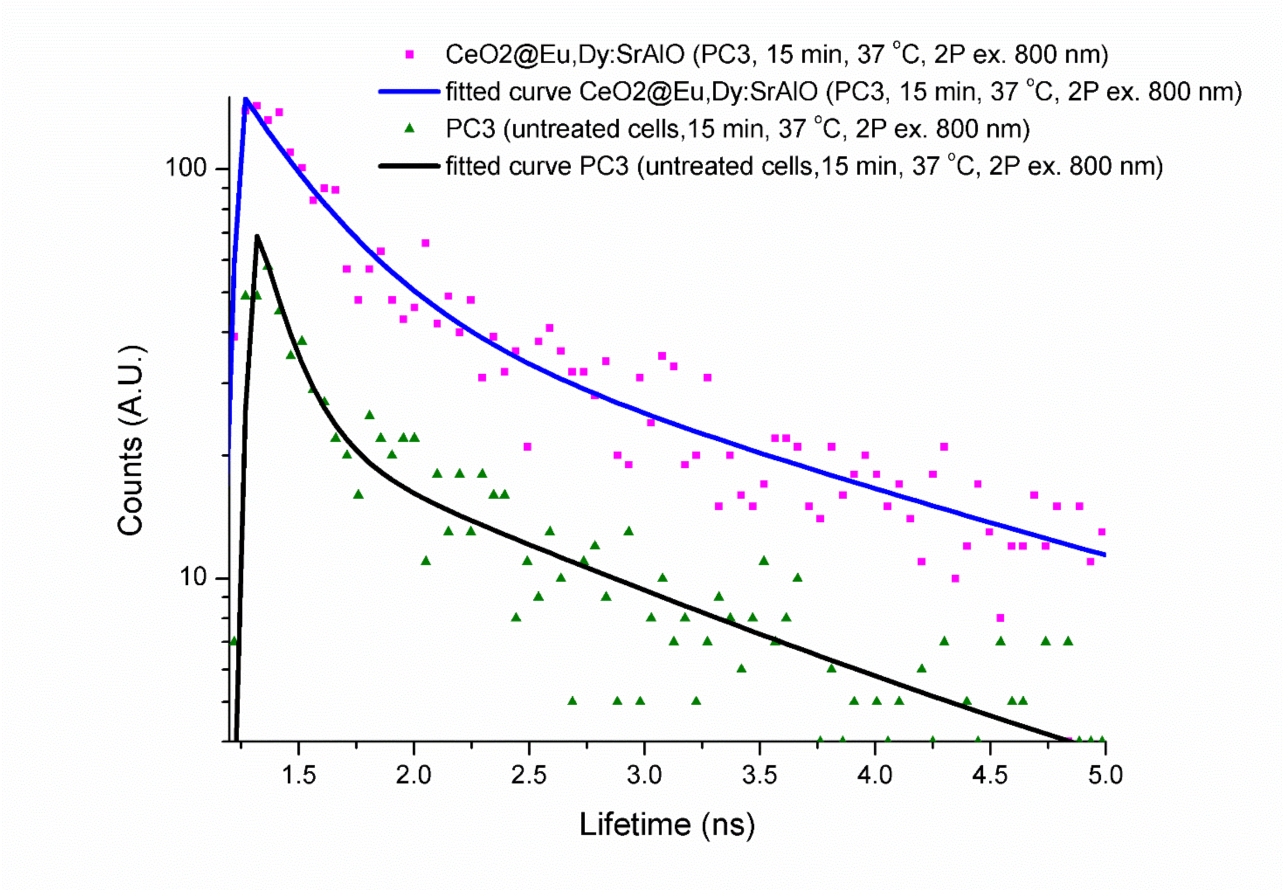

Figure S18. Comparison of 2P fluorescence lifetime decays of CeO_2@ Eu,Dy:SrAlO in PC-3 cells with untreated PC3 cells (2-photon excitation 800 nm, TCSPC measurements, cells were treated with 1 mg/mL of the particles dispersed in H_2O).). Raw data and fitted parameters are given in S45 (CeO_2@Eu,Dy:SrAlO in PC-3) and S48 (untreated PC-3 cells).

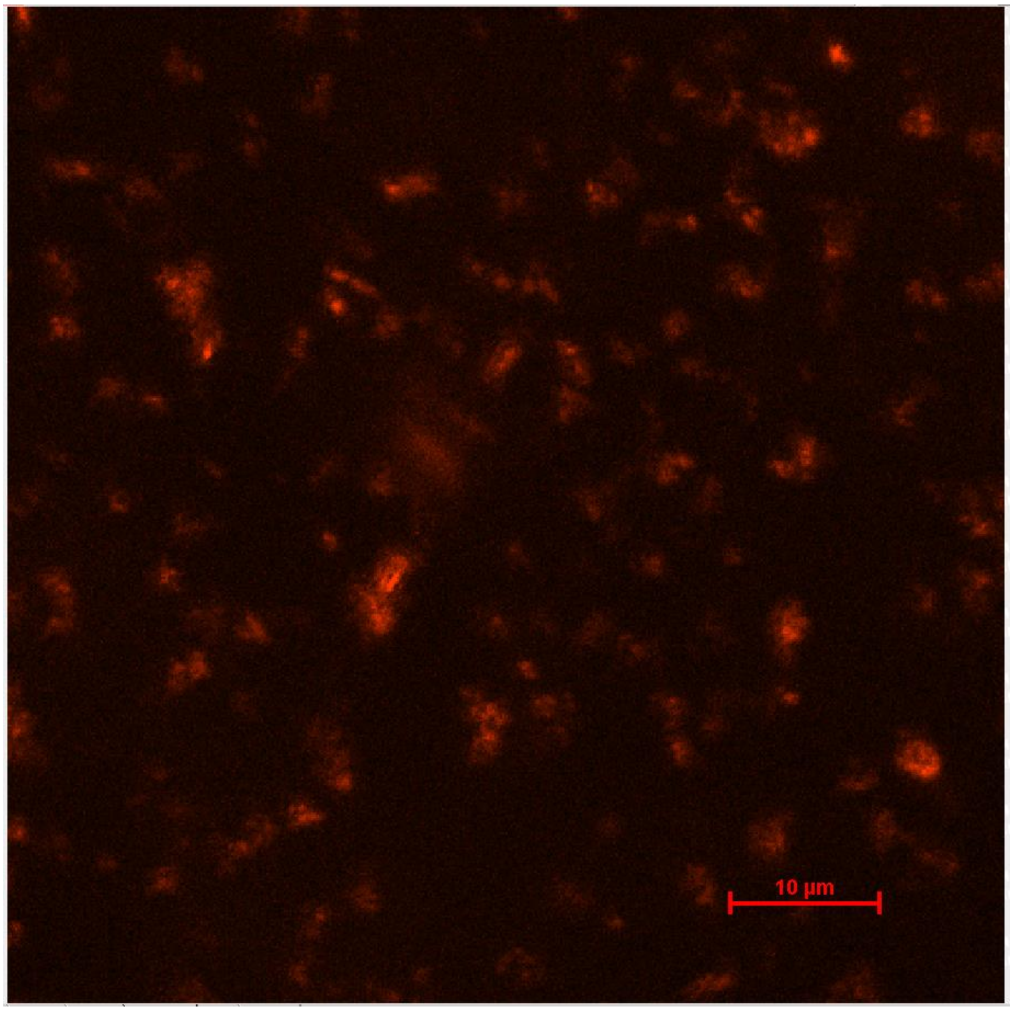

Figure S19. Confocal fluorescence imaging of Eu,Dy:SrAlO in thin film (ex 400 nm, em 605-675 nm)

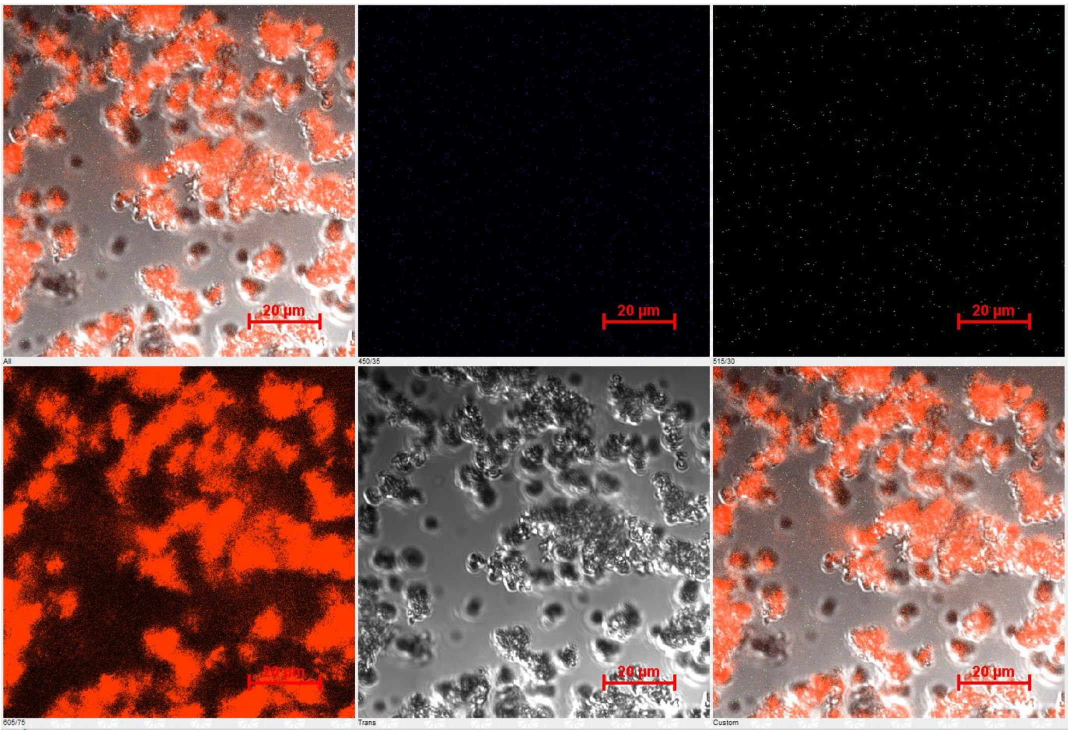

Figure S20. Confocal fluorescence imaging of CeO_2@ Eu,Dy:SrAlO in thin film (ex 400 nm, em 605-675 nm and corresponding overlays with bright field imaging. No emission was observed in the blue (435-450 nm) and green (515-530 nm) emission channels)

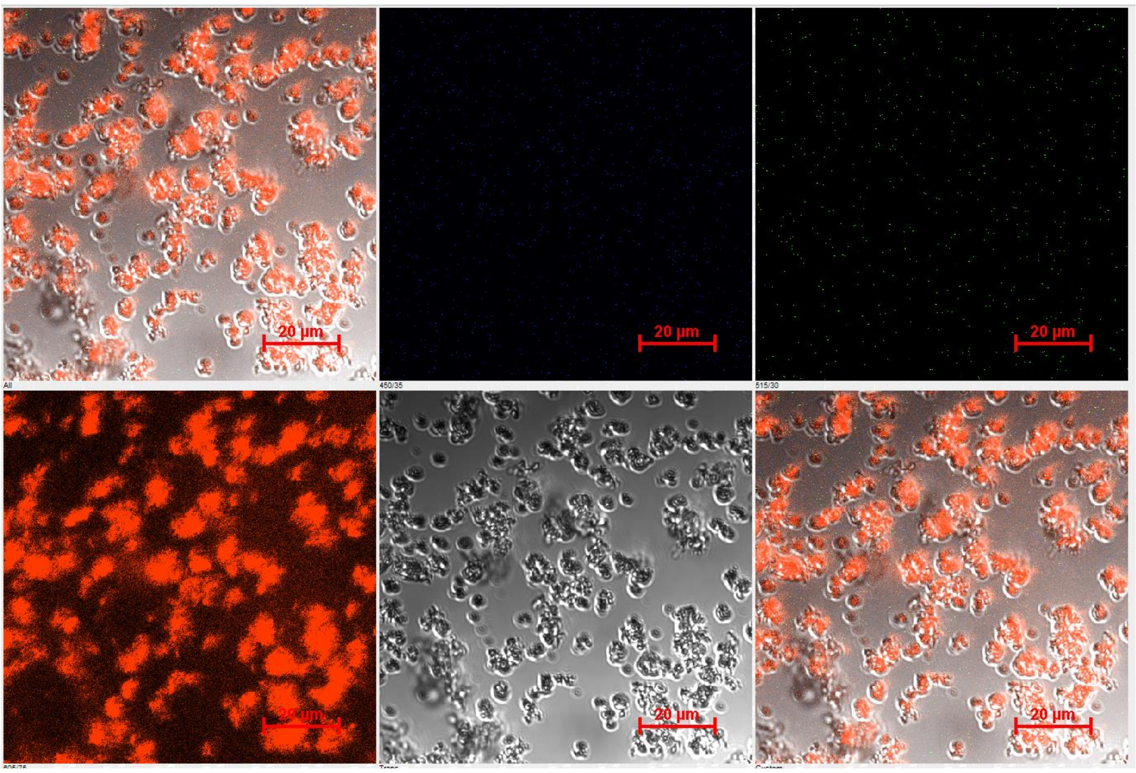

Figure S21. Alternative micrographics for the confocal fluorescence imaging of CeO_2@ Eu,Dy:SrAlO in thin film (ex 400 nm, em 605-675 nm and corresponding overlays with bright field imaging. No emission was observed in the blue (435-450 nm) and green (515-530 nm) emission channels).

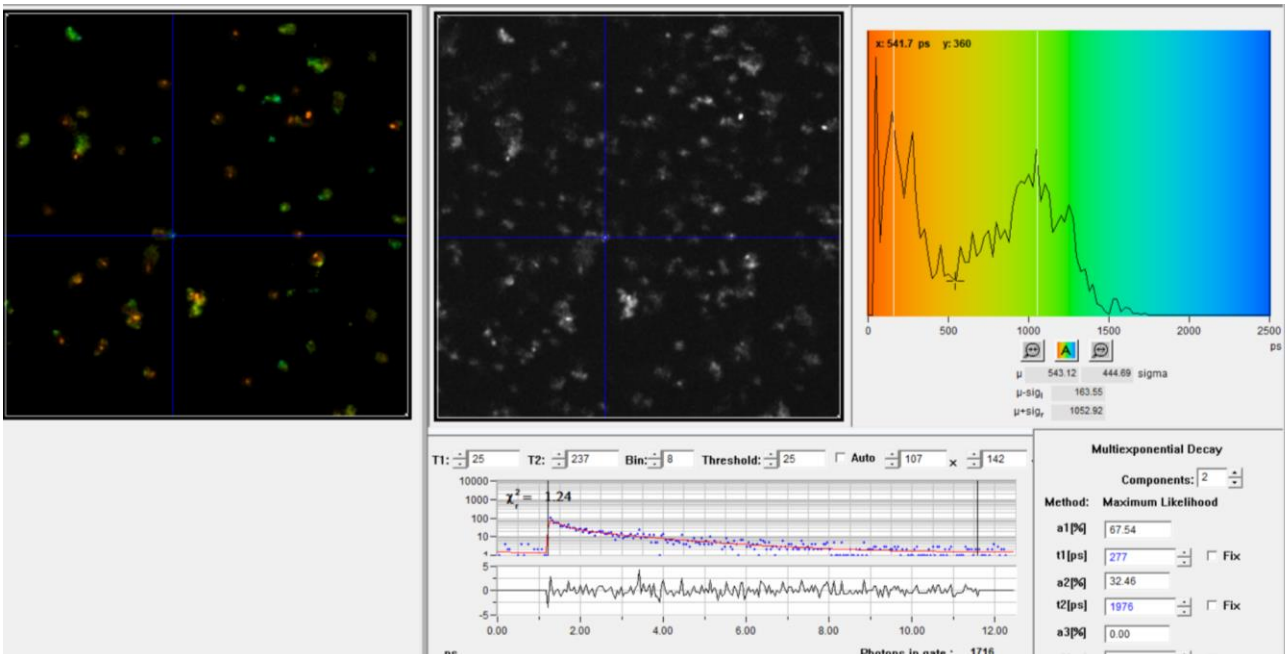

Figure S22. Alternative 2P FLIM experiments with luminescent particles (Eu,Dy:SrAlO), 2-photon 800 nm excitation, thin film deposited then dried from 1 mg/mL dispersion in H_2O. Top row, left to right: Lifetime mapping. Corresponding 2P emission intensity micrograph. Rainbow coloured chart corresponding to lifetime distributions and curve. Bottom row: 2P TCSPC Lifetime measurement in typical spots and corresponding fitting parameters.

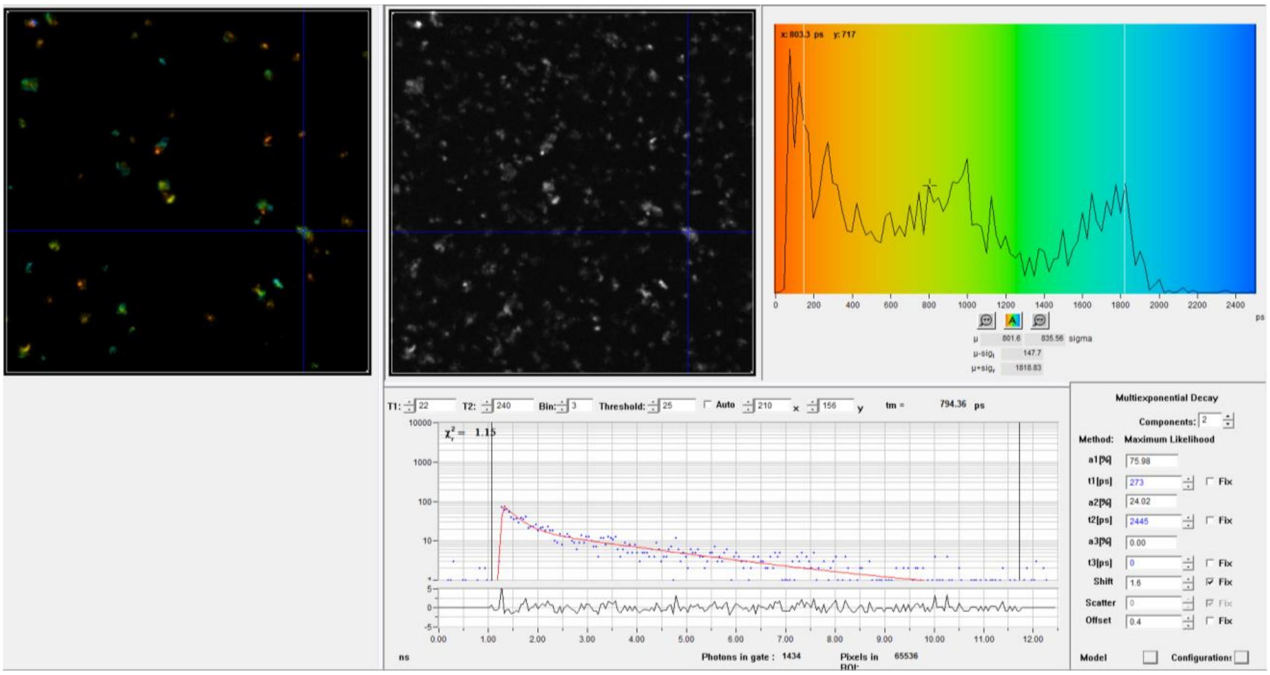

Figure S23. Alternative 2P FLIM experiments with luminescent particles (Eu,Dy:SrAlO), 800 nm excitation, thin film deposited then dried from 1 mg/mL dispersion in H_2O. Top row, left to right: Lifetime mapping. Corresponding 2P emission intensity micrograph. Rainbow coloured chart corresponding to lifetime distributions and curve. Bottom row: 2P TCSPC Lifetime measurement in typical spots and corresponding fitting parameters.

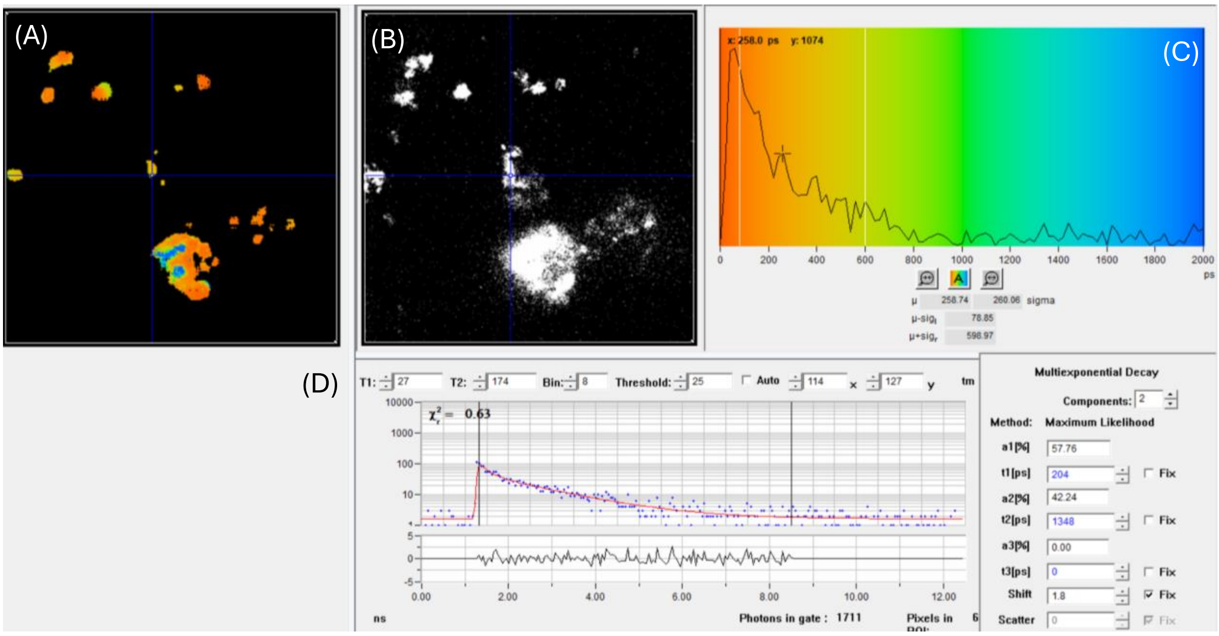

Figure S24. Alternative micrographs for a 2P FLIM experiment with luminescent particles (Eu,Dy:SrAlO), 800 nm excitation, thin film deposited then dried from 1 mg/mL dispersion in H_2O. Top row, left to right: (A): Lifetime mapping. (B): Corresponding 2P emission intensity micrograph. (C): Rainbow coloured chart corresponding to lifetime distributions and curve. (D): TCSPC Lifetime measurement in typical spot and corresponding fitting parameters.

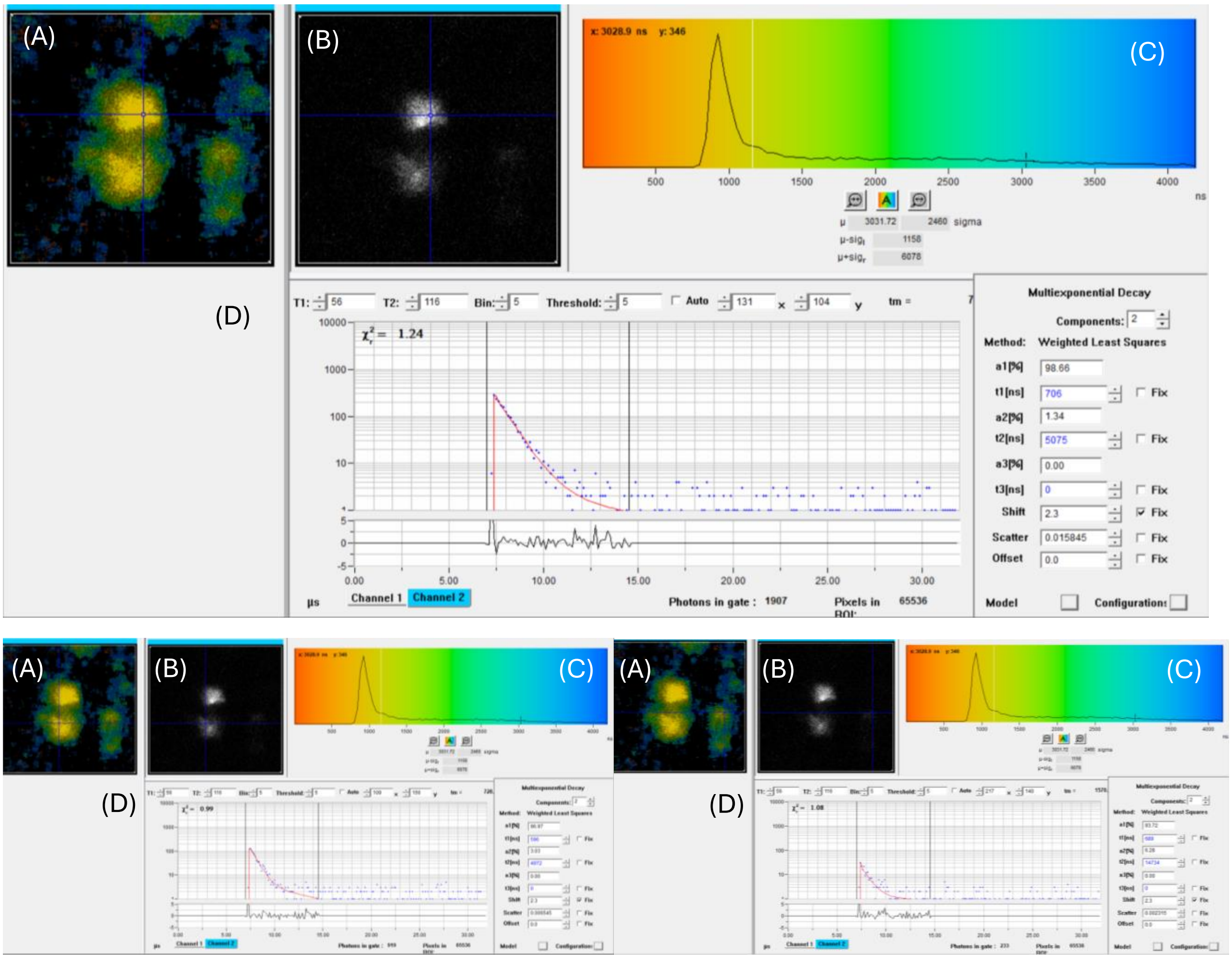

Figure S25. PLIM data recorded with luminescent particles (Eu,Dy:SrAlO), 400 nm excitation, thin film deposited then dried from 1 mg/mL dispersion in H_2O. Top row, left to right: (A): Lifetime mapping. (B): Corresponding 2P emission intensity micrograph. (C): Rainbow coloured chart corresponding to lifetime distributions and curve. (D): TCSPC Lifetime measurement in typical spot and corresponding fitting parameters.

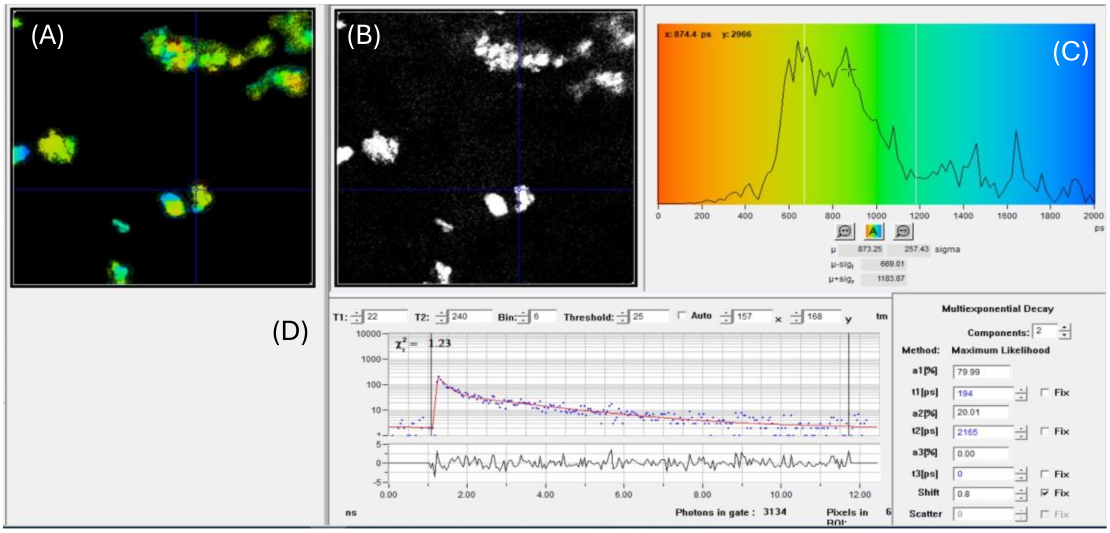

Figure S26. Alternative micrographs for a 2P FLIM experiment with core-shell dispersed particles (CeO_2@Eu,Dy:SrAlO) deposited on a borosilicate glass slide, 800 nm excitation. (A): Lifetime mapping. (B): Corresponding 2P emission intensity micrograph. (C): Rainbow coloured chart corresponding to lifetime distributions and curve. (D): Lifetime measurement in typical spot and corresponding fitting parameters.

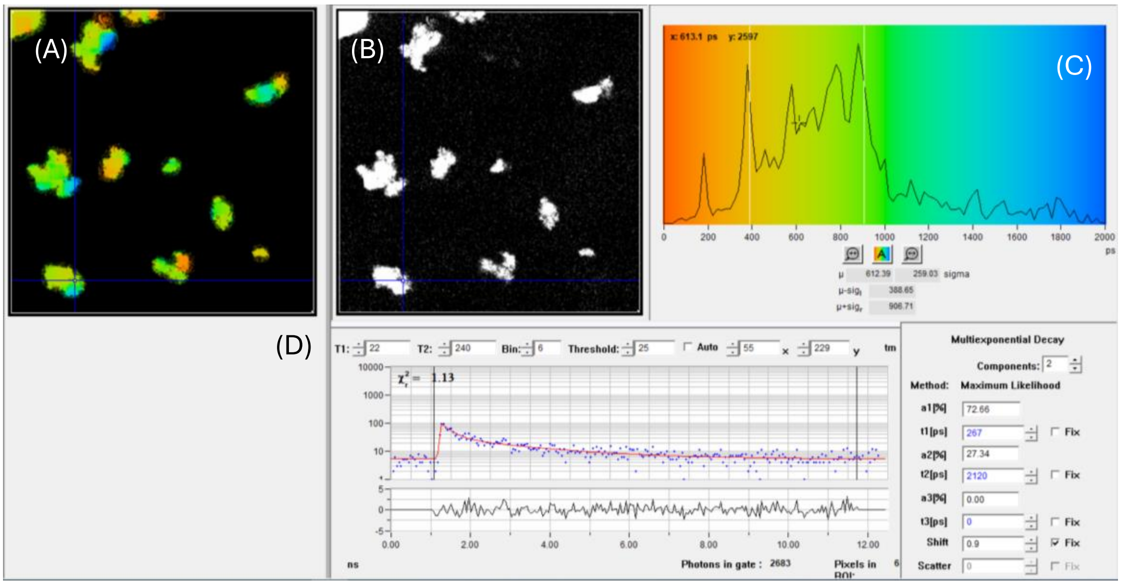

Figure S27. Alternative micrographs for a 2P FLIM experiment with core-shell particles (CeO_2@Eu,Dy:SrAlO), 800 nm excitation. (A): Lifetime mapping. (B): Corresponding 2P emission intensity micrograph. (C): Rainbow coloured chart corresponding to lifetime distributions and curve. (D): Lifetime measurement in typical spot and corresponding fitting parameters

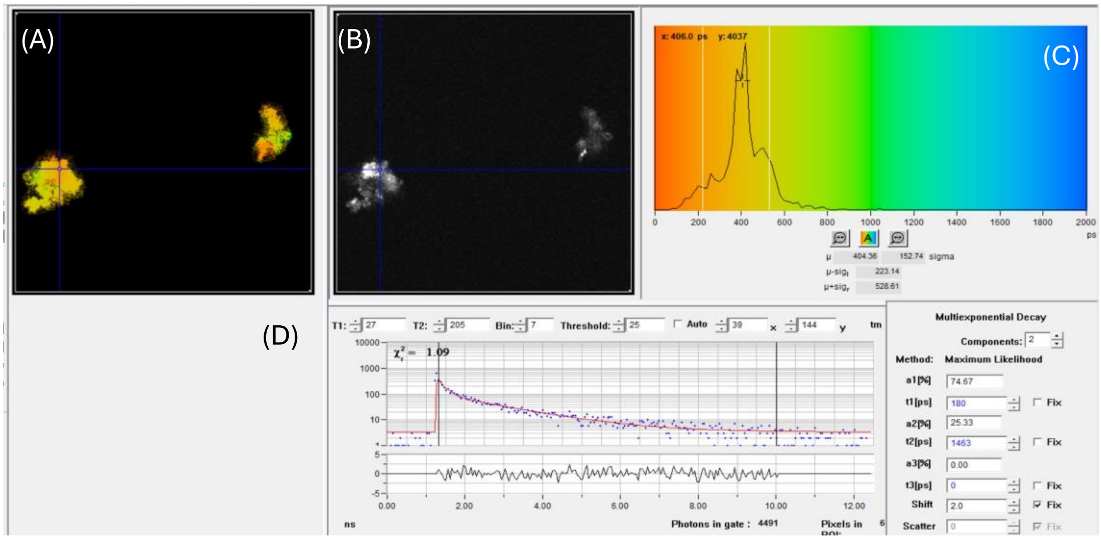

Figure S28. Alternative micrographs for a 2P FLIM experiment with core-shell particles (CeO_2@Eu,Dy:SrAlO), 800 nm excitation. (A): Lifetime mapping. (B): Corresponding 2P emission intensity micrograph. (C): Rainbow coloured chart corresponding to lifetime distributions and curve. (D): Lifetime measurement in typical spot and corresponding fitting parameters.

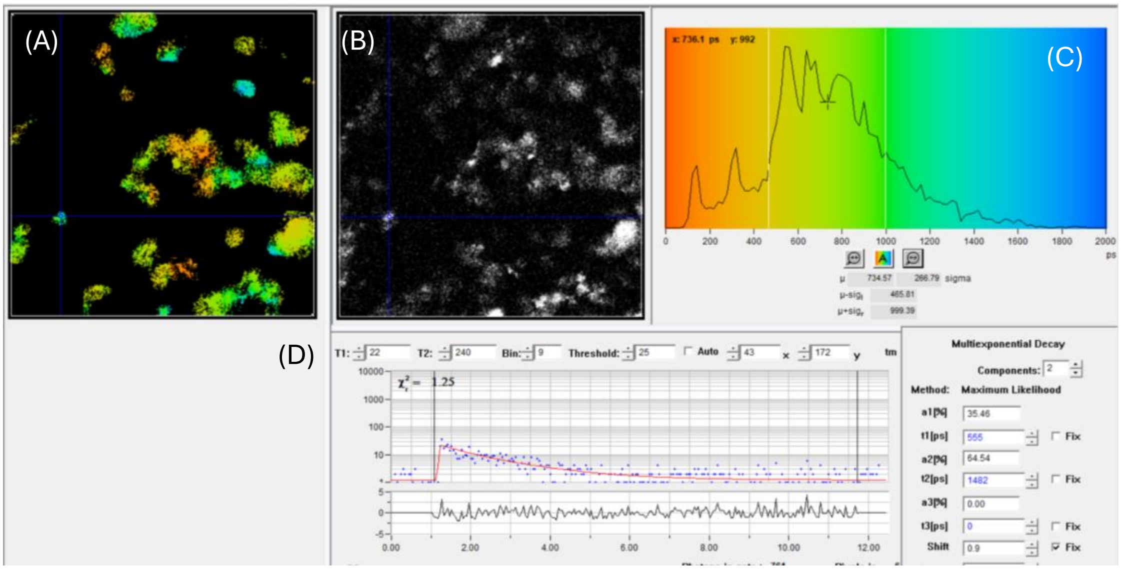

Figure S29. Alternative micrographs for a 2P FLIM experiment with core-shell particles (CeO_2@Eu,Dy:SrAlO), 800 nm excitation: (A) Lifetime mapping, (B) Corresponding 2P emission intensity micrograph, (C) Rainbow coloured chart corresponding to lifetime distributions and curve. (D): Lifetime measurement in random spot (blue cross lines in (A) and (B)) and corresponding fitting parameters.

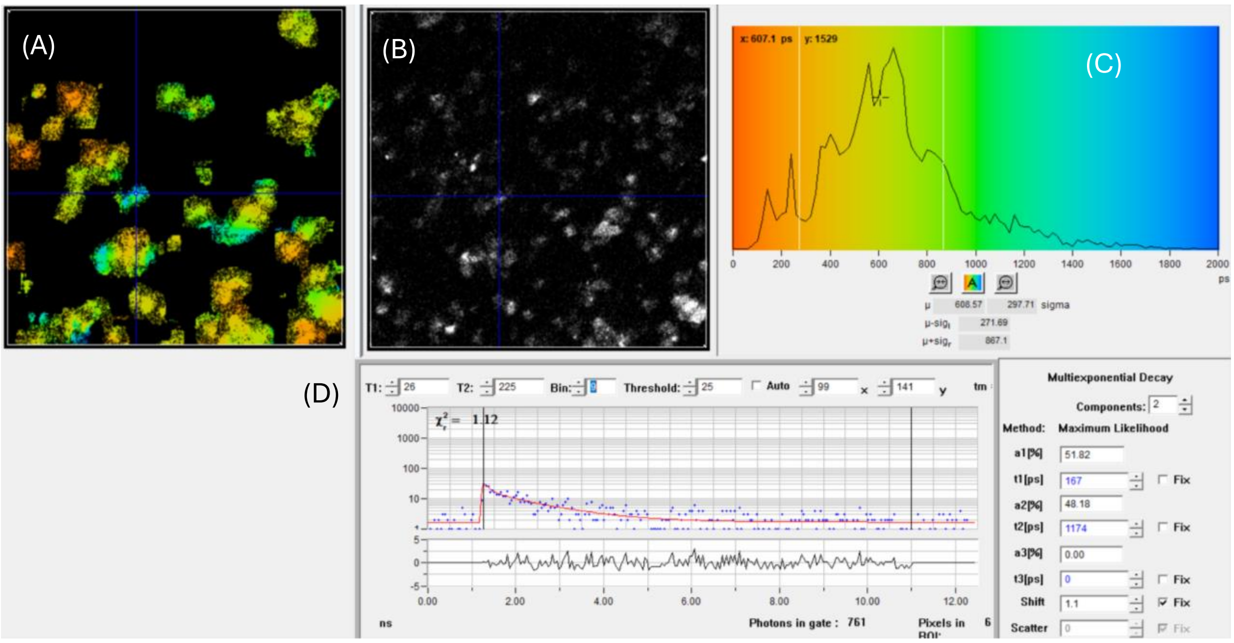

Figure S30. 2P FLIM experiment with core-shell particles (CeO_2@Eu,Dy:SrAlO), 800 nm excitation. (A): Lifetime mapping. (B): Corresponding 2P emission intensity micrograph. (C): Rainbow coloured chart corresponding to lifetime distributions and curve. (D): Lifetime measurement in typical spot and corresponding fitting parameters.

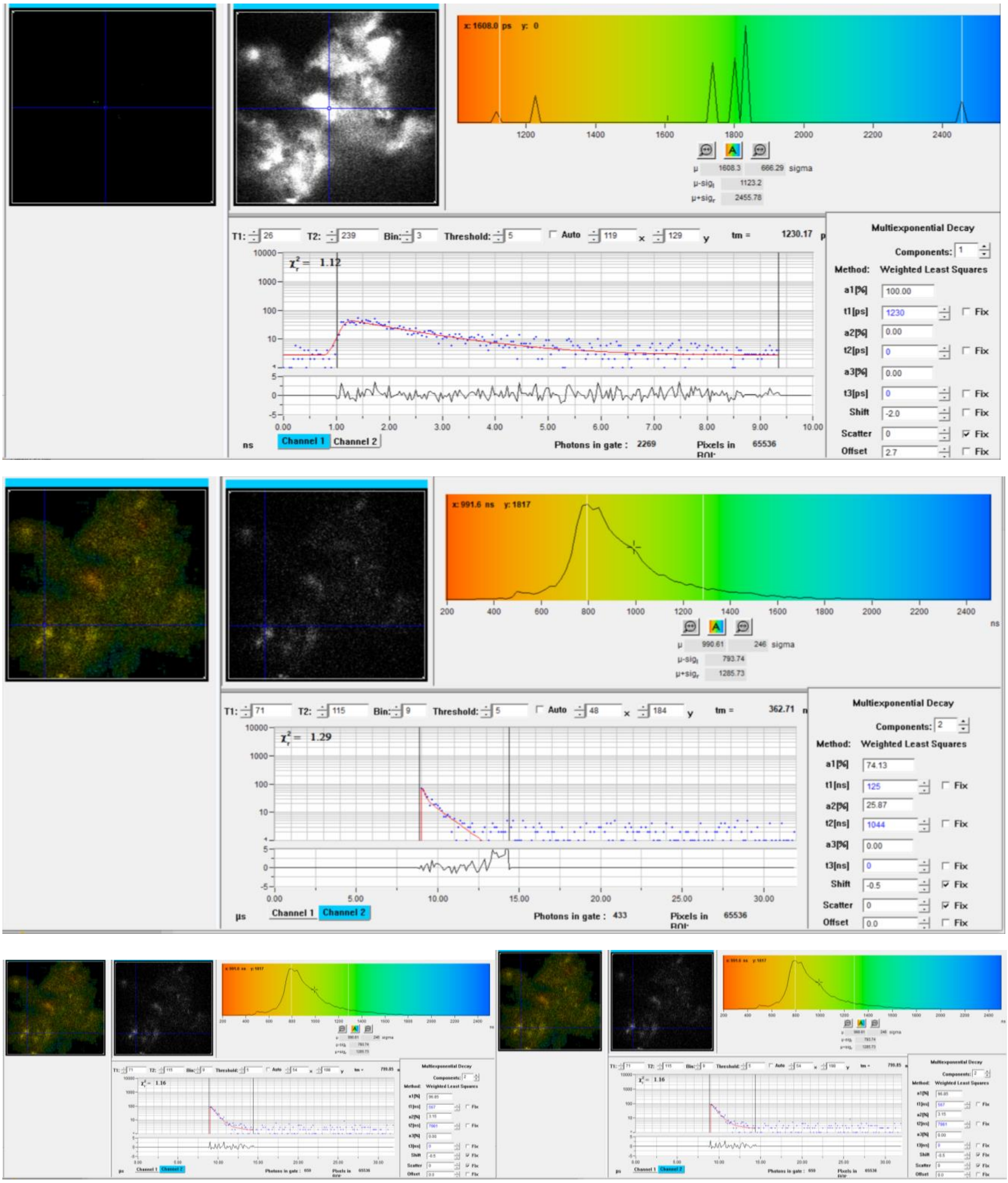

Figure S31. Correlated FLIM and PLIM measurements of particle films (400 nm excitation) for (CeO_2@Eu,Dy:SrAlO), Channel 1: Fluorescence Intensity channel (λ_{em} = 570–750 nm); Channel 2: PLIM intensity and lifetime map and associated profile distribution for core-shell structures.
Colours provide a direct correlation between the lifetime maps and the lifetime histograms and TCSPC data and fitting parameters are given in several different spots.

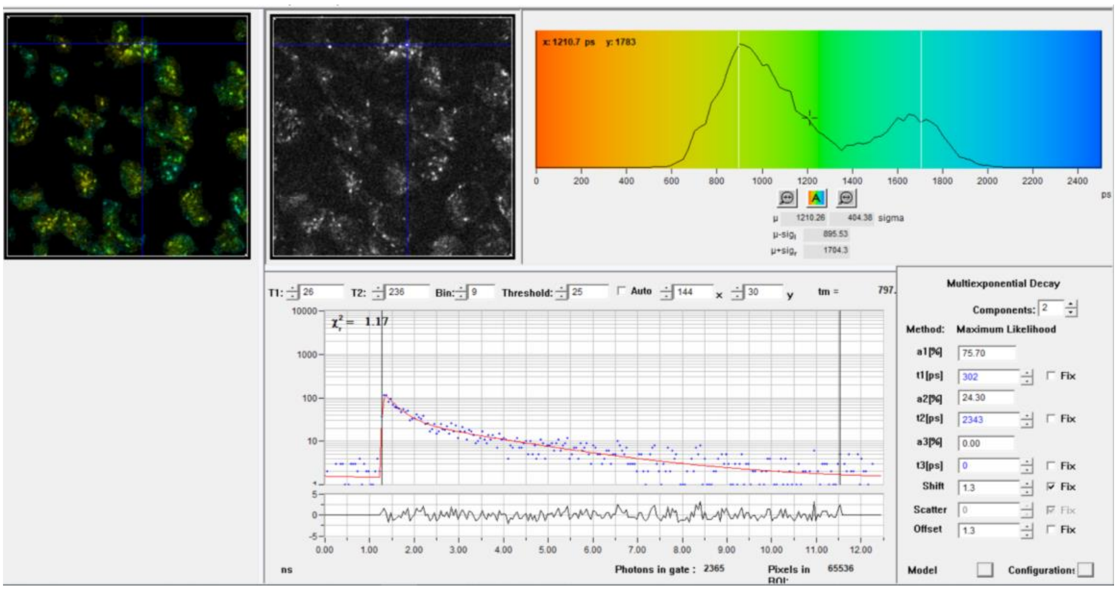

Figure S32. 2P FLIM luminescent particles (Eu,Dy:SrAlO) 1 mg/ml in living CHO cells, 15 min incubation, 37 ºC, 1% DMSO, excited at 800 nm excitation with a laser power 2.0 mW. Lifetime mapping. Corresponding 2P emission intensity micrograph. Rainbow coloured chart corresponding to lifetime distributions and curve. Raw data collected and residuals are also show.

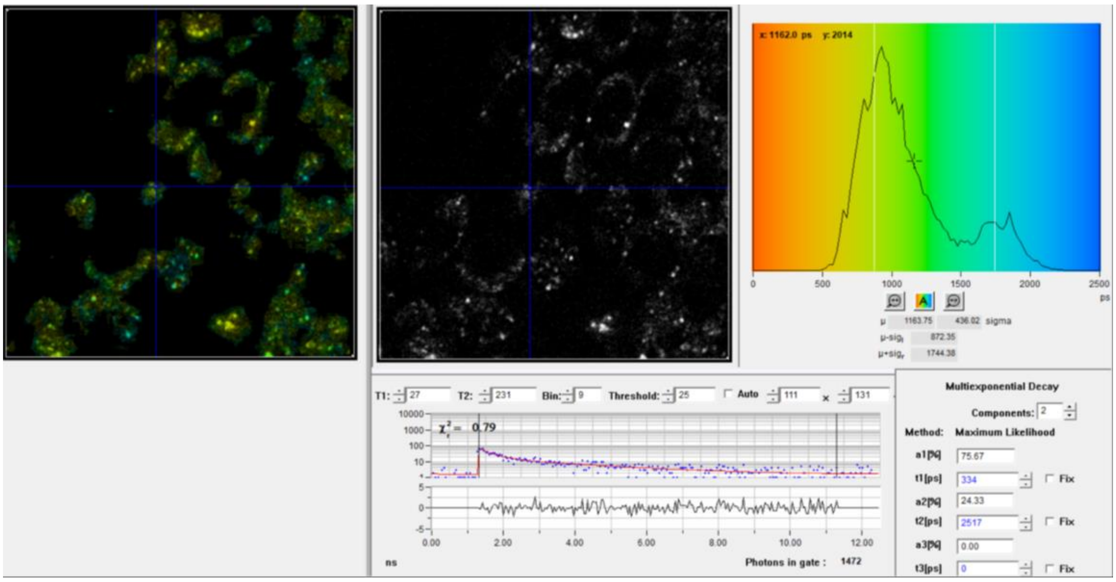

Figure S33. 2P FLIM luminescent particles (Eu,Dy:SrAlO) 1 mg/ml in living CHO cells, 15 min incubation, 37 ºC, 1% DMSO, excited at 800 nm excitation with a laser power 2.0 mW. Lifetime mapping. Corresponding 2P emission intensity micrograph. Rainbow coloured chart corresponding to lifetime distributions and curve. Raw data collected and residuals are also show.

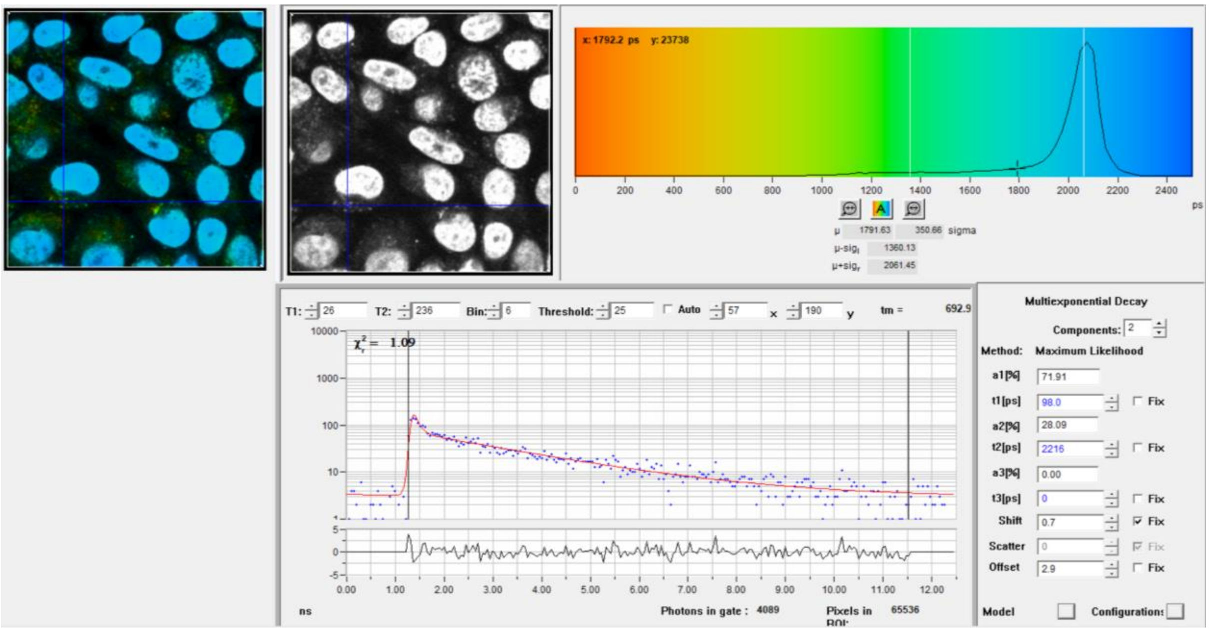

Figure S34. 2P FLIM luminescent particles (Eu,Dy:SrAlO),) 1 mg/ml in living CHO cells, 15 min incubation, 37 ºC, 1% DMSO, and NucBlue, (515 nm filter, 800 nm excitation) with a laser power 2.0 mW. Lifetime mapping. Corresponding 2P emission intensity micrograph. Rainbow coloured chart corresponding to lifetime distributions and curve. Raw data collected and residuals are also show.

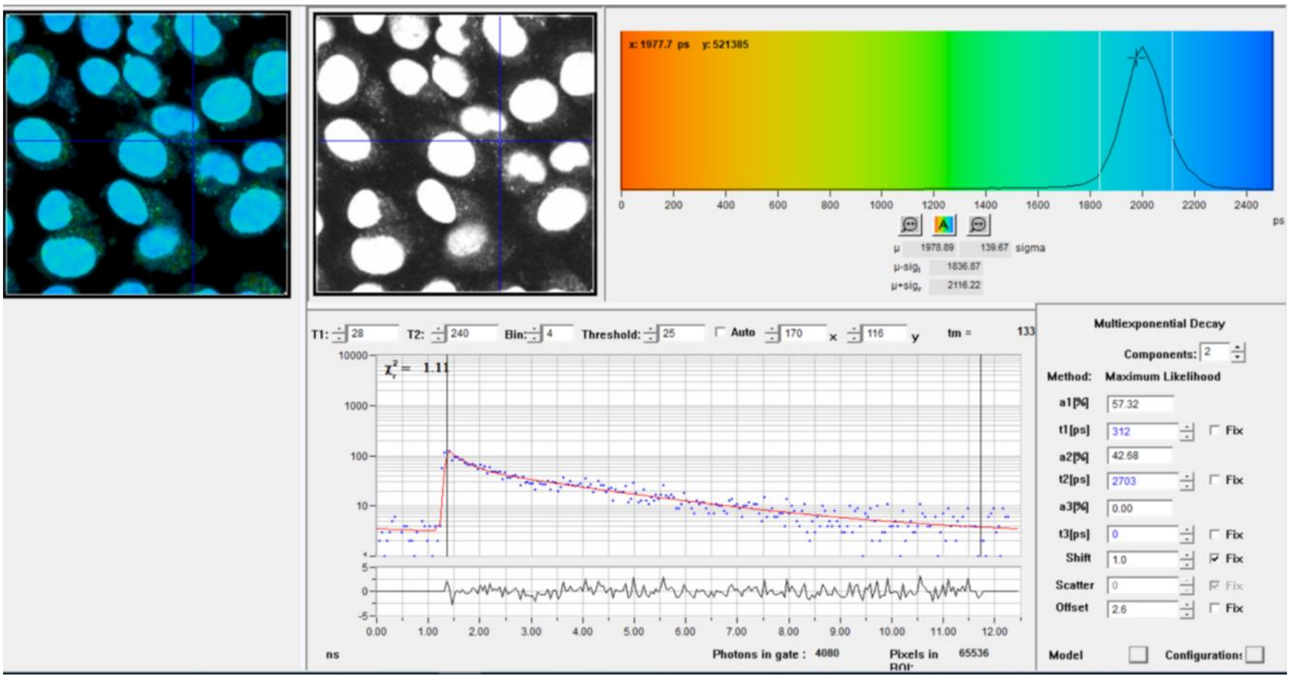

Figure S35. 2P FLIM luminescent particles (CeO_2@Eu,Dy:SrAlO), 1 mg/ml in living CHO cells, 15 min incubation, 37 ºC, 1% DMSO, and NucBlue, excited at 800 nm excitation with a laser power 2.0 mW. Lifetime mapping. Corresponding 2P emission intensity micrograph. Rainbow coloured chart corresponding to lifetime distributions and curve. Raw data collected and residuals are also show.

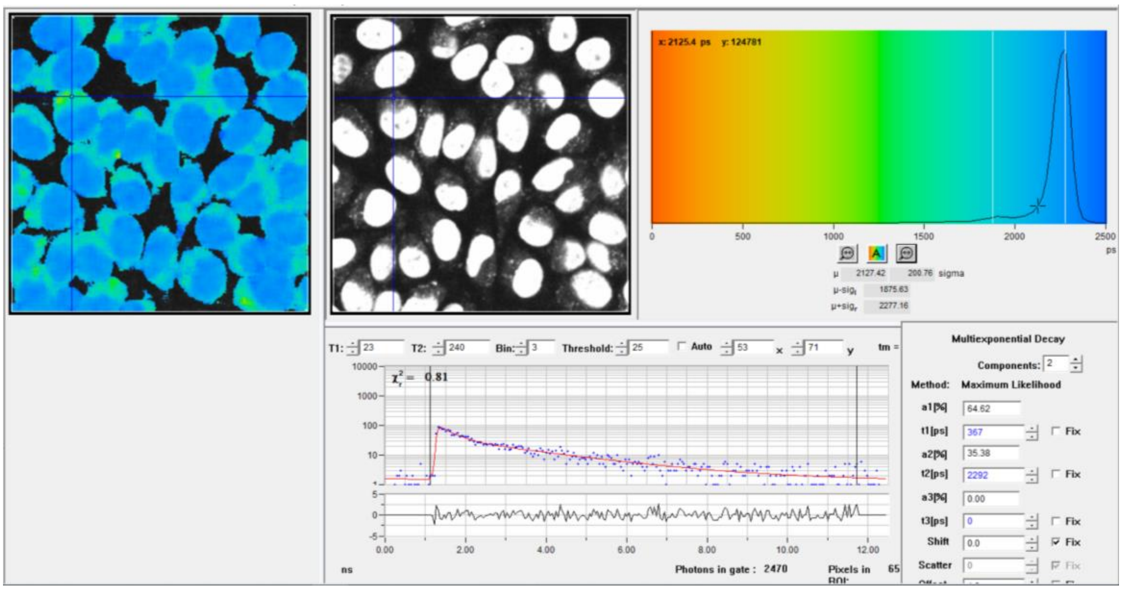

Figure S36. 2P FLIM luminescent particles (Eu,Dy:SrAlO) 1 mg/ml in living CHO cells, 15 min incubation, 37 ºC, 1% DMSO, excited at 800 nm excitation with a laser power 2.0 mW. Lifetime mapping. Corresponding 2P emission intensity micrograph. Rainbow coloured chart corresponding to lifetime distributions and curve. Raw data collected and residuals are also show.

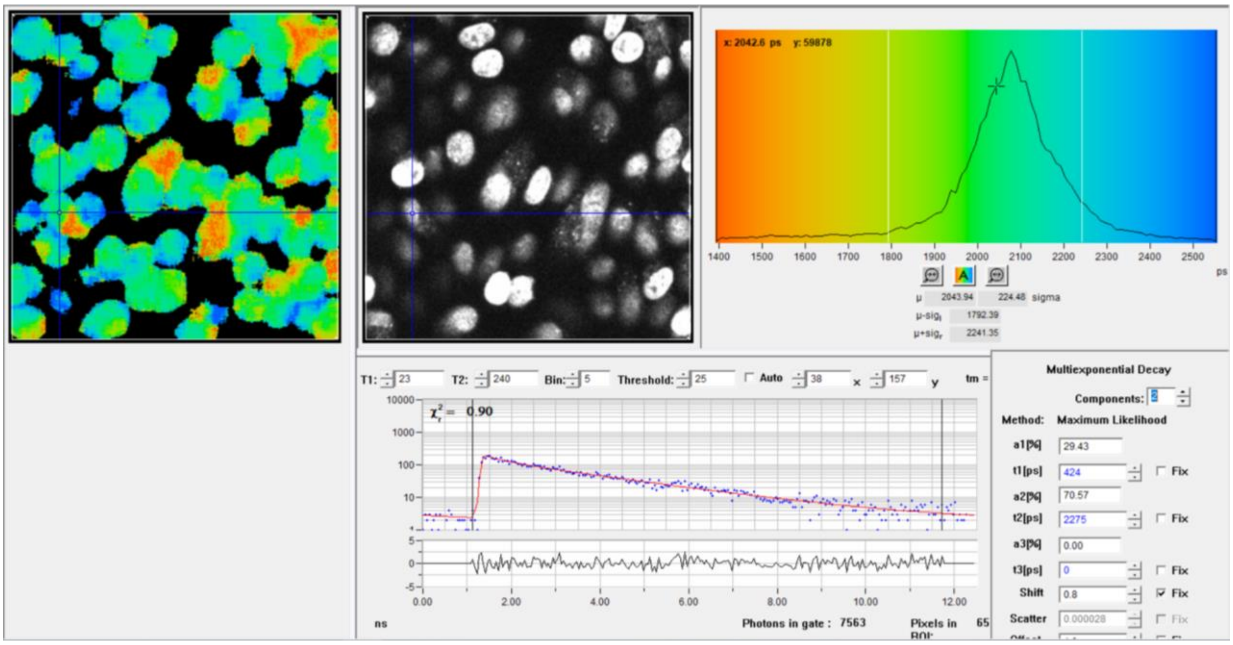

Figure S37. 2P FLIM luminescent particles (Eu,Dy:SrAlO), 1 mg/ml in living CHO cells, 15 min incubation, 37 ºC, 1% DMSO, excited at 800 nm excitation (filter set 515 nm) with a laser power 2.0 mW. Lifetime mapping. Corresponding 2P emission intensity micrograph. Rainbow coloured chart corresponding to lifetime distributions and curve. Raw data collected and residuals are also shown.

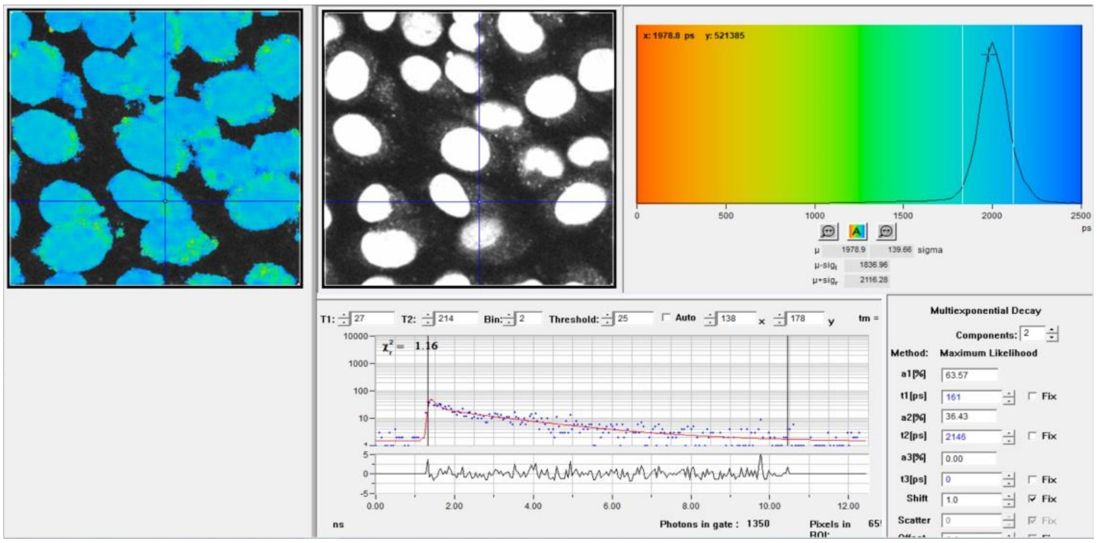

Figure S38. 2P FLIM luminescent particles (Eu,Dy:SrAlO) 1 mg/ml in living CHO cells, 15 min incubation, 37 ºC, 1% DMSO, excited at 800 excitation (filter set 495 nm) with a laser power 2.0 mW. Lifetime mapping. Corresponding 2P emission intensity micrograph. Rainbow coloured chart corresponding to lifetime distributions and curve.

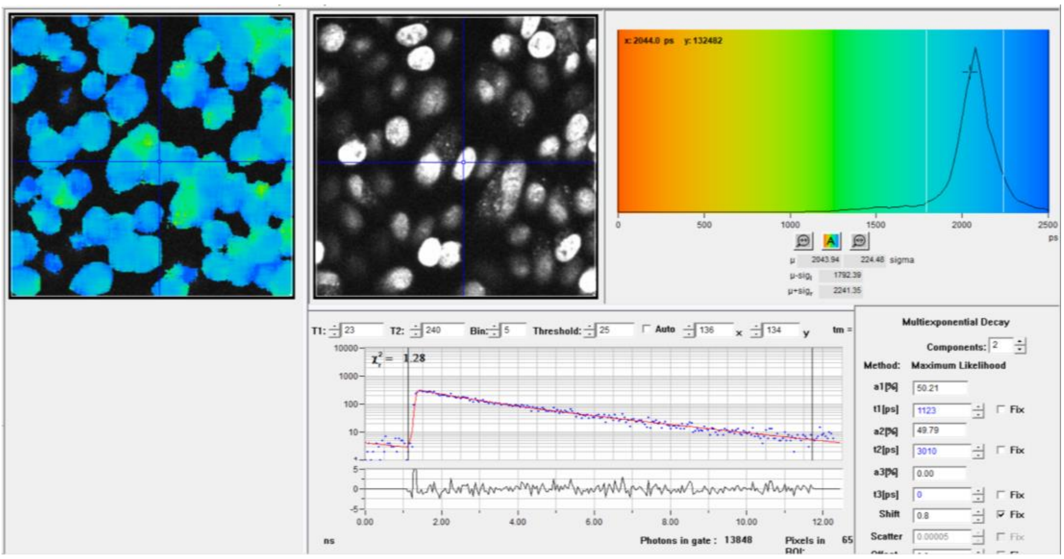

Figure S39. 2P FLIM luminescent particles (Eu,Dy:SrAlO) 1 mg/ml in living CHO cells, 15 min incubation, 37 ºC, 1% DMSO, excited at 800 nm excitation with a laser power 2.0 mW. Lifetime mapping. Corresponding 2P emission intensity micrograph. Rainbow coloured chart corresponding to lifetime distributions and curve.

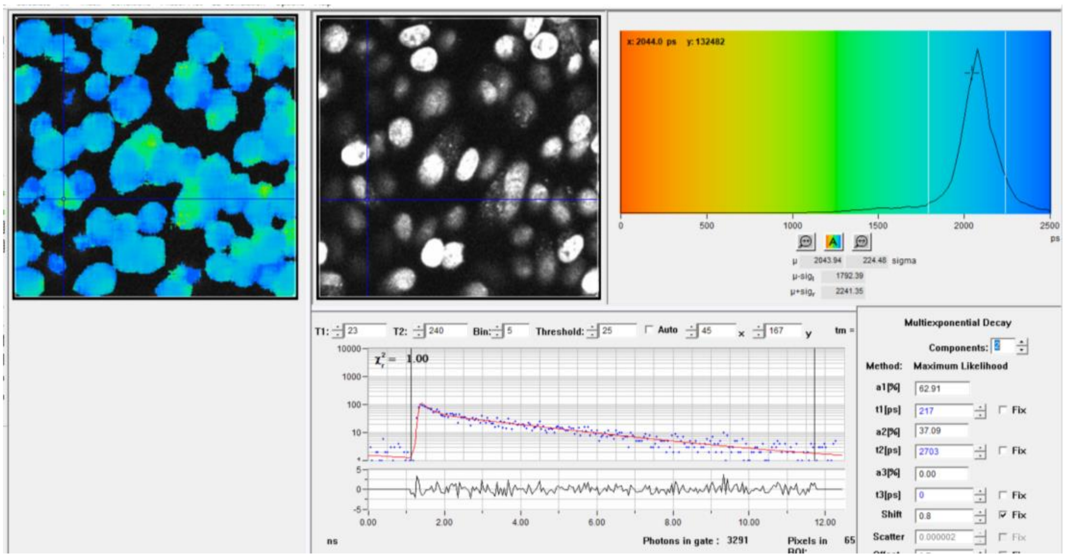

Figure S40. 2P FLIM luminescent particles (Eu,Dy:SrAlO) 1 mg/ml in living CHO cells, 15 min incubation, 37 ºC, 1% DMSO, excited at 800 nm excitation with a laser power 2.0 mW. Lifetime mapping. Corresponding 2P emission intensity micrograph. Rainbow coloured chart corresponding to lifetime distributions and curve (Alternative spot chosen)

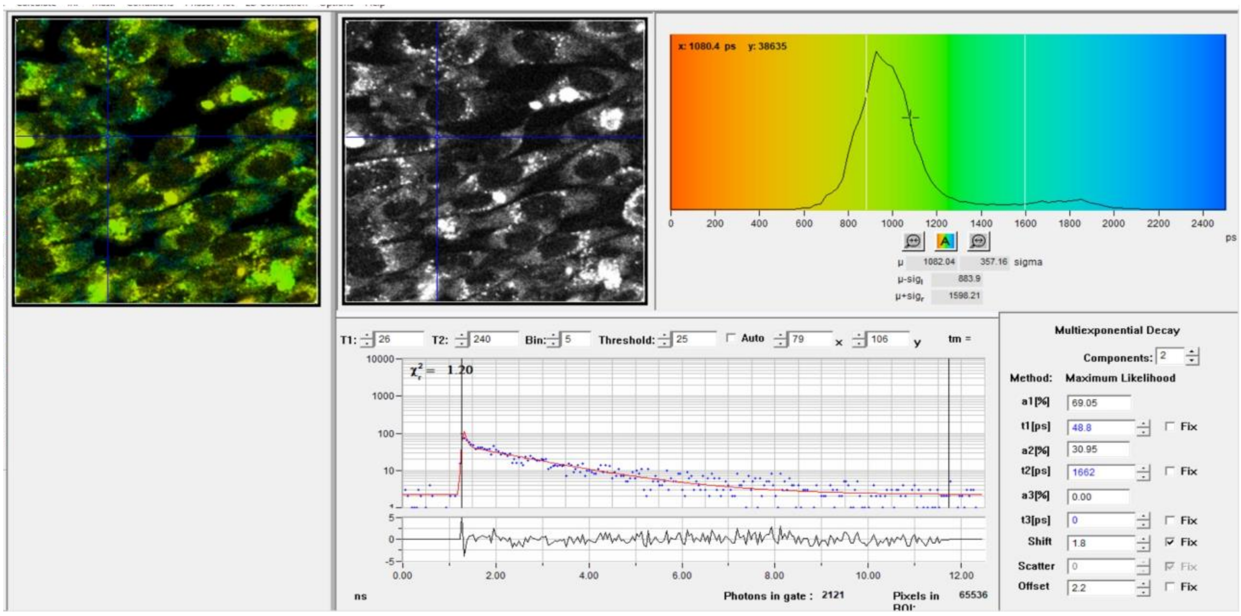

Figure S41. 2P FLIM luminescent particles (CeO_2@Eu,Dy:SrAlO) 1 mg/ml in living CHO cells, 2 h incubation, 37 ºC, 1% DMSO, excited at 800 nm excitation (515 nm filter) with a laser power 2.0 mW. Lifetime mapping. Corresponding 2P emission intensity micrograph. Rainbow coloured chart corresponding to lifetime distributions and curve.

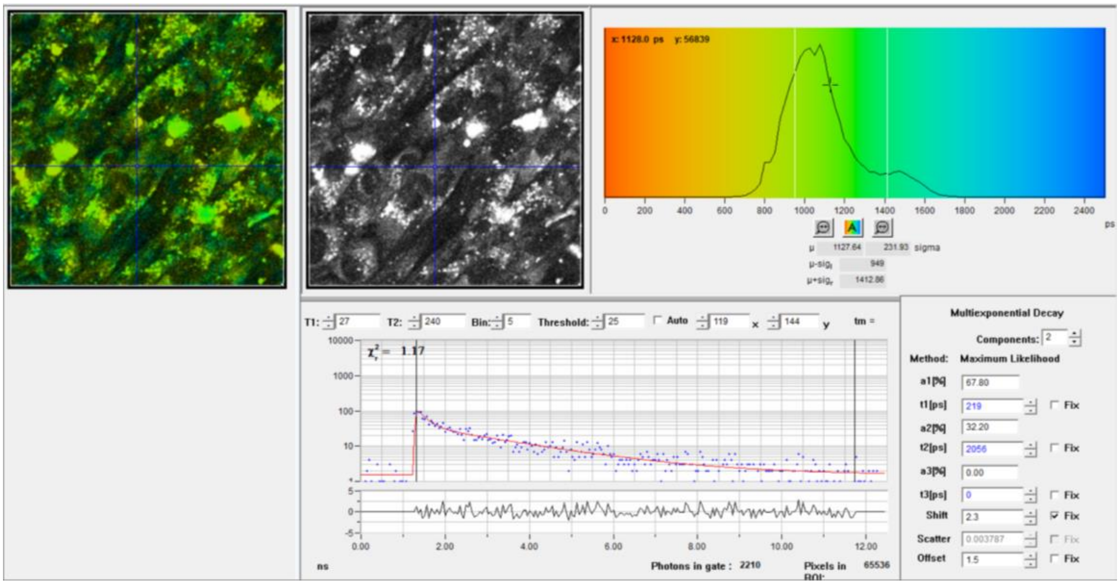

Figure S42. 2P FLIM luminescent particles (CeO_2@Eu,Dy:SrAlO) 1 mg/ml in living CHO cells, 2 h incubation, 37 ºC, 1% DMSO, excited at 800 nm excitation (515 nm filter) with a laser power 2.0 mW. Lifetime mapping. Corresponding 2P emission intensity micrograph. Rainbow coloured chart corresponding to lifetime distributions and curve.

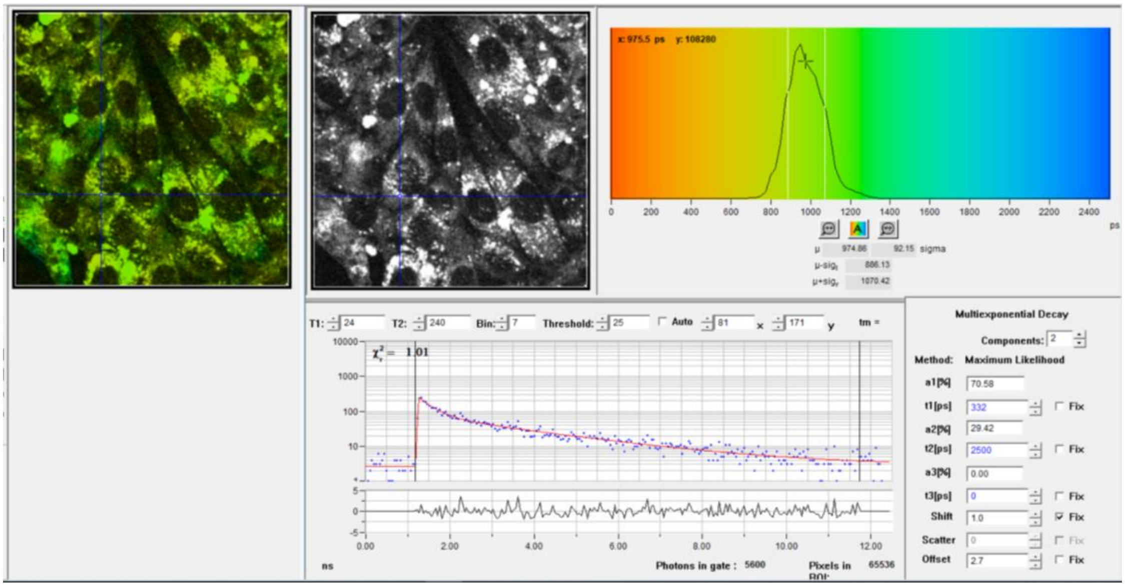

Figure S43. 2P FLIM luminescent particles (CeO_2@Eu,Dy:SrAlO) 1 mg/ml in living CHO cells, 15 h incubation, 37 ºC, 1% DMSO, 800 nm excitation (515 nm filter) with a laser power 2.0 mW. Lifetime mapping. Corresponding 2P emission intensity micrograph. Rainbow coloured chart corresponding to lifetime distributions and curve.

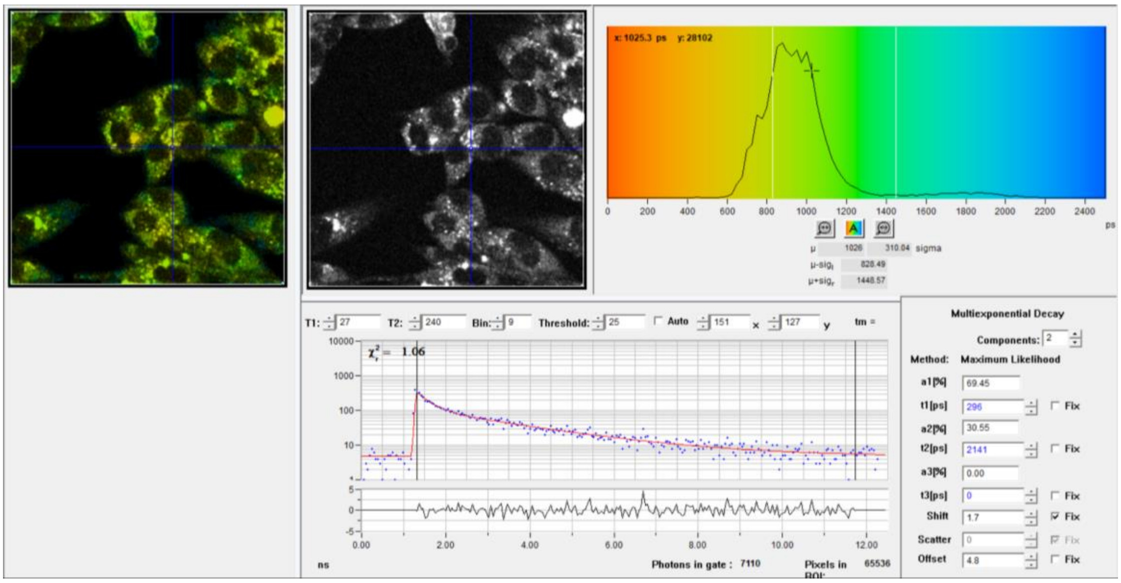

Figure S44. 2P FLIM luminescent particles (CeO_2@Eu,Dy:SrAlO) 1 mg/ml in living CHO cells, 15 h incubation, 37 ºC, 1% DMSO, excited at 800 nm excitation with a laser power 2.0 mW. Lifetime mapping. Corresponding 2P emission intensity micrograph. Rainbow coloured chart corresponding to lifetime distributions and curve.

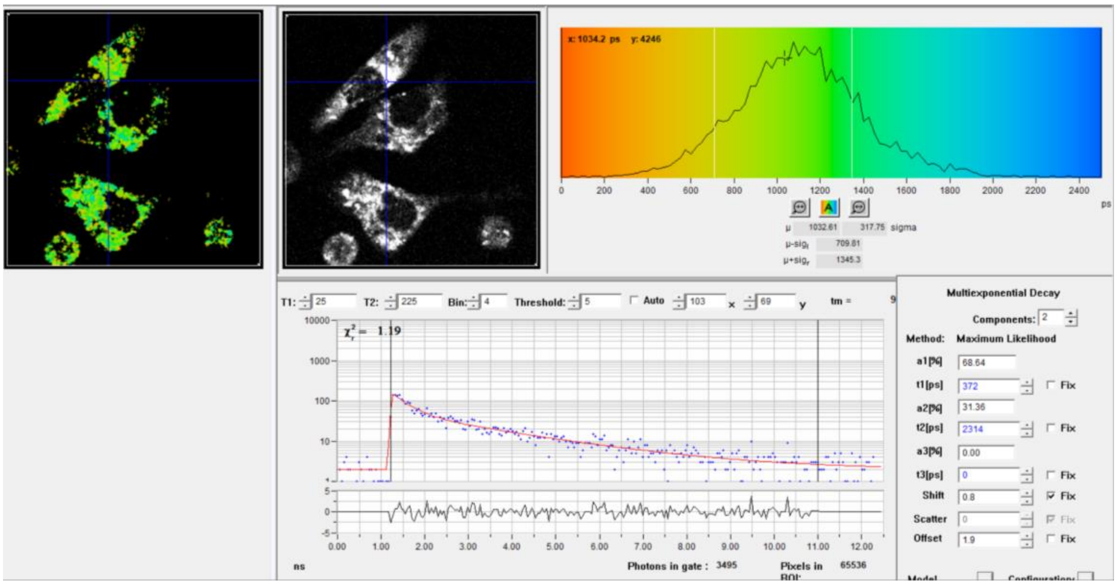

Figure S45. 2P FLIM luminescent particles (CeO_2@Eu,Dy:SrAlO) 1 mg/ml in living PC3 cells, 15 h incubation, 37 ºC, 1% DMSO, 800 nm excitation (515 nm filter) with a laser power 2.0 mW. Lifetime mapping. Corresponding 2P emission intensity micrograph. Rainbow coloured chart corresponding to lifetime distributions and curve.

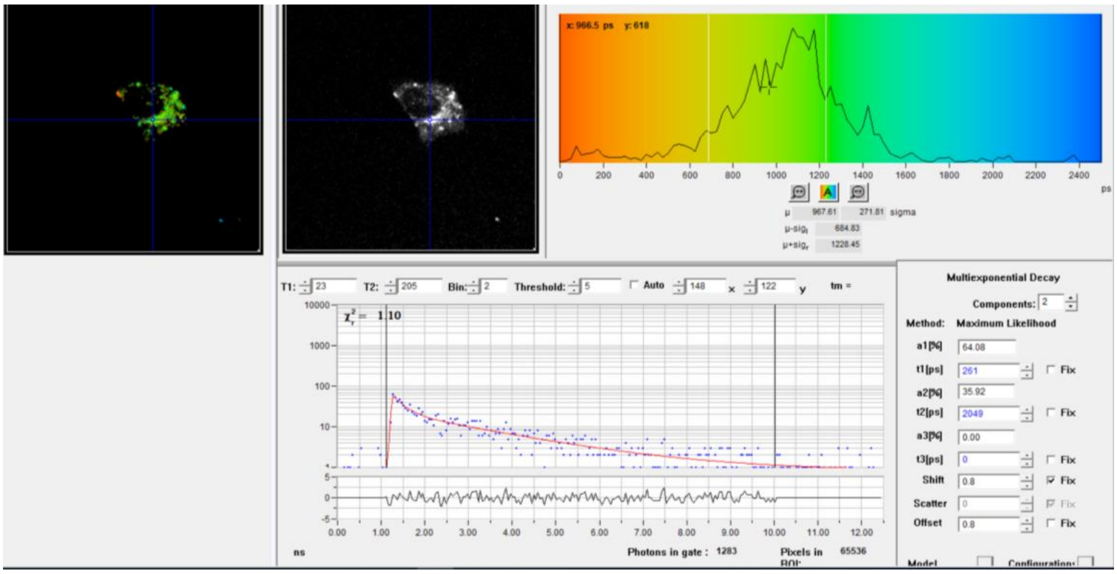

Figure S46. 2P FLIM luminescent particles (CeO_2@Eu,Dy:SrAlO) 1 mg/ml in living PC3 cells, 15 h incubation, 37 ºC, 1% DMSO, excited at 800 nm excitation with a laser power 2.0 mW. Lifetime mapping. Corresponding 2P emission intensity micrograph. Rainbow coloured chart corresponding to lifetime distributions and curve.

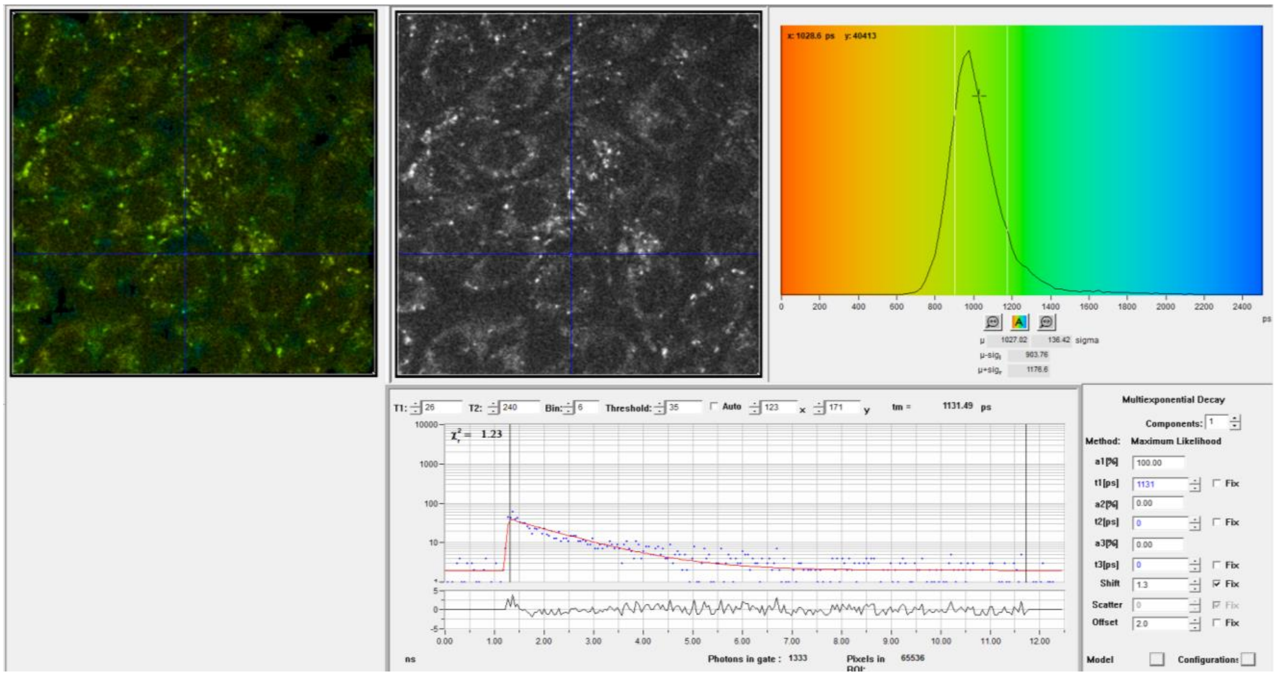

Figure S47. Control Experiments: untreated cells under 800 nm excitation: 2P FLIM of living CHO cells, 15 min incubation, 37 ºC, 1% DMSO, excited at 800 nm excitation with a laser power 2.0 mW. Lifetime mapping with corresponding 2P emission intensity micrograph. Rainbow coloured chart corresponding to lifetime distributions and curve.

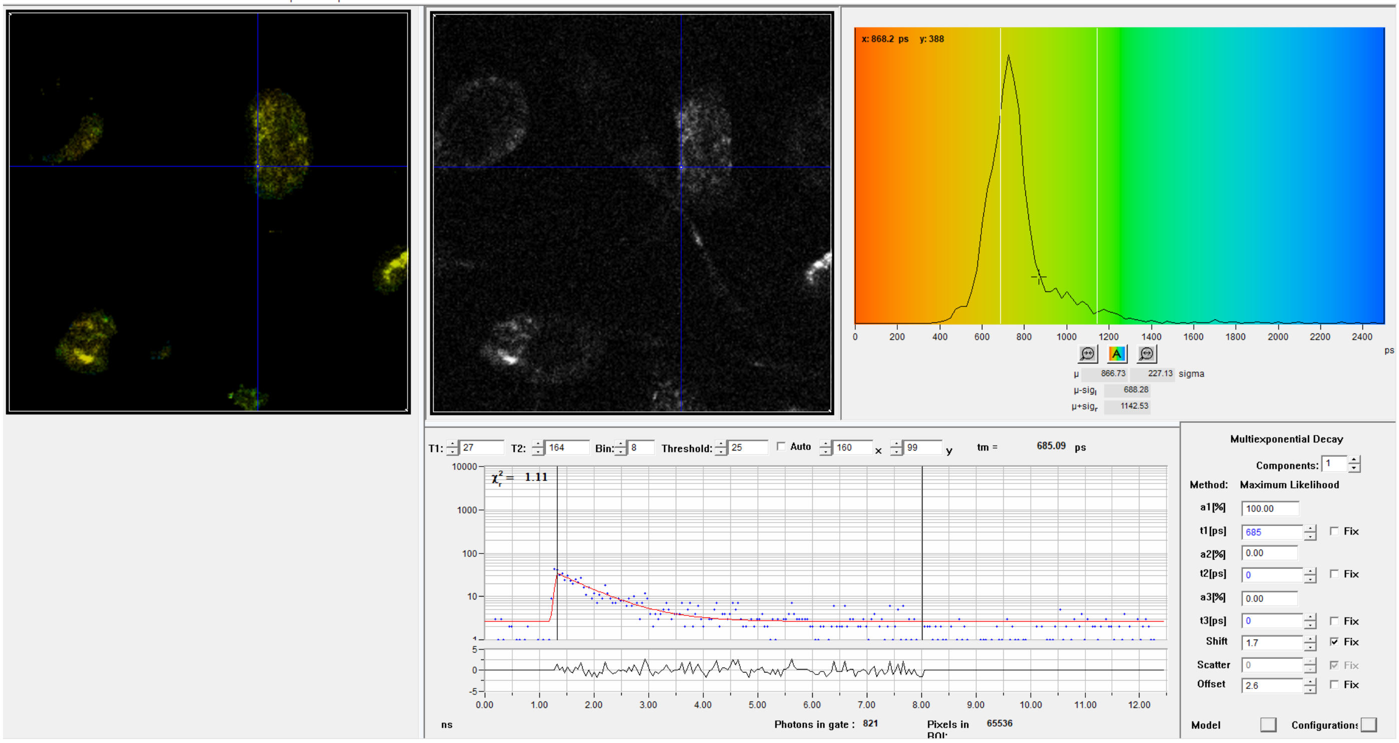

Figure S48. Control Experiments: untreated cells under 800 nm excitation: 2P FLIM of living PC3 cells, 15 min incubation, 37 ºC, 1% DMSO, excited at 800 nm excitation with a laser power 2.0 mW. Lifetime mapping. Corresponding 2P emission intensity micrograph. Rainbow coloured chart corresponding to lifetime distributions and curve.

5. General Cell culturing methods for fluorescence imaging

Chinese hamster Ovary cells (CHO) and Prostate cancer cells, PC-3 line, were purchased from American type culture collection (ATCC). Cells were grown as monolayers in T75 tissue culture flasks and cultured in Roswell Park Memorial Institute medium (RPMI), 1% L-glutamine (200 mM), 0.5% penicillin/streptomycin (10 000 IU mL-1/10 000 mg mL-1). Cells were cultured at 37 °C in a humidified atmosphere of 5% CO_2 in air and split once 70% confluence had been reached, using the corresponding cell medium. All steps were performed in absence of phenol red. Once cells reached more than 70% confluence, the supernatant containing dead cell matter and excess protein was aspirated. The live adherent cells were then washed with 10 mL of phosphate buffer saline solution twice to remove any remaining media. Cells were incubated in 3 mL of trypsin solution (0.25% trypsin) for 5 to 7 min at 37 °C.

After trypsinisation, 6 mL of medium containing 10% serum medium was added to inactivate the trypsin and the solution was centrifuged for 5 min (1000 rpm, 25 °C). The supernatant liquid was aspirated and 5 mL of serum medium was added to the cell matter left behind. Cells were counted using a haemocytometer and then seeded as appropriate.

For microscopy, cells were seeded into glass-bottomed Petri dishes and incubated for 24 h to ensure adhesion. Cells were plated in 35 mm uncoated 1.5 mm thick glass-bottomed dishes as 3 × 105 cells per dish and incubated for at least 24 h prior to imaging experiment. Once cells attached firmly, cells were washed with 990 µL Hank's Balanced Salt Solution (HBSS) five times and refilled with 990 µL of serum-free medium (SFM), then in each case, an aliquot of 10 µL of the nanoparticulate material (generally as 1 mg/mL stock dispersion in DMSO) was added. Cells were incubated with compounds for 15 minutes, 1 h, 6h or over-night at 37 °C, or longer, as required. Afterwards, cells were washed with 990 µL Hank's Balanced Salt Solution (HBSS) three times to rinse any remaining probe traces from the medium and 990 µL of SFM was added.

Once the cell dish was ready for the single photon confocal fluorescence imaging, cells were excited at 405 nm, 488 nm and 561 nm wavelength, then at each wavelength, there were five images captured namely a merged image, image between 420 and 480 nm wavelength, image between 516 and 530 nm wavelength, image between 615 and 650 nm wavelength and Differential Interference Contrast (DIC) image.

6. Cellular Viability Tests

Standard MTT assays of PC-3 cells treated with composite were performed in order to investigate the effect of the Eu,Dy:SrAlO alone, and post CeO_2 encapsulation giving rise to CeO_2@ Eu,Dy:SrAlO the cellular viability. Two different batches of ceria coated platelets were tested and the results demonstrate that all the nanoplatelet aterails tested were biocompatible and the encapsulation of Eu,Dy:SrAlO within a ceria shell improves the in vitro biocompatibility.

Normalised cell viability was evaluated in PC-3 cells treated with 1pg/mL – 20 µg/mL Eu,Dy:SrAlO and CeO_2@ Eu,Dy:SrAlO. Control cells seed and grown for 72 hours at 37 ºC, cells treated with either Eu,Dy:SrAlO and CeO_2@ Eu,Dy:SrAlO were incubated for 72 hours at 37 ºC, 5 mg/mL MTT reagent was incubated for 3 hours. Error bars stand for standard error calculated from the twelve repeats.

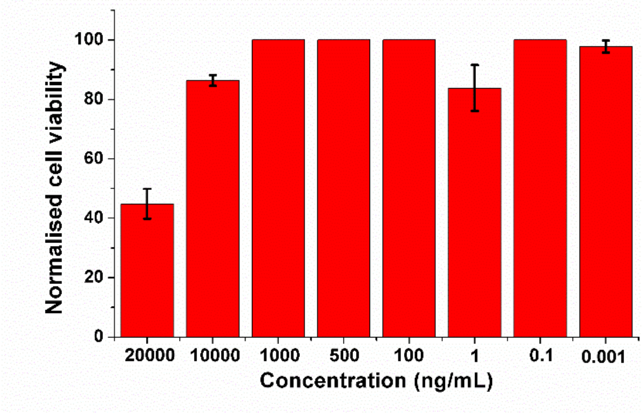

(a)

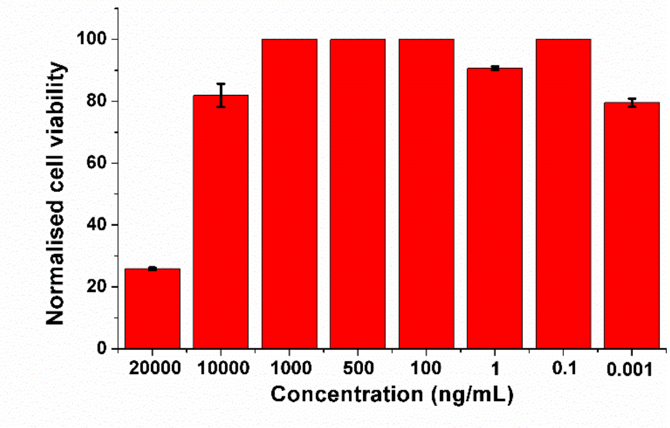

(b)

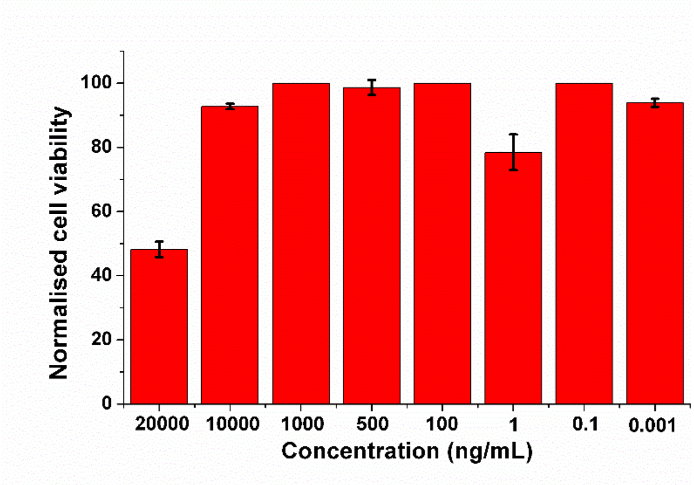

(c)

Figure S49. Cytotoxicity evaluation by MTT assays in PC3 cells treated with a) uncoated Eu,Dy:SrAlO and b,c) CeO_2@Eu,Dy:SrAlO (Batch 1 and Batch 2), with concentrations ranging from 0.001 ng/mL to 20000 ng/mL over 72 hours.
